# Quantifying the Biodegradation of Water‐Soluble Polymer Mixtures with Diffusion NMR Spectroscopy

**DOI:** 10.1002/anie.202514235

**Published:** 2025-09-26

**Authors:** Louisa T. Brenninkmeijer, Jacob L. Golding, Arianna Brandolese, Melanie M. Britton, Andrew P. Dove

**Affiliations:** ^1^ School of Chemistry University of Birmingham, Molecular Sciences Building Edgbaston B15 2TT UK

**Keywords:** Biodegradation, Diffusion NMR Spectroscopy, Hydrolysis, Polymer mixtures, Water‐soluble polymers

## Abstract

Polymers in liquid formulations result in 36 million tons of waste each year. It is estimated that 13% of these polymers directly enter, and accumulate in, natural environments, however, their fate is poorly understood; in part as a consequence of challenges in characterizing how the polymers biodegrade. Multiple analytical techniques have been used to quantify polymer biodegradation but require extensive sample preparation and can only measure one species accurately at a time, inhibiting the measurement of water‐soluble polymer mixtures. Here, we report the application of diffusion nuclear magnetic resonance spectroscopy as an alternative method to enable the facile monitoring of polymer biodegradation. This technique uniquely aids the understanding of biodegradation mechanisms, by measuring chemical as well as molar mass changes, concurrently, for both the polymer and degradation products. Furthermore, the ability to detect and measure the molar mass of multiple separate species enables the measurement of simultaneous biodegradation of polymer mixtures, including polymers with different chemical structures but the same molar mass.

## Graphical abstract

The fate of polymers in liquid formulations in natural environments has not been extensively researched, in part as a result of incompatible analytical techniques. We show that diffusion NMR spectroscopy can be used to quantify the simultaneous biodegradation of multiple water‐soluble polymers with limited sample preparation, measuring the molar mass, and chemical composition of multiple species with no molar mass restriction.

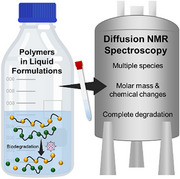

## Introduction

Polymers in liquid formulations (PLFs) have a global market value of $1 trillion and are used extensively as additives in applications as diverse as home and personal care, agrochemicals and water treatment formulations, among many other areas.^[^ ^1^, ^2^ ^]^ Despite their importance in enhancing product performance, there is a growing concern surrounding their fate after they have been discarded into waste streams.^[^ ^3^, ^4^, ^5^, ^6^ ^]^ Current PLFs, such as polyacrylates, are largely constructed from petroleum‐based monomers and their all‐carbon backbone cannot be biodegraded, according to the intergovernmental Organization for Economic Co‐operation and Development (OECD) standards, leading to their accumulation in the environment.^[^ ^7^, ^8^, ^9^ ^]^ Much work is being conducted to create bio‐based polymers that are susceptible to biodegradation and have potential for application in PLFs.^[^ ^10^, ^11^ ^]^ However, there is limited understanding of how these polymers will biodegrade, mainly due to the limited methods currently available for studying polymer degradation in aqueous solutions which, lack accuracy and present an incomplete narrative.

**Figure 1:**
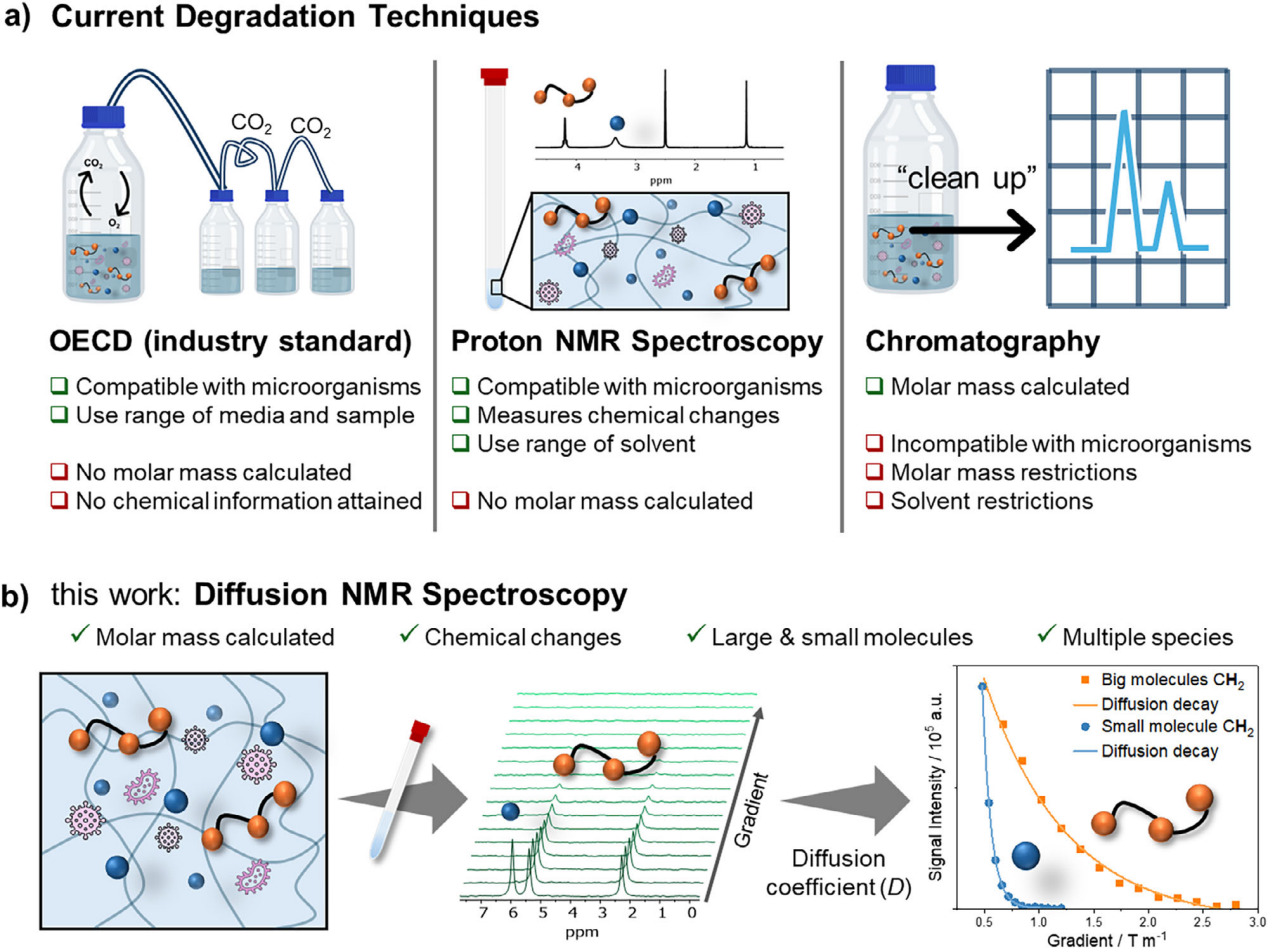
a) Scheme describing current degradation techniques to measure biodegradation of polymers including Organization for Economic Co‐operation and Development (OECD), proton NMR spectroscopy, and chromatography methods. b) Schematic of this work focused on how diffusion NMR spectroscopy is used to measure polymer biodegradation. The schematic displays two representative extreme diffusion decays of a big and small molecule.

The most commonly applied standardized biodegradation tests (i.e., OECD standards) use the mineralization of polymers to CO_2_ to infer the extent of degradation.^[^ ^12^ ^]^ These methods only report the result of the biotic metabolization of either polymer or their hydrolytic degradation products (Figure 1a). It is not possible to measure the degradation of the polymer directly, meaning that subtle differences in polymer structure or the impact of other components in the formulation cannot be identified. Several other analytical methods have been used to study the degradation of polymers in solution.^[^ ^13^ ^]^ Proton nuclear magnetic resonance (NMR) spectroscopy provides a method to directly measure chemical changes in the polymer structure, including degradation product formation, however, in the absence of functional end‐groups is not able to measure the molar mass of the polymer.^[^ ^14^, ^15^, ^16^ ^]^ This particularly inhibits the understanding of polymer biodegradation as a result of degradation products being consumed by the organisms.

Chromatography‐based techniques provide a well‐established relative method for measuring polymer molar mass, however, numerous sample incompatibilities complicate sample preparation, which is exacerbated by the presence of bacteria for biodegradation studies.^[^ ^17^, ^18^ ^]^ For example, while the accurate measurement of the molar mass of a polymer is possible using size‐exclusion chromatography (SEC) with appropriate detectors,^[^ ^19^ ^]^ simultaneous accurate measurement of the degradation products is limited, as a result of significant changes in hydrodynamic radii (*R*_H_) and overlapping solvent fronts.^[^ ^20^ ^]^ Recently, SEC techniques have been merged with matrix assisted laser desorption ionization‐time of flight (MALDI‐TOF) to improve the characterization of polymer mixtures.^[^ ^21^, ^22^ ^]^ However, following polymer degradation still proves challenging as polymers and resultant degradation products require contrasting matrices, laser power and cationizing agents as a consequence of their different polarity and solubility.^[^ ^23^ ^]^

To overcome these challenges, we hypothesized that diffusion NMR spectroscopy would enable accurate polymer degradation data to be obtained to fully understand polymer biodegradation with limited sample preparation (Figure 1b). Diffusion NMR spectroscopy has been previously shown to be suitable for the measurement of accurate molar masses for polymers as first applied by Johnson.^[^ ^24^ ^]^ Grubbs achieved accurate molar mass calculations of living polymerizations with this methodology since being extended to characterize the polymerization of l‐lactide, styrene and methyl methacrylate.^[^ ^25^, ^26^, ^27^, ^28^ ^]^ Recently, Junkers and coworkers reported a method to generate a universal calibration curve, comprised of polyethylene glycol (PEG) and polystyrene (PS) calibrants and accounting for the solvent viscosity.^[^ ^29^ ^]^ This solvent independent calibration curve has subsequently been used to determine the molar mass of poly(methyl methacrylate) (PMMA) and PEG copolymers,^[^ ^30^ ^]^ as well as to exploit the potential of low‐cost bench top NMR.^[^ ^31^, ^32^ ^]^ Further variation of a solvent independent calibration curve was also successfully demonstrated by Grabe and coworkers, who also measured the diffusion coefficient of both small and large molecules.^[^ ^33^, ^34^ ^]^ Additional works into parameter optimization have shown the importance of sample concentration, temperature, and pulse program.^[^ ^35^, ^36^ ^]^ Meanwhile, studies have been conducted on the quantification and characterization of post‐consumer plastics, which demonstrates diffusion NMR spectroscopy's potential to be used by environmental research facilities to assess polymer pollutants non‐destructively within the environment.^[^ ^37^, ^38^ ^]^ However, to date, diffusion NMR spectroscopy has not been used to follow and analyze polymer hydrolysis or biodegradation, despite its clear benefits.

Beyond these advances, the ability to calculate molar mass for each proton environment in the NMR spectrum, enabling multiple separate species, regardless of size, to be analyzed at a time, provides a significant potential advantage of diffusion NMR spectroscopy for polymer degradation analysis. Furthermore, thanks to simple sample preparation, diffusion NMR spectroscopy can be easily applied to polymer mixtures that mimic environmental samples, allowing the influence of different components on each other to be understood. To this end, herein we demonstrate that diffusion NMR spectroscopy provides a simple and reliable technique to fully characterize and understand the biodegradation of polymer mixtures. Using poly(5‐methyl‐5‐carboxyl‐1,3‐dioxan‐2‐one) (PMC) as a model water‐soluble, biodegradable polymer, we first demonstrate the comparability of SEC and diffusion NMR spectroscopy for the analysis of the hydrolytic degradation of PMC in a controlled buffer system. Subsequently, we report the biodegradation of PMC using diffusion NMR spectroscopy. The analysis of chemical and molar mass evolution of both large and small molecules gives rise to mechanistic insights that suggest that both hydrolysis and biodegradation mechanisms occur simultaneously. Finally, we demonstrate the ability to measure the degradation of a binary polymer mixture, in which the different rates of degradation can be clearly quantified.

## Results and Discussion

**Figure 2:**
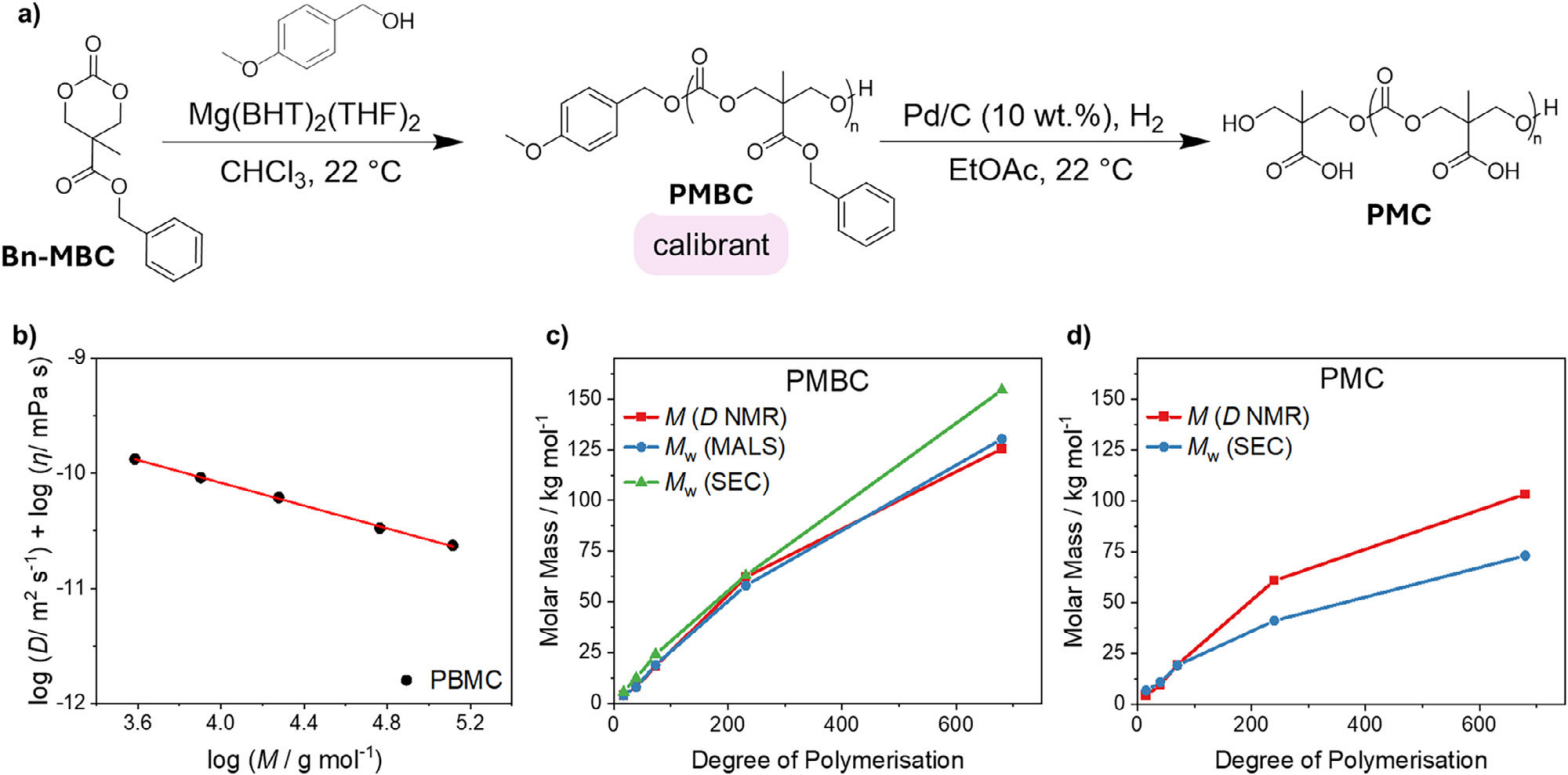
a) ROP of Bn‐MBC to obtain PMBC and its deprotection to PMC. b) Calibration curve of PMBC where the logarithm of diffusion coefficient and viscosity is plotted against the logarithm of molar mass. c) Molar masses of PMBC series plotted against the degree of polymerization (DP) comparing diffusion NMR spectroscopy, MALS, and SEC. d) Molar masses of PMC plotted against DP comparing diffusion NMR spectroscopy and weight‐average molar mass obtained from SEC.

Poly(5‐methyl‐5‐carboxyl‐1,3‐dioxan‐2‐one) (PMC) was selected as a model polymer on account of established synthetic procedures and known solubility in aqueous solutions.^[^ ^39^, ^40^ ^]^ The presence of hydrolysable carbonate bonds in the backbone was anticipated to provide a polymer that would be suitable for model degradation studies conducted in aqueous solutions. To this end, a six membered benzyl‐protected monomer, 5‐methyl‐5‐benzyloxycarbonyl‐1,3‐dioxan‐2‐one (Bn‐MBC), was synthesized and polymerized via ring opening polymerization (ROP) to form poly(5‐methyl‐5‐benzyloxycarbonyl‐1,3‐dioxan‐2‐one) (PMBC), according to literature protocols (Figures 2a and S1).^[^ ^41^ ^]^ Polymerizations of the hydrophobic monomer were conducted using 4‐methoxybenzyl alcohol as initiator, such that it would be removed upon benzyl group deprotection, and magnesium 2,6‐di‐tert‐butyl‐4‐methylphenoxide (Mg(BHT)_2_(THF)_2_) as the catalyst. PMBC polymers were subsequently deprotected by hydrogenation over Pd/C to afford PMC (Figures 2a and S2).

Polymers with various degrees of polymerization (DP) of 15, 40, 75, 240, and 680 were synthesized and DP was confirmed by ^1^H NMR spectroscopic analysis of the polymer with relative molar masses measured using SEC (Figures S3a and S4). The chromatograms displayed unimodal distributions with increasing dispersity at higher polymer DP, attributed to the higher probability of transesterification side reactions at longer chain lengths. Absolute molar mass measurements were also conducted for PMBC samples by SEC equipped with a multi‐angle light‐scattering (SEC‐MALS) detector in dimethyl formamide (DMF), to enable their use as calibrants for diffusion molar mass conversions (Figure S3b). Diffusion coefficients (*D*) of the PMBC and PMC polymers were measured using diffusion NMR spectroscopy, using bipolar gradient pulse pairs to minimize the ^1^H‐^1^H nuclear Overhauser effect (NOE) during the diffusion delay and reduce eddy currents.^[^ ^42^ ^]^ Values of *D* were calculated using the Stejskal‐Tanner equation^[^ ^43^ ^]^ (see page S7 in Supporting Information) and were found to decrease at higher DPs (Figures S5). The diffusion coefficients of the PMBC polymers were then plotted against the corresponding absolute molar masses of PMBC, obtained by SEC‐MALS, to create a solvent independent calibration curve following the protocol previously established by Junkers and co‐workers (Figure 2b and Equation S5, with further detail given on page S8 in Supporting Information).^[^ ^29^ ^]^ This method allows the use of a single calibration curve to analyze a polymer across a range of solvents. Furthermore, it enables the construction of a calibration curve capable of relating diffusion and molar mass in the absence of errors that would arise from the mismatch between the hydrodynamic radius (*R*_H_) of PMBC and standard calibrants that are used in conventional SEC.

To ensure diffusion NMR spectroscopy afforded accurate molar mass measurements, the molar masses of PMBC were then calculated using this calibration curve and compared to the values obtained by SEC and SEC‐MALS. Molar masses derived from diffusion NMR spectroscopy showed an agreement with SEC and SEC‐MALS analyses with the exception of DP 680, where SEC calibrated against polystyrene standards overestimated the weight‐average molar mass (*M*_w_) of the PMBC (Figure 2c). This reinforces common implicit errors that can arise within SEC measurements for samples containing different structures and *R*_H_ to their standards.^[^ ^44^, ^45^ ^]^ The *R*_H_ of PMBC did not change upon removal of the benzyl protecting group to afford PMC, as calculated with the Stokes–Einstein equation. Indeed, the *R*_H_ of the two polymers increased proportionally from DP 10 to 240, confirming that PMBC was a suitable calibrant to accurately measure PMC mass loss (Equation S2 and Figure S6). The same calibration curve, relating *D* to molar mass, that was previously used for PMBC, was later used to convert the *D* of the PMC series to molar mass (Figure 2b). At low DPs, *M*_w_ of the PMC measured by aqueous SEC (using PEG calibrants) and diffusion NMR spectroscopy was in good agreement (Figure 2d). However, at DP 240 and 680, there was a significant deviation, with diffusion NMR spectroscopy calibrated via a structurally analogous polymer, reporting a higher *M*_w_, suggesting that the mismatch of *R*_H_ in solution between the PEG calibrant and PMC underestimated the molar mass of the polymer (Figures 2d and S6).^[^ ^18^ ^]^

**Figure 3:**
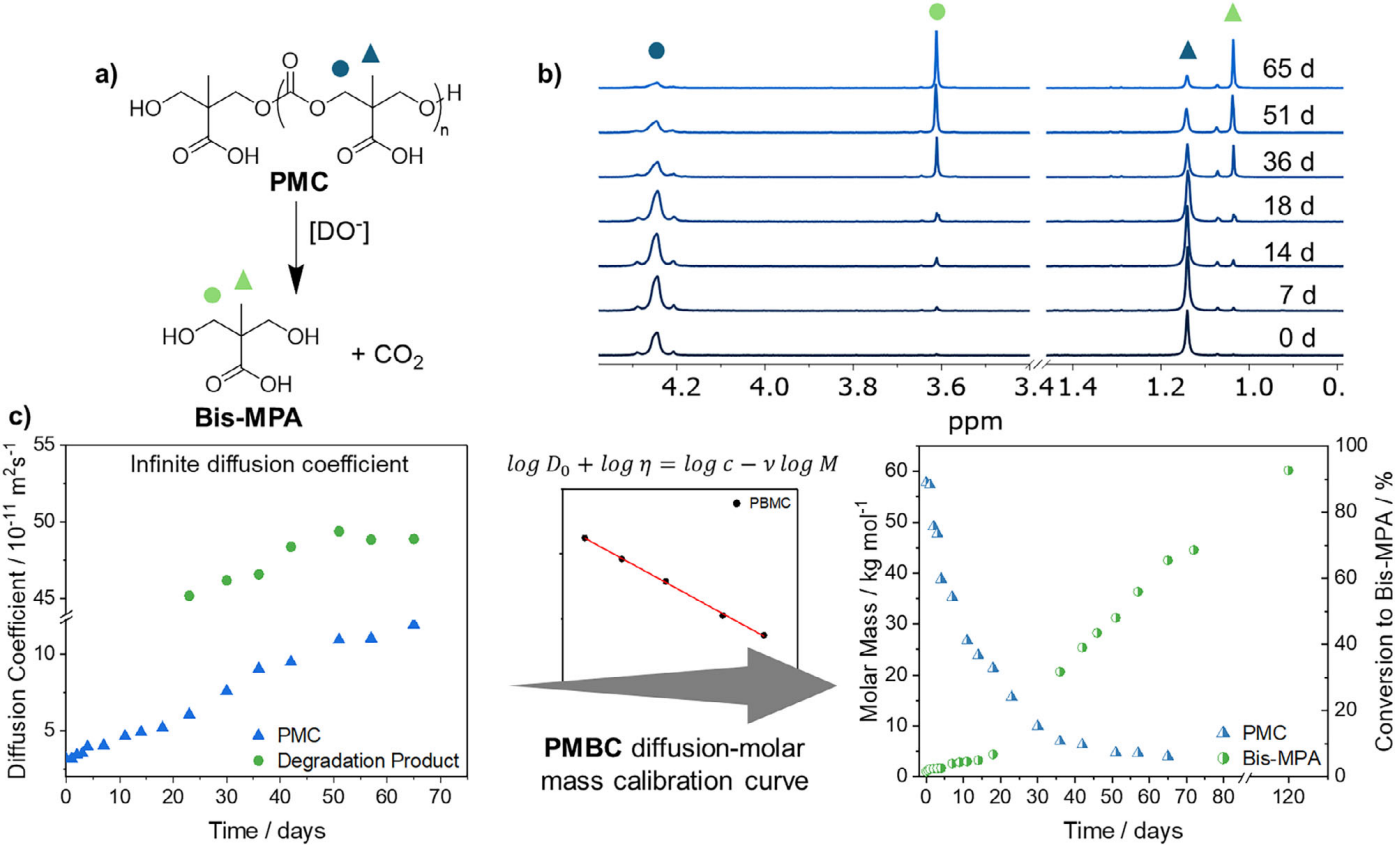
a) Schematic of PMC degradation. b) Stacked ^1^H NMR spectra (300 MHz, D_2_O) of PMC hydrolysis in sodium carbonate buffer (pH 9.1). c) Diffusion coefficients of PMC and bis‐MPA at infinite dilution over time being converted via the PMBC calibration curve to molar mass (left *y*‐axis) plotted against time and the conversion of PMC to bis‐MPA (right *y*‐axis).

Diffusion NMR spectroscopy was then employed to investigate the hydrolysis of PMC into a diol (2,2‐bis(hydroxymethyl)propionic acid (bis‐MPA)) and CO_2_ to demonstrate that the degradation of the polymer could be measured in a model system (Figure 3a). To this end, PMC with DP 240 was dissolved in a sodium carbonate buffer (pH 9.1) and incubated at 19 °C under stirring for 65 days. The buffer was prepared with D_2_O to increase the signal intensity and enable early characterization of degradation products. DP 240 was preferentially used as a consequence of its high starting molar mass and similar *R*_H_ of PMC and PMBC calibrants to ensure better agreement between the two analytical techniques from the start of the experiment (Figure S6).

The accuracy of the diffusion coefficient, and hence molar mass determination, is greatly impacted by numerous external factors. These include obstruction effects, which arise from the alcohol groups present in PMC and bis‐MPA undergoing a proton exchange with surrounding water molecules, and changes to solvent viscosity as a consequence of PMC's degradation into bis‐MPA.^[^ ^46^, ^47^ ^]^ The impact of viscosity and obstruction effects changing with PMC and bis‐MPA concentrations was confirmed with rheology and diffusion NMR spectrometry, respectively, using separate model solutions of both species with concentrations ranging from 1–0.0625 mg·mL^−1^ (Figures S7 and S8). Aliquots from subsequent degradation solutions were diluted to 1, 0.5, and 0.25 mg·mL^−1^ at each time point. The diffusion coefficients measured at each of these concentrations were then plotted and used to extrapolate back to infinite dilution (*D*_0_), thereby, reducing any impact from external contributions on the value of *D* used at each time point (Figure S9). The negative gradient observed across the different dilutions, for each time point, demonstrates the impact of these external factors on the *D*. The value *D*_0_ was then used in subsequent molar mass calculations. Furthermore, NMR experimental parameters were optimized and continuously adapted to attain maximum signal attenuation (see Section 2.4 in Supporting Information). Additionally, variability between samples was minimal, with a calculated 1.5% standard error as a percentage of the mean. Therefore, due to the timescale of the data collection no repeats were conducted (Figure S10).

Initial analysis of the ^1^H NMR spectra measured during the PMC degradation showed a decrease in the intensity of PMC peaks (*δ* = 4.25 and 1.05 ppm) over time. New peaks at *δ* = 3.60 and 1.25 ppm were detected and confirmed the formation of bis‐MPA as the degradation product (Figures 3b and S11). The clear individual proton environments of the two species facilitate the analysis of the diffusion coefficient meaning both compounds can be examined simultaneously. *D* for the degradation product was calculated from 23 days onward and was constant at around 4 × 10^−10^ m^2^·s^−1^, in reasonable agreement with the diffusion coefficient of commercial bis‐MPA (Figure S12). We note that the slight variance in measured *D* for bis‐MPA in the degradation solution, compared to that measured independently results from optimization of the diffusion parameters for the PMC, rather than for bis‐MPA. These parameters can readily be optimized for a different species of interest, such as the degradation product, as diffusion NMR spectroscopy is not inherently limited by the molar mass of the polymer, but the maximum gradient accessible by the NMR probe. The conversion of *D*_0_ to molar mass, using the pre‐established PMBC calibration curve, found that the product had a molar mass of 240 g·mol^−1^. Liquid chromatography‐mass spectrometry (LC‐MS) applied as a confirmatory technique, showed the presence of bis‐MPA (*m/z* 133 g·mol^−1^) and a dimer (*m/z* 280 g·mol^−1^) formed as a result of strong electrostatic attraction between the sodium and carboxylic acid groups (Figure S13). After extrapolating to infinite dilution, *D*_0_ of PMC showed a linear increase from 3 ×10^−11^ to 1 × 10^−10^ m^2^·s^−1^ over 65 days (Figure 3c). The conversion to molar mass using the same pre‐established calibration curve exhibited a 93% mass loss of PMC over 65 days from 58 to 4 kg·mol^−1^ (Figure 3c and Table S5). Comparing these molar mass measurements to SEC (using PEG calibrants) revealed a smaller *M*_w_ reduction of 82%, with molar masses plateauing at 8 kg·mol^−1^ (Table S5 and Figure S14). This difference in molar mass was probably a result of SEC utilizing a PEG calibration curve, which has a different *R*_H_ than PMC (Figure S6). Converting the data into the rate of PMC hydrolysis revealed significant differences; SEC showed second order rate kinetics, with a rate constant of 9 × 10^−3^ M^−1^·s^−1^, while diffusion NMR spectroscopy was better fitted by two first order rate constants of 0.05424 and 0.012 s^−1^ after 50 days (Figures S15 and S16). This change in rate was not visible in the SEC data and was linked to the PMC oligomers becoming more soluble in the D_2_O buffer than its high molar mass PMC chains, ultimately increasing the rate of carbonate hydrolysis. Despite the benefits of diffusion NMR spectroscopy in offering mechanistic insights, as well as molar mass of multiple species, no comment can be made on polymer dispersity using this technique alone, although other studies have attempted to estimate dispersity values from diffusion NMR spectroscopy.^[^ ^48^ ^]^

To confirm that the observed differences in rate arose as a result of the calibrant used on the SEC, diffusion NMR spectroscopy was employed to create a calibration curve for conventional SEC using the PMC molar masses derived from the previous diffusion measurements. This resulted in improved molar mass measurements that aligned with diffusion NMR spectroscopy data, especially at low molar masses (Figure 3c and Table S6). Crucially, after application of the PMC‐derived calibration curve, both techniques led to the observation of first order rate kinetics (Figure S17). This means all SEC instruments can be tailored with individual calibration curves to correct for *R*_H_ inconsistencies in SEC measurements.

The biodegradation of PMC with *Escherichia coli* (*E. coli*) was next analyzed using diffusion NMR spectroscopy. To prove PMC would not inhibit the growth of *E. coli*, bacterial plates comprised of Luria–Bertani (LB) agar were made up with an optimal polymer concentration for bacterial growth identified at 2 mg·mL^−1^ (Figure S18). Notably, colony growth was slightly inhibited by increasing PMC concentration, likely as a consequence of colonies fusing together; 10 mg·mL^−1^ PMC demonstrated the largest colony size (Figure S20b). Colony growth for bacteria exposed to the degradation product, bis‐MPA, was similar to the control experiment performed in the presence of super optimal broth with catabolite repression (SOC) media (Figure S20a). Furthermore, adding PMC to the growth media enabled more bacterial growth than when other polymers such as polyvinyl alcohol (PVA) or PEG samples were present (Figures S19 and S20d). To demonstrate bacterial consumption of the polymer, agarose plates, with only the polymer as a food source, were prepared. Growth of bacterial colonies under these conditions confirmed bacterial uptake of PMC at 2 mg·mL^−1^ (Figure S21). However, increasing the polymer concentration up to 10 mg·mL^−1^ inhibited bacterial growth, likely as a result of the increased acidity from the carboxylic acid groups. As anticipated, bacteria that were exposed to bis‐MPA exhibited the most growth as a result of small molecules being easier to uptake into the bacterial cell. Interestingly, colonies that had been exposed to PMC / bis‐MPA had a larger colony size than those incubated with SOC media and turned a pink color, which suggests that the consumed metabolites affected the bacteria's pH (Figures S21, S22, and S24a). In contrast, the positive control experiment conducted with PVA, exhibited the largest colony growth with few colonies changing color, highlighting the higher metabolic rate of *E. coli* toward PVA than PMC (Figures S22 – S24).

**Figure 4:**
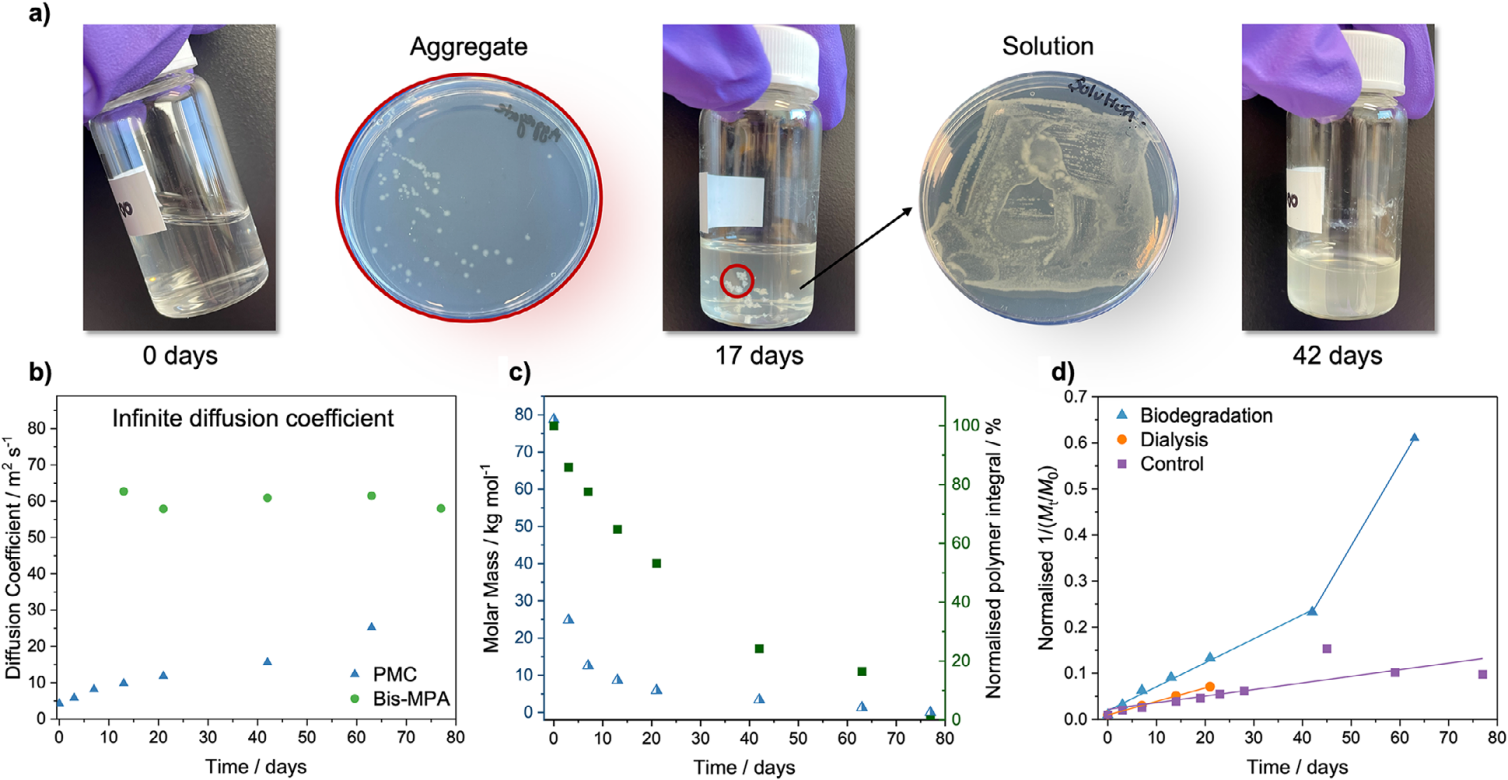
a) Images of PMC degradation for 0, 17, and 42 days as well as bacterial plates demonstrating bacterial growth from aggregates and solution. b) Diffusion coefficient at infinite dilution of PMC and bis‐MPA over biodegradation period. c) Calculated molar mass of PMC (left *y*‐axis) and the normalized polymer integral (right *y*‐axis) plotted against time. d) Normalized second order rate kinetics, (1/(*M*_t_/*M*_0_)) plotted against time for biodegradation, dialysis, and control experiments, all incubated at 37 °C at 180 rpm.

In order to monitor the polymer biodegradation, PMC was dissolved in 0.05 M phosphate buffered saline solution (PBS) prepared with H_2_O at 2 mg·mL^−1^, to ensure complete dissolution of the polymer, and incubated at 37 °C with the addition of *E. coli*. Over 11 weeks the solution transitioned from clear to cloudy, with aggregates forming after 7 days (Figures 4a and S25). Extracts of the solution and aggregates grew on nutrient rich agar, which confirmed the presence of live colonies (Figure 4a). Diffusion NMR spectroscopy could then be easily used to quantify and understand the biodegradation of PMC. ^1^H NMR spectra enabled bis‐MPA to be identified as the main degradation product, which could have been produced as a result of either hydrolysis and/or biodegradation of PMC (Figure S26). A new proton environment at *δ* = 1.9 ppm was also seen in low concentration, only after 3 days, indicating the formation of a byproduct, which was subsequently consumed by the *E.coli* during the initial incubation period, when molar mass loss is at its fastest. This byproduct was not seen at any other time point.

The change in molar mass of the polymer and degradation product can be calculated from the diffusion coefficient at infinite dilution following the method established above (Figure S27). Again, a linear increase in the diffusion coefficient of PMC at infinite dilution was observed (Figure 4b). The calculated molar mass for bis‐MPA increased from 204 g·mol^−1^ to an average of 220 g·mol^−1^ over the degradation period. This indicates the formation of the dimeric species observed during PMC hydrolysis (Figure S28). The polymer exhibited an exponential decay in molar mass during biodegradation with 84% molar mass loss in the first week followed by a linear, but low rate of degradation, from 21 days onward, until degradation was complete at 11 weeks (determined by diffusion NMR spectroscopy, Figure 4c). The comparison of the molar mass of PMC with the integrals of the polymer taken from the NMR spectra, showed that the significant initial molar mass loss was not reflected in the consumption of the polymer, where only 22% of the original polymer integral had been lost in the first week (Figure 4c). From 21 to 77 days, the NMR spectra demonstrated that the polymer was being preferentially consumed, where a 53% reduction in polymer concentration was exhibited in the final 8 weeks of the study, compared to a 7.5% molar mass change (Figure 4c). Therefore, whilst the chains are decreasing in length, the number of polymer chains is not decreasing as rapidly, which suggests that the large chains may initially be degrading by hydrolysis with subsequent biodegradation only possible for the polymer chains of lower molar mass.

To confirm that biodegradation of the polymer was taking place, PMC was dissolved in 0.05 M PBS prepared in H_2_O with ampicillin sodium salt, to prevent any bacterial contamination. Under these conditions, PMC degraded more slowly, with a reduction in molar mass of only 60% in the first week. Notably, after 42 days, in the absence of biodegradation pathways, the PMC integral decreased more slowly than when bacteria were present (Figures S29 and S30). Biodegradation studies were also conducted within a dialysis bag to ensure the removal of the buffer and degradation product. Diffusion NMR spectroscopy was used to measure the molar mass over 21 days after which the polymer had degraded to a size that it could pass through the dialysis bag and could no longer be analyzed (Figure S31). An 86% mass loss was observed over 21 days (Figure S32c), with the molar mass of PMC decreasing faster than in the control experiment with no bacteria, albeit slower than the original mixture of bacteria and PBS. Under all three conditions, polymer degradation displayed second order kinetics (Figure 4d), most likely a consequence of the increased solubility of PMC in H_2_O, compared to D_2_O. Interestingly, the degradation of PMC in the presence of bacteria was better fitted by two second order fittings of 0.0052 and 0.0 1803 M^−1^·s^−1^, demonstrating the increased consumption of PMC once a smaller chain length had been reached (Figure S33a). These results show that the rate of biodegradation of PMC is increased in the presence of bacteria (Figure S34), with higher total polymer consumption observed over 80 days. These findings suggest that different degradation mechanisms occur when *E. coli* is present which lead to an increase in degradation rate for the high molar mass polymers, and that biodegradation is the major degradation pathway at low molar masses of around 7 kg·mol^−1^ and below. This mechanistic insight was only attainable through the combination of molar mass and NMR integrals obtained through diffusion NMR spectroscopy.

**Figure 5:**
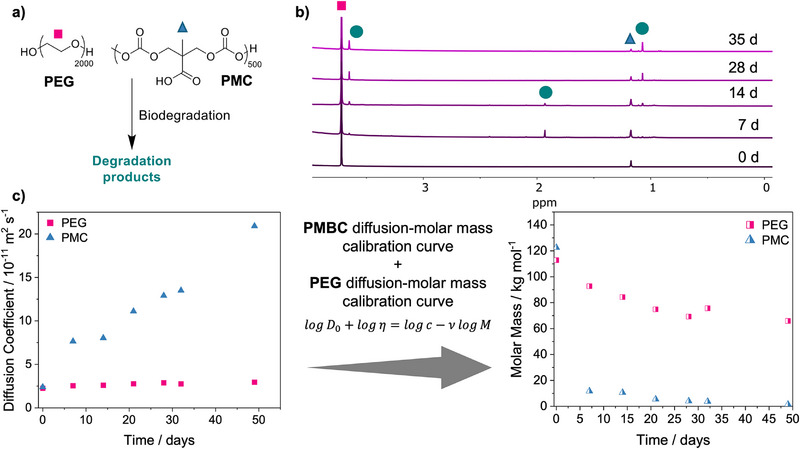
a) Scheme of biodegradation of PMC and PEG. b) Stacked ^1^H NMR spectra (300 MHz, H_2_O) over time for biodegradation of PEG and PMC. c) Diffusion coefficients of PMC and PEG at infinite dilution over time being converted via PMBC calibration curve for PMC and PEG calibration curve for PEG polymers to molar mass over time.

Finally, we sought to demonstrate that the ability of diffusion NMR spectroscopy to measure the diffusion coefficient for each chemical environment visible in the spectra, such that they could be leveraged to simultaneously analyze the degradation of multiple species in more complex mixtures. To this end, we studied the biodegradation of a binary polymer mixture of PMC and PEG. To demonstrate this, *E. coli* was added to a mixture of PMC and PEG, with similar starting molar masses, dissolved in 0.05 M PBS (DI H_2_O). The degradation of each polymer was monitored through diffusion NMR spectroscopy so that the molar mass and NMR spectra could be analyzed over time (Figures 5 and S35). The expected conversion of PMC to bis‐MPA was observed, where PMC demonstrated a 90% molar mass loss within one week. Interestingly, this was a much faster rate of PMC degradation than had been observed previously in the absence of PEG, but agrees with previous studies in the literature, where PEG was found to accelerate the hydrolysis of polyphosphazene polyelectrolytes.^[^ ^49^ ^]^ PMC also no longer followed first or second order rate kinetics, further indicating that the addition of PEG actively impacts the degradation rate. However, it must be noted that at the final measurement point at 49 days, the PMC signal was very weak, therefore is subject to higher error (Figures S36 and S37). Over this period, the *D*_0_ of PEG increased by 7 × 10^−12^ m^2^·s^−1^, consistent with low levels of chain scission but no loss of mass, confirmed by ^1^H NMR spectroscopy signal integrals (Figure S38). Notably the PEG chain scission occurs with and without either bacteria or PMC and could result from the high stirring rate used (Figures S39 and S40).^[^ ^50^, ^51^ ^]^ Moreover, the NMR spectra exhibited a new degradation product at 7 and 14 days only, with a new peak arising at *δ* = 1.8 ppm, which suggests that a new proton environment is being formed and subsequently consumed by the *E.coli* (Figure 5b). This species was also formed during the biodegradation of PMC in the absence of PEG, but was limited to lower concentrations, further indicating that the presence of PEG alters the biodegradation of PMC. Hence, diffusion NMR spectroscopy was used to follow the degradation of two polymer species with the same starting molar mass, which is not possible with conventional techniques as a consequence of overlapping mass distributions. Therefore, the use of diffusion NMR spectroscopy opens the possibility for biodegradation studies in more complex environments, such as wastewater, where polymer mixtures are present.

## Conclusion

In this study, we have demonstrated how diffusion NMR spectroscopy can be applied to monitor water‐soluble polymer biodegradation. The whole polymer biodegradation process can now be followed, characterized and understood using one technique, with minimal sample preparation, circumventing the previously stated limitations associated with biodegradation studies. We have shown how accurate molar masses can be calculated for any species within a mixture, regardless of size, meaning that environmental samples containing a variety of polymers with the same starting molar mass can be analyzed. This allows each component of complex formulations to be analyzed, hence furthering understanding of their effect on polymer biodegradation. Through diffusion NMR spectroscopy it is possible to further our understanding of the biodegradation mechanism of polymers included in formulations through the direct analysis of environmental samples. Therefore, ensuring the extent of PLF degradation in wastewater treatment systems and the environment can be conducted to understand their inevitable impact on our ecosystems.

## Supporting Information

The authors have cited additional references within the Supporting Information.^[^ ^43^, ^44^, ^45^, ^46^, ^47^, ^48^, ^49^, ^50^, ^51^, ^52^, ^53^, ^54^, ^55^, ^56^, ^57^ ^]^

## Conflict of Interests

The authors declare no conflict of interest.

## Supporting information



Supporting Information

## Data Availability

The data that support the findings of this study are available from the corresponding author upon reasonable request.
