# Supplementary material for: Quantifying the Biodegradation of Water‐Soluble Polymer Mixtures with Diffusion NMR Spectroscopy
Source: Angew Chem Int Ed Engl. 2025 Sep 26;64(47):e202514235. doi: 10.1002/anie.202514235 (PMC12624306; doi:10.1002/anie.202514235)
Supplement: Supplementary file 1 — Supporting Information [file ANIE-64-e202514235-s001.docx]

Supplementary Information for

**Quantifying the biodegradation of water-soluble polymer mixtures with diffusion NMR spectroscopy**

Louisa T. Brenninkmeijer,^a^ Jacob L. Golding,^a^ Dr Arianna Brandolese,^a^ Prof. Melanie M. Britton,^a^* Prof. Andrew P. Dove^a^*

a. School of Chemistry, University of Birmingham, Edgbaston, Birmingham, B15 2TT, U.K.

*Corresponding Authors: [a.dove@bham.ac.uk](mailto:a.dove@bham.ac.uk), [m.m.britton@bham.ac.uk](mailto:m.m.britton@bham.ac.uk)

**Contents**

[**List of Figures** 3](#_Toc201747012)

[**1.** **General Information** 7](#_Toc201747013)

[**1.1** **Diffusion NMR Spectroscopy Theory** 7](#_Toc201747014)

[**1.1.1 Diffusion – Molar Mass Calibration Curve** 8](#_Toc201747015)

[**2. Experimental Procedures** 9](#_Toc201747016)

[**2.1 Reagents.** 9](#_Toc201747017)

[**2.2 Synthetic Procedures.** 9](#_Toc201747018)

[**2.3 Characterization Techniques.** 11](#_Toc201747019)

[**2.4 Diffusion NMR spectroscopy parameters.** 13](#_Toc201747020)

[**3.** **Appendix** 17](#_Toc201747021)

[**4.** **Diffusion NMR spectroscopy signal decays** 43](#_Toc201747022)

[**5.** **References** 65](#_Toc201747023)

# **List of Figures**

[**Figure S1**. ^1^H NMR (CDCl_3_, 400 MHz, 298 K) and ^13^C NMR (100.57 MHz) of protected PMBC polymer, DP 240 1](#_Toc197714822)7

[**Figure S2** ^1^H NMR (DMSO-d_6,_ 400 MHz, 298 K) and ^13^C NMR (100.57 MHz) of deprotected PMC, DP 240 1](#_Toc197714823)8

[**Figure S3 (a)** Size exclusion chromatograms of protected PMBC polymers (CHCl_3_ with 0.5% NEt_3_, PS standards, RI detector) **(b)** Size exclusion chromatograms of PMBC polymers measured in DMF (containing 5 mM NH_4_BF_4_) coupled with a multi angle light scattering (MALS) detector. 1](#_Toc197714824)9

[**Figure S4** Size exclusion chromatograms of deprotected PMC polymers (80:20 H_2_O:MeOH, 0.1 M NaNO_3_, PEG standards, RI detector). 1](#_Toc197714825)9

[**Figure S5 (a)** Diffusion coefficients (D) for Bn-MBC monomer and protected PMBC (CH_2_ environment) in CDCl_3_ plotted against the degree of polymerisation (DP). **(b)** D for Bn-MBC and deprotected PMC (CH_2_ environment) in DMSO-d_6_ plotted against DP. 20](#_Toc197714826)

[**Figure S6** Hydrodynamic radius of PMBC and PMC calculated from diffusion coefficients measured above via the Stokes-Einstein equation. 20](#_Toc197714827)

[**Figure S7** Viscosity plotted against concentration of **(a)** bis-MPA and **(b)** PMC measures with a rheometer as described above. 21](#_Toc197714828)

[**Figure S8** Diffusion coefficient plotted against concentration of **(a)** bis-MPA and **(b)** PMC (300 MHz, 298 K, G_max_ = 2.8 T m–1, G_min_ = 0.14 T m–1, δ=2 ms, Δ= 100 ms, and repetition time = 1 s). 21](#_Toc197714829)

[**Figure S9** Diffusion coefficient plotted against concentration of PMC polymers over time to extrapolate to infinite dilution over the 665-daydegradation period at 19 °C, NaBS pH 9.1 made up with D_2_O. 22](#_Toc197714830)

**Figure S10** D_IFFUSION COEFFICIENT PLOTTED FOR 4 PMC POLYMER SAMPLES (DP 240, H2O, 300 MHz, 298 K, GMAX = 2.5 T·M_^–1^_, GMIN = 0.75 T·M_^–1^_, δ= 2 MS, Δ= 100 MS, AND REPETITION TIME = 1 s) INDICATING THE MINIMUM VARIABILITY OF THE DIFFUSION NMR MEASUREMENT_……………..……22

[**Figure S11** Stacked ^1^H NMR spectra (300 MHz, D_2_O. 298 K) of PMC hydrolysis in NaBS (pH 9.1) with the identified peaks of PMC and bis-MPA. 23](#_Toc197714831)

[**Figure S12** Comparison of the diffusion coefficient of the degradation product and commercial bis-MPA **(a)** Diffusion coefficients (300 MHz, 298 K, G_max_ = 2.8 T_·_m^–1^, G_min_ = 0.14 T·m–1, δ=2 ms, Δ= 100 ms, and repetition time = 1 s) **(b)** ^1^H NMR spectra (300 MHz, D_2_O, 298 K). 23](#_Toc197714832)

[**Figure S13** LC-MS (ESI –TOF) (negative) of PMC degradation sample in pH 9.1 (D_2_O), 24 °C after 120 days. Mobile phase was 70:30 to 40:60% H_2_O:ACN, with gradient at 10 min and flow rate of mobile phase was 0.2 mL·min^–1^. 24](#_Toc197714833)

[**Figure S14** Size exclusion chromatograms (80:20 H_2_O:MeOH, 0.1 M NaNO_3_, PEG calibrants) for hydrolysis of PMC in pH 9.1, 19 °C monitored for 65 days. 25](#_Toc197714834)

[**Figure S15** Inverse of the normalised molar mass of PMC vs time of PMC hydrolysis (19 °C, pH 9.1 D_2_O) comparing SEC and diffusion NMR spectroscopy showing the second order rate constant calculated via M_w_. 26](#_Toc197714835)

[**Figure S16** **(a)** Natural log of the normalised molar mass of PMC vs time of PMC hydrolysis (19 °C, pH 9.1 D_2_O) comparing SEC and diffusion NMR spectroscopy showing the first order rate constant calculated via diffusion NMR spectroscopy **(b)** Natural log of the normalised molar mass of PMC vs time of PMC hydrolysis in the same conditions from diffusion NMR spectroscopy showing the two first order rate constants calculated via M_w_. 26](#_Toc197714836)

[**Figure S17** Natural log of the normalised molar mass of PMC over the 65-day degradation period (19 °C, pH 9.1 D_2_O). 27](#_Toc197714837)

[**Figure S18** E. coli growth on nutrient rich LB agar for control Bia-MPA and PMC at concentrations of 2, 4, 10 mg mL^–1^, incubated at 37 °C for 2 days done in triplicates. 28](#_Toc197714838)

[**Figure S19** E. coli growth on nutrient rich LB agar, incubated at 37 °C for 2 days for control, PVA and PEG at 2 mg·mL^–1^ performed in triplicates. 28](#_Toc197714839)

[**Figure S20** Comparison of bacterial growth comparing **(a)** number of colonies **(b)** size of colonies **(c)** type pf polymer comparing number of colonies grown and **(d)** size of colonies. All plates were stored at 37 °C and performed in triplicates. 29](#_Toc197714840)

[**Figure S21** E. coli growth on pure agarose plates with control Bia-MPA and PMC at concentrations of 2, 4, 10 mg·mL^–1^ as food source, incubated at 37 °C for 8 days performed in triplicates. 30](#_Toc197714841)

[**Figure S22** Representative pure agarose plates of bis-MPA, PMC and PVA (2 mg·mL^–1^) after 7 days stored at 37 °C. 31](#_Toc197714842)

[**Figure S23** E. coli growth on pure agarose plates with PVA (2 mg·mL^–1^) as food source incubated at 37 °C for 8 days done in triplicates. 32](#_Toc197714843)

[**Figure S24** Comparison of E. coli growth on pure agarose plates (37 °C) comparing **(a)** PMC concentrations and bis-MPA and **(b)** PMC and PVA polymers. Experiments conducted in triplicates. 32](#_Toc197714844)

[**Figure S25** Pictures of PMC (2 mg·mL^–1^) with E. coli incubated at 37 °C shaken at 180 rpm. 33](#_Toc197714845)

[**Figure S26** ^1^H NMR spectra (300 MHz, H_2_O, 298 K) of PMC biodegradation (E. coli, 0.05 M PBS (DI H_2_O), 37 °C, 180 rpm) sample over 77 days. 34](#_Toc197714846)

[**Figure S27** Diffusion coefficients of PMC biodegradation (E. coli, 0.05 M PBS (DI H_2_O), 37 °C, 180 rpm) over the degradation period for concentrations 2, 1 and 0.5 mg·mL^–1^ followed by extrapolation to infinite dilution, which was then used for the subsequent calculations. 34](#_Toc197714847)

[**Figure S28** Molar mass of bis-MPA (using diffusion NMR spectroscopy) over the biodegradation period (E. coli, 0.05 M PBS (DI H_2_O), 37 °C, 180 rpm). 35](#_Toc197714848)

[**Figure S29** **(a)** Size exclusion chromatograms of PMC (80:20 H_2_O:MeOH, 0.1 M NaNO_3_, PMC calibrants) without bacteria present (0.05 M PBS DI H_2_O, 37 °C, 180 rpm, 0.083 equiv. ampicillin sodium salt) **(b)** mass loss (black line) and NMR integrals (purple dots) of PMC signal plotted against time for PMC biodegradation in the same conditions. **(c)** ^1^H NMR spectra (300 MHz, H_2_O, 298 K) of PMC degradation without bacteria present over 77 days. Peaks at δ= 1.06 and 3.64 ppm correspond to bis-MPA, peaks at δ= 1.16 and 4.21 correspond to PMC and peaks between δ= 1.40–1.56 ppm correspond to the ampicillin sodium salt. 36](#_Toc197714849)

[**Figure S30** **(a)** Normalised molar mass plotted against time of PMC in the presence of E. coli (biodegradation) and without (control) containing 0.083 equiv. ampicillin sodium salt in 0.05 M PBS (DI H_2_O) at 37 °C, 180 rpm. **(b)** Normalised absolute integral of PMC in presence of E. coli (biodegradation with E. coli) and without (named control) containing 0.083 equiv. ampicillin sodium salt in 0.05 M PBS (DI H_2_O) at 37 °C, 180 rpm. 37](#_Toc197714850)

[**Figure S31** ^1^H NMR spectra (300 MHz, H_2_O, 298 K) over 28 days for PMC biodegradation (2 mg·mL^–1^, E. coli, 0.05 M PBS (DI H_2_O)) in a dialysis bag at 37 °C, 180 rpm. 37](#_Toc197714851)

[**Figure S32 (a)** Diffusion coefficients of PMC within the dialysis bag (E. coli, 0.05 M PBS (DI H_2_O), 37 °C, 180 rpm) over the 22 day degradation period at concentrations of 2, 1 and 0.5 mg·mL^–1^ followed by extrapolation to infinite dilution to afford the corrected D. **(b)** The corrected D at infinite dilution, which was then used for the subsequent calculations. **(c)** Final molar mass results for PMC biodegradation with continuous removal of degradation products under same conditions. 38](#_Toc197714852)

[**Figure S33** **(a)** Second order rate kinetics of PMC biodegradation (E. coli, 0.05 M PBS (DI H_2_O), 37 °C, 180 rpm) **(b)** First order fitting of PMC biodegradation (E. coli, 0.05 M PBS (DI H_2_O), 37 °C, 180 rpm). 39](#_Toc197714853)

[**Figure S34** Comparison of rate constants from PMC degradation with and without bacteria (0.05 M PBS (DI H_2_O), 37 °C, 180 rpm). 39](#_Toc197714854)

[**Figure S35** Diffusion coefficients of **(a)** PMC and **(b)** PEG over the degradation period (E. coli, 0.05 M PBS (DI H_2_O), 37 °C, 180 rpm) at concentrations of 2, 1 and 0.5 mg·mL^–1^ followed by extrapolation to infinite dilution, which was then used for the subsequent calculations. 40](#_Toc197714855)

[**Figure S36 (a)** First order rate fitting of PMC in the presence of E. coli and PEG (E. coli, 0.05 M PBS (DI H_2_O), 37 °C, 180 rpm) **(b)** Second order rate fitting of the same condition.](#_Toc197714856) 40

[**Figure S37** Normalised second order plotted against time for PMC and PEG (E. coli, 0.05 M PBS (DI H_2_O), 37 °C, 180 rpm). 41](#_Toc197714857)

[**Figure S38** Absolute peak intensity of PEG plotted against time during biodegradation (E. coli, 0.05 M PBS (DI H_2_O), 37 °C, 180 rpm). 41](#_Toc197714858)

[**Figure S39 (a)** Size exclusion chromatogram of PEG during control experiment (no bacteria) (0.05 M PBS (DI H_2_O), 0.083 equiv. ampicillin sodium salt, 37 °C, 180 rpm) **(b)** Molar mass of PEG during control (no bacteria). 42](#_Toc197714859)

**Figure S40** C_OMPARISON OF MOLAR MASS OF PEG FROM BIODEGRADATION (WITH E. COLI, 0.05 M PBS (DI H2O), 37 °C AND 180 RPM) AND CONTROL EXPERIMENTS 0.083 EQUIV. AMPICILLIN SODIUM SALT, 0.05 M PBS (DI H2O), 37 °C AND 180 RPM), BOTH INCUBATED AT 37 °C AT 180 RPM_………..…43

# **General Information**

- 1. **Diffusion NMR Spectroscopy Theory**

Pulsed field gradient nuclear magnetic resonance (PGF-NMR) spectroscopy has been widely used to measure the self-diffusion coefficient of molecules. Free induction decays (FID) are collected at increasing gradient strength, from which the signal attenuation can be measured. Diffusion coefficients can then be obtained for each proton environment through the Stejskal-Tanner (S-T) equation (**Eq S1**).^[1]^ The S-T equation is commonly used for a Gaussian decay function, in which *I* and *I_0_* are the signal intensities in the pulse sequence with and without a gradient, *G*, respectively. *D* is the diffusion coefficient, γ is the gyromagnetic radius, *δ* is the gradient duration of the PFG signal and Δ is the observation time.

$I=I_{0}exp[-D\gamma^{2}\delta^{2}G^{2}(\Delta-\frac{\delta}{3})]$ (**Eq S1**)

Once the *D* is calculated it can be related to the hydrodynamic radius (*R_H_*) through the Stokes-Einstein equations where *k* is the Boltzmann constant, *T* is the temperature and $\eta$ is the viscosity of the solution (**Eq S2**).^[2]^

$D=\frac{kT}{6\pi\eta R_{H}}$ (**Eq S2**)

This can be extended to relate molar mass and *D* in a monodisperse polymer sample, where *A* and *α* are constants derived from the polymer and account for the space it fills in solution (**Eq S3**).^[3]^

$D=AM^{\alpha}$ (**Eq S3**)

Grubbs and co-workers further extended this relationship to afford a linear relationship between *D* and *M* (**Eq S4**).^[4]^ This equation utilises the Avogadro constant (*N*_A_) and density of the liquid ($\rho$) to account for the shape of the compound. This model assumes there were negligible polymer viscosity effects, with measurements taken from highly dilute sample conditions of 1 mg mL^–1^.

$\log D=-\frac{1}{3}\log M+\frac{1}{3}\log\rho-\log\eta-\frac{1}{3}\log\frac{162\pi^{2}}{k^{3}T^{3}N_{A}}$ (**Eq S4**)

Finally, Junkers and co-authors developed a solvent independent calibration curve by considering the solvent viscosity at a given temperature.^[5]^ This leads to the inclusion of proportionality factors c and ν taken from calibration standards fitted to this equation (**Eq 5**). For this, it was assumed that there were negligible solvent effects on a polymer’s *R*_H_, allowing for this comparison between solvents.

$\log D+\log\eta=\log c-\nu\log M$ (**Eq S5**)

In order to use the PMBC calibration curve measured in chloroform, we decided to use the Junkers model to attain the molar mass from the diffusion coefficient for subsequent experiments.

Viscosity of chloroform was taken from the literature used without changes.^[5]^ Viscosity of DMSO was extrapolated to 20 °C using data from the literature.^[6]^ Viscosity of the sodium buffer (NaBS) was measured using the rheometer using the method described below.

| Solvent | Viscosity at 20 °C / mPa·s |
| --- | --- |
| Chloroform | 0.563 |
| DMSO | 2.1734 |
| NaBS, pH 9.1 | 1.095 |

## **Diffusion – Molar Mass Calibration Curve**

To create the diffusion molar mass calibration curve, diffusion coefficients (*D*) of the PMBC polymers were measured *via* diffusion NMR spectroscopy. Polymers were dissolved in deuterated chloroform at 1 mg·mL^–1^ to minimise polymer-polymer interactions. *D* were calculated from the Stejskal-Tanner equation and were found to decrease at higher DPs and ranged from 2 × 10⁻^10^ to 4 × 10⁻^11^ m^2^·s^⁻1^ (**Eq S1, Figure S5**). Diffusion decays were fitted with single exponentials, except for DP 240 and 680, in which case biexponential fits were required to result in two components, with different *D* and magnitude, to be calculated (see section 4). The slower components at 3.7 × 10⁻^11^ and 5.7 × 10⁻^11^ m^2^·s^⁻1^, for DP 680 and 240 respectively, corresponded to the polymer and were always in higher proportionality (> 88%). Meanwhile, the faster component at 2 × 10⁻^10^ m^2^·s^⁻1^ could be related to small oligomers that were not completely removed from the polymer after precipitation. The former was not related to PMBC and was removed for the subsequent calibration curve.

Following this, *D* of PMC standards were measured in DMSO-d_6_ at 295 K. As previously observed for PMBC, *D* decreased with increasing DP, from 4 × 10⁻^10^ to 8 × 10⁻^12^ m^2^·s^⁻1^ (**Figure S5b**). The large variation in *D* between PMBC and PMC was attributed to the difference in solvent viscosity, as polymers were tested in CDCl_3_ and DMSO-d_6_, respectively. Biexponential fits were required for all samples, with the slower component relating to the polymer and the faster component (~3 × 10⁻^10^ m^2^·s^⁻1^) attributed to oligomers or small molecules formed in the deprotection stage (see section 4). The presence of small oligomers in PMC polymers could not be detected by aqueous SEC and this demonstrates the higher sensitivity of diffusion NMR spectroscopy.

# **2. Experimental Procedures**

### **2.1 Reagents.**

Reagents were commercially available from Merck (Sigma Aldrich) unless noted and were used without further purification. Mg(BHT)_2_(THF)_2_ had been synthesised previously according to previous literature.^[7]^ Before being introduced to the glovebox, Bn-MPA and Mg(BHT)_2_(THF)_2_ were dried in a desiccator over P_2_O_5_ under static vacuum for a week. 4-methoxybenzyl alcohol was dried with CaH_2_ overnight before being transported into the glovebox.

### **2.2 Synthetic Procedures.**

***Synthesis of benzyl 3-hydroxy-2-(hydroxymethyl)-2-methylpropanoate (Bn-MPA)***. Synthesized according to previous literature.^[8]^ In a 250 mL round bottom flask (RBF), bis-MPA (25 g, 186.4 mmol) was dissolved in 140 mL DMF to which KOH (11.94 g, 212.8 mmol, 1.14 equiv) was then added. Reaction was stirred for 1 h at 100 °C. Benzyl bromide (26 mL, 224 mmol, 1.2 equiv) was added dropwise and left to react overnight. Then DMF was removed *via* vacuum, facilitated by heating the RBF to 60 °C. The remaining solid was then dissolved in 500 mL DCM and transferred to a separating funnel. The organic layer was washed twice with 500 mL H_2_O, dried over MgSO_4_, filtered, and concentrated in *vacuo*. The crude product was recrystallized in toluene, affording the title compound as white crystals (22.5 g, 54%).

^1^H NMR (400 MHz, CDCl_3_, 298 K) *δ* 7.41 – 7.30 (m, 5H), 5.22 (s, 2H), 3.94 (d, *J* = 11.3 Hz, 2H), 3.74 (d, *J* = 11.3 Hz, 2H), 1.08 (s, 3H).

^13^C NMR (101 MHz, CDCl_3_, 298 K) *δ* 128.63, 128.29, 128.01, 77.39, 77.10, 76.94, 66.84, 49.24, 17.16

***Synthesis of 5-methyl-5-benzyloxycarbonyl-1,3-dioxan-2-one (Bn-MBC)***. Synthesized according to previous literature.^[9]^ In a 2-neck RBF, Bn-MPA (20 g, 89 mmol) was dissolved in 100 mL of CH_2_Cl_2_ to which pyridine (43 mL, 533.8 mmol, 6 equiv) was added. The mixture was submerged in an ice bath and left to stir for 1 h. Triphosgene (13.2 g, 44.5 mmol, 0.5 equiv) was solubilized in CH_2_Cl_2_ in a vial and added dropwise to the solution *via* dropping funnel over 1 h. After, the reaction was brought to room temperature, quenched with 140 mL of NH_4_Cl solution and transferred to a separating funnel. The organic layer was washed with saturated NaHCO_3_, dried over MgSO_4_, filtered, and concentrated in *vacuo*, to give yellow crystals. The crude product was recrystallized with hot EtOAc to give the title compound as white crystals (16 g, 71%). Crystals were further dried with P_2_O_5_ in a desiccator.

^1^H NMR (400 MHz, CDCl_3_, 298 K) *δ* 7.44-7.32 (m, 5H), 5.12 (s, 2H), 4.27 (s, 3H), 1.22 (s, 3H).

^13^C NMR (101 MHz, CDCl_3_, 298 K) *δ* 128.80, 128.78 (2C), 128.25 (2C), 77.35, 77.03, 76.72, 72.95 (2C), 67.94, 40.24, 17.62.

***Synthesis of poly(5-methyl-5-benzyloxycarbonyl-1,3-dioxan-2-one) (PMBC)***. In the glovebox, Bn-MBC (1 g, 4 mmol, 1 equiv) was dissolved in CHCl_3_ (6.75 mL, 0.5 M) in a vial. A stock solution of 4-MeBnOH was made of 10 µl initiator in 500 µl of CHCl_3,_ of which 5 µl (DP target 100) was added to the reaction mixture. Then Mg(BHT)_2_(THF)_2_ (0.012 g, 0.08 mmol, 0.5 equiv) was dissolved in 1 mL of CHCl_3_ and added to the reaction mixture. After reaching 90% conversion, the vial was removed from the glovebox and the reaction was quenched by the addition of two drops of trifluoracetic acid. The solution was precipitated twice in cold MeOH and dried under vacuum to afford the white solid polymer (0.81 g, 86%).

Representative ^1^H NMR (400 MHz, CDCl_3_, 298 K) *δ* 7.35 – 7.29 (m, 9H), 6.91 (*J* = 8.6 Hz, 2H), 6.88 (d, *J* = 8.6 Hz, 2H), 5.15 (s, 2H), 4.29 (t, *J* = 2.5 Hz, 4H), 1.25 (s, 3H).

Representative ^13^C NMR (101 MHz, CDCl_3_, 298 K) *δ* 200.72, 128.60, 128.35, 128.01, 102.47, 97.74, 77.34, 77.02, 76.71, 76.70, 68.60, 67.07, 46.56, 45.29, 17.41.

PMBC (DP 70): SEC (Chloroform with 0.5 % NEt_3_, PS standard): *M*_w_= 25 kg mol^-1^, *Ð*_M_ = 1.30

***Synthesis of poly(5-methyl-5-carboxyl-1,3-dioxan-2-one) (PMC)***. In a 100 mL RBF, PMBC (0.4 g) was dissolved in EtOAc (15 mL). After degassing the mixture, Pd/C (0.04 g, 10 wt. %) was added to the mixture under N_2_ environment. H_2_ was added to the system *via* a balloon and the reaction was left to stir overnight. The solution was centrifuged to remove the catalyst Pd/C and was concentrated *in vacuo* to obtain a transparent film of the solid deprotected polymer (0.227 g, 93%).

Representative ^1^H NMR (400 MHz, DMSO-*d*_6_, 298 K) *δ* 13.01 (s, 1H), 4.24 – 4.13 (m, 4H), 1.13 (s, 3H).

Representative ^13^C NMR (101 MHz, DMSO-*d*_6_, 298 K) *δ* 173.51, 153.96, 68.86, 54.91, 45.64, 40.15, 39.94, 39.73, 39.52, 39.31, 39.10, 38.89, 16.92.

PMC (DP 70): SEC (80:20 H_2_O: MeOH, 0.1 M NaNO_3_, PEG standard): *M*_w_ = 19 kg mol⁻^1^, *Ð*_M_ = 1.29

**Degradation studies**

***Hydrolysis***. 100 mL of 0.1 M sodium carbonate buffer solution (NaBS) was synthesized from 0.095 g of sodium carbonate anhydrous and 0.764 g of sodium bicarbonate, which was dissolved in 100 mL deuterated water in a 100 mL volumetric flask. PMC was dissolved in 0.1 M NaBS (pH 9.1) at a concentration of 2 mg·mL⁻^1^ in a 20 mL vial. Sample was incubated at 19 ± 1 °C and shaken at 250 rpm. For SEC measurements, aliquots of 100 µL were taken and dissolved in 0.9 mL of 80:20 MeOH: H_2_O, 0.1 M NaNO_3_ and analyzed on the aqueous SEC. For diffusion NMR spectroscopy, aliquots were taken and diluted with NaBS to 1, 0.5, 0.125 mg·mL⁻^1^ for 0 and 1 time points and were diluted to 1, 0.5, 0.25 mg·mL⁻^1^ for the remaining time points, in order to shorten instrument time.

***Bacterial plates***. Bacterial colonies of D5 alpha *E. coli* (Lot number 27465248) were stored at -180 °C before use. 1 mL of SOC media and incubated for 30 mins at 37 °C with continuous shaking at 180 rpm. After, 50 µL of this culture was transferred onto the agar plate (4% LB agar plate or 1% pure agarose plate).

***Biodegradation***. Bacterial colonies of D5 alpha *E. coli* (Lot number 27465248) were stored at -180 °C before use. For polymer biodegradation: *E. coli* was thawed slowly over ice, once melted, 50 µL of solution was taken and transferred to 1 mL of SOC media and incubated for 30 mins at 37 °C with continuous shaking at 180 rpm. After, 100 µL of this culture was transferred to the pre-warmed polymer solution (2 mg·mL⁻^1^ in 0.05 M PBS prepared in H_2_O). For diffusion NMR spectroscopy, aliquots were taken and filtered with a 0.22 µm Nylon filter. The aliquot at a concentration of 2 mg·m⁻^-1^ was measured *via* diffusion NMR spectroscopy and diluted with deionized water to 1 and 0.5 mg·mL⁻^1^.

***Control***. Polymer was dissolved in 0.05 M PBS solution (2 mg·mL⁻^1^, DI H_2_O). Ampicillin sodium salt (0.5 mg, 0.083 equiv.) was added to the solution to prevent any bacterial contamination. Samples were kept at 37 °C with continuous shaking at 180 rpm.

### **2.3 Characterization Techniques.**

***^1^H NMR spectroscopy***. All ^1^H NMR spectra were performed at 298 K on Bruker DPX -300 NMR instruments equipped operating at 300 MHz for ^1^H (100.57 MHz for ^13^C) unless stated otherwise. ^1^H NMR spectra are references to residual protic solvents (CHCl_3_ at δ =7.26 ppm, DMSO at δ =2.5, H_2_O at δ =4.6) and ^13^C NMR spectra are referenced to the residual solvent signal (CDCl_3_ at δ =77.16 ppm, DMSO-d_6_ at δ = 39.52). The resonance multiplicities are described as s (singlet), d (doublet), t (triplet) or m (multiplet).

***Diffusion NMR Spectroscopy***. Diffusion NMR measurements of samples were performed on a Bruker AVANCE III HD 300 spectrometer, equipped with a 7 T vertical wide-bore superconducting magnet, operating at a proton resonance frequency of 300.13 MHz, with a 10 mm ^1^H diff30 radiofrequency (RF) coil. NMR experiments were performed at 295 ± 1 K, controlled by the temperature of the water-cooled gradient coils. The 90° RF pulse was calibrated for each sample and found to be 20 ± 1 µs. The gradient system was calibrated by measuring the diffusion coefficient (*D*) of *n*-octane (2.16 ×10⁻^9^ m^2^·s⁻^1^ at 293 ± 1 K) which agreed with previous literature reported.^[10]^ Self-diffusion coefficients were measured using a pulsed gradient stimulated echo (PGSTE) sequence with 16 gradient steps. Additionally, 12, 10 and 8 gradient steps were also used in cases to shorten instrument time. Parameters for PMBC, PMC, PMC hydrolysis and biodegradation were optimized for each time point and concentration and reported in the supporting information. Diffusion coefficients were calculated by fitting the signal attenuation against the gradient, using Prospa (version 3.1, Magritek, Wellington, New Zealand) and were fitted to the Stejskal-Tanner equation^[1]^ using the KaleidoGraph (**Eq S1**).

***Size Exclusion Chromatography (SEC)***. Non-aqueous SEC measurements were performed in CHCl_3_ on an Agilent 1260 Infinity II Multi-Detector GPC/SEC System fitted with RI, ultraviolet (UV, λ =309 nm), and viscometer detectors. The polymers were eluted through an Agilent guard column (PLGel 5 µM, 50 × 7.5 mm) and two Agilent mixed -C columns (PLGel 5 µM, 300 × 7.5 mm) using CHCl_3_ (buffered with 0.5 % NEt_3_) as the mobile phase (flow rate= 1 mL min^-1^, 40 °C). *M*_n_, *M*_w_ and *Ð*_M_ = *M*_w_/*M*_n_ were determined using Agilent GPC/SEC software (vA.02.01) against a 15-point calibration curve (*M*_p_ = 162 – 3,187,000 g mol^-1^) based on polystyrene standards (Easivial PS-M/H, Agilent).

Aqueous SEC measurements were performed in 80:20 H_2_O and MeOH solution containing 0.1 M NaNO_3_ on an Agilent 1260 Infinity II Multi-Detector GPC/SEC System fitted with RI, ultraviolet (UV, λ =309 nm), and viscometer detectors. The polymers were eluted through an Agilent guard column (PLGel 5 µM, 50 × 7.5 mm) and two Agilent mixed -C columns (PL aquagel-OH MIXED-H 8 µM, 300 × 7.5 mm) with a flow rate= 1 mL min^-1^, 40 °C. *M*_n_, *M*_w_ and *Ð*_M_ = *M*_w_/*M*_n_ were determined using Agilent GPC/SEC software (vA.02.01) against a 15-point calibration curve (*M*_p_ = 615 – 3,187,000 g mol^-1^) based on PEG standards (Easivial PEG-M/H, Agilent).

Size Exclusion Chromatography Multi Angle Light Scattering (SEC-MALS) measurements were performed in DMF on a system equipped with an isocratic pump (1260 Infinity II, Agilent, Santa Clara, CA, USA), a multiangle static light scattering (MALS) detector with a 658 nm light source (DAWN HELEOS-II, Wyatt Technology, Santa Barbara, CA, USA), and a differential refractometer (DRI) detector operating at a wavelength of 658 nm (Optilab T-rEX, Wyatt Technology, Santa Barbara, CA, USA). Separations were performed using serially connected size exclusion columns (two PLgel MIXED-C columns, 10 µm, 7.5 × 300 mm, Agilent, Santa Clara, CA, USA) at 40 °C, with DMF (containing 5 mM NH_4_BF_4_) as a mobile phase at a flow rate of 1 mL/min. Samples were filtered using a 0.22 µm PTFE filter before injection. Absolute molecular weights (MWs) of polymers were determined using the ASTRA 7 software (version 7.1.3, Wyatt Technology, Santa Barbara, CA, USA) and calculated from dn/dc values of each polymer sample.

***Liquid Chromatography-Mass Spectrometry (LC-MS)***. LC-MS measurements were conducted on Waters XEVO G2-XS TOF, fitted with UV detectors (λ = 210-400 nm) The Mobile phase was 70:30 to 40:60% H_2_O:ACN, with gradient at 10 min and flow rate of mobile phase was 0.2 mL min^-1^. High resolution spectra used a lock-mass to adjust the calibrated mass scale. Electro spray ionization technique time of flight (-ve) mode was additionally used.

***Rheology***. Viscosity measurements were performed on an Anton Paar MCR 302 equipped with a PP50 geometry. The temperature was controlled with a P-PTD 200/AIR Peltier and a P-PTD 200 hood. Sample was loaded directly onto plate, and plate was lowered to ensure no bubbles formed. The method used was for low-viscosity liquids with frequency sweeps were performed from 1 to 100 rad/s. Shear rate sweeps were performed from 1 to 100 s^-1^ at 1 s^-1^ per shear rate.

### **2.4 Diffusion NMR spectroscopy parameters.**

**PMC Hydrolysis (pH 9.1 (D_2_O), 19 °C)**

Diffusion measurements were optimised for the polymer proportion. Therefore, samples were collected with a maximum gradient (*G*_max_) of 2.8 T·m^–1^, minimum gradient (*G*_min_) of 0.14 T·m^–1^, pulse duration (*δ*) of 2 ms, observation (*Δ*) of 100 ms, with a repetition time of 1 s. The maximum gradient was optimised to fit the degrading polymer sample. Therefore, from 9 – 57 days the *G*_max_ of 2.4 T·m^–1^ and *G*_min_ of 0.3 T·m^–1^ was used and for the 65 day time point a *G*_max_ of 1.8 T·m^–1^ *G*_min_ of 0.2 T·m^–1^ was used.

Diffusion coefficients were calculated by fitting the signal attenuation against the gradient, using Prospa (version 3.1, Magritek, Wellington, New Zealand) and were fitted to the Stejskal-Tanner equation using the KaleidoGraph (**Eq S1**).

**PMC biodegradation 1 (*E. coli*, 0.05 M PBS (DI H_2_O), 37 °C, 180 rpm)**

Parameters for the diffusion measurements of PMC biodegradation were adapted to ensure the maximum diffusion decay was measured. Parameters are listed in **Table S1**. The number of scans were 64 for 2 mg·mL^–1^, 128 scans for 1 mg·mL^–1^, and 256 for 0.5 mg·mL^–1^. The *G*_min_ was increased at lower concentrations to minimise the signal to noise (the interference of water in the polymer peaks).

**Table S1** Diffusion NMR spectroscopy parameters used in the biodegradation of PMC (E. coli, 0.05 M PBS (DI H_2_O), 37 °C, 180 rpm).

| Time / days | *G*_max_ / T·m^–1^ | *G*_min_ / T·m^–1^ | *δ* / ms | *Δ* / ms | Repetition time / s |
| --- | --- | --- | --- | --- | --- |
| 0 | 2 mg ·mL^–1^: 3  1, 0.5 mg·mL^–1^: 2.5 | 2 mg·mL^–1^: 0.5  1, 0.5 mg·mL^–1^: 0.75 | 2 | 100 | 1 |
| 3 | 2.5 | 2 mg·mL^–1^: 0.5  1, 0.5 mg·mL^–1^: 0.75 | 2 | 100 | 1 |
| 7 | 6 | 2 | 1 | 20 | 1 |
| 14 | 5 | 2 mg·mL^–1^: 1.5  1, 0.5 mg·mL^–1^: 2 | 1 | 20 | 1 |
| 21 | 2, 1 mg mL^–1^: 4.5  0.5 mg mL^–1^: 4 | 2 mg·mL^–1^: 2  1 mg·mL^–1^: 1.5  0.5 mg·mL^–1^: 1 | 1 | 20 | 1 |
| 28 | 5 | 2 mg·mL^–1^: 2  1, 0.5 mg·mL^–1^: 1.5 | 1 | 20 | 1 |
| 32 | 4 | 1.5 | 1 | 20 | 1 |
| 42 | 4 | 1.5 | 1 | 20 | 1 |

**PMC biodegradation 2 (*E. coli*, 0.05 M PBS (DI H_2_O), 37 °C, 180 rpm)**

Parameters for the diffusion measurements of PMC biodegradation were adapted to ensure the maximum diffusion decay was measured. Parameters are listed in **Table S1**. The number of scans were 64 for 2 mg·mL^–1^, 128 scans for 1 mg·mL^–1^, and 256 for 0.5 mg·mL^–1^. The *G*_min_ was increased at lower concentrations to minimise the signal to noise (the interference of water in the polymer peaks).

**Table S2** Diffusion NMR spectroscopy parameters used in the biodegradation of PMC (E. coli, 0.05 M PBS (DI H_2_O), 37 °C, 180 rpm).

| Time / days | *G*_max_ / T·m^–1^ | *G*_min_ / T·m^–1^ | *δ* / ms | *Δ* / ms | Repetition time / s |
| --- | --- | --- | --- | --- | --- |
| 0 | 2 mg·mL^–1^: 3  1, 0.5 mg·mL^–1^: 2.5 | 1.5 | 2 | 100 | 1 |
| 3 | 2.5 | 2, 1 mg·mL^–1^: 0.5  0.5 mg·mL^–1^: 0.75 | 2 | 100 | 1 |
| 7 | 6 | 2 | 1 | 20 | 1 |
| 13 | 5 | 1.5 | 1 | 20 | 1 |
| 21 | 4.5 | 1.5 | 1 | 20 | 1 |
| 42 | 4 | 1.5 | 1 | 20 | 1 |
| 63 | 4 | 1.5 | 1 | 20 | 1 |
| 7 | 3.5 | 1.5 | 1 | 20 | 1 |

**PMC biodegradation (*E. coli*, 0.05 M PBS (DI H_2_O), 37 °C, 180 rpm) with continuous removal of degradation product**

Parameters for the diffusion measurements of PMC biodegradation were adapted to ensure the maximum diffusion decay was measured. Parameters are listed in **Table S2**. The number of scans were 128 for 1 mg·mL^–1^, 256 scans for 0.5 mg·mL^–1^, and 512 for 0.25 mg·mL^–1^.

**Table S3** Parameters for the diffusion NMR spectroscopy measurements of PMC with continuous removal of the degradation product (E. coli, 0.05 M PBS (DI H_2_O), 37 °C, 180 rpm).

| Time / days | *G*_max_ / T·m^–1^ | *G*_min_ / T·m^–1^ | *δ* / ms | *Δ* / ms | Repetition time / s |
| --- | --- | --- | --- | --- | --- |
| 0 | 10 | 2 | 1 | 20 | 1 |
| 7 | 7 | 2 | 1 | 20 | 1 |
| 14 | 6 | 2 | 1 | 20 | 1 |
| 21 | 5 | 2 | 1 | 20 | 1 |

**PMC biodegradation mixed with PEG (*E. coli*, 0.05 M PBS (DI H_2_O), 37 °C, 180 rpm)**

Parameters for the diffusion measurements of PMC biodegradation were adapted to ensure the maximum diffusion decay was measured. Parameters are listed in **Table S3**. The number of scans were 64 for 1 mg·mL^–1^, 128 scans for 2 mg·mL^–1^, and 256 for 1 mg·mL^–1^. In order to obtain good attenuation for both PEG and PMC separate experiment parameters were used from 14 days onwards.

**Table S4** Diffusion NMR spectroscopy parameters used in the biodegradation of PMC and PEG mixtures (E. coli, 0.05 M PBS (DI H_2_O), 37 °C, 180 rpm).

| Time / days | *G*_max_ / T·m^–1^ | *G*_min_ / T·m^–1^ | *δ* / ms | *Δ* / ms | Repetition time / s |
| --- | --- | --- | --- | --- | --- |
| 0 | 6 | 1 | 1 | 20 | 1 |
| 7 | 3 | 0.5 | 1 | 20 | 1 |
| 14 | PMC: 1.4  PEG: 3 | PMC: 0.7  PEG: 0.5 | 2 | 100 | 1 |
| 21 | PMC: 5  PEG: 3 | PMC: 1.5  PEG: 0.5 | PMC: 1  PEG: 2 | PMC: 20  PEG: 100 | 1 |
| 28 | PMC: 5  PEG: 10 | PMC: 1.5  PEG: | 2 | 100 | 1 |
| 32 | PMC: 1 mg·mL^–1^: 2.5  0.5, 0.25 mg·mL^–1^: 4  PEG: 10 | PMC: 1 mg·mL^–1^: 0.12  0.5, 0.25 mg·mL^–1^: 2  PEG:2 | PMC: 1  PEG: 2 | PMC: 20  PEG: 100 | 1 |
| 49 | PMC: 3.5  PEG: 2 | PMC: 1  PEG: 10 | PMC: 1  PEG: 2 | PMC: 20  PEG: 100 | 1 |

# **Appendix**

**
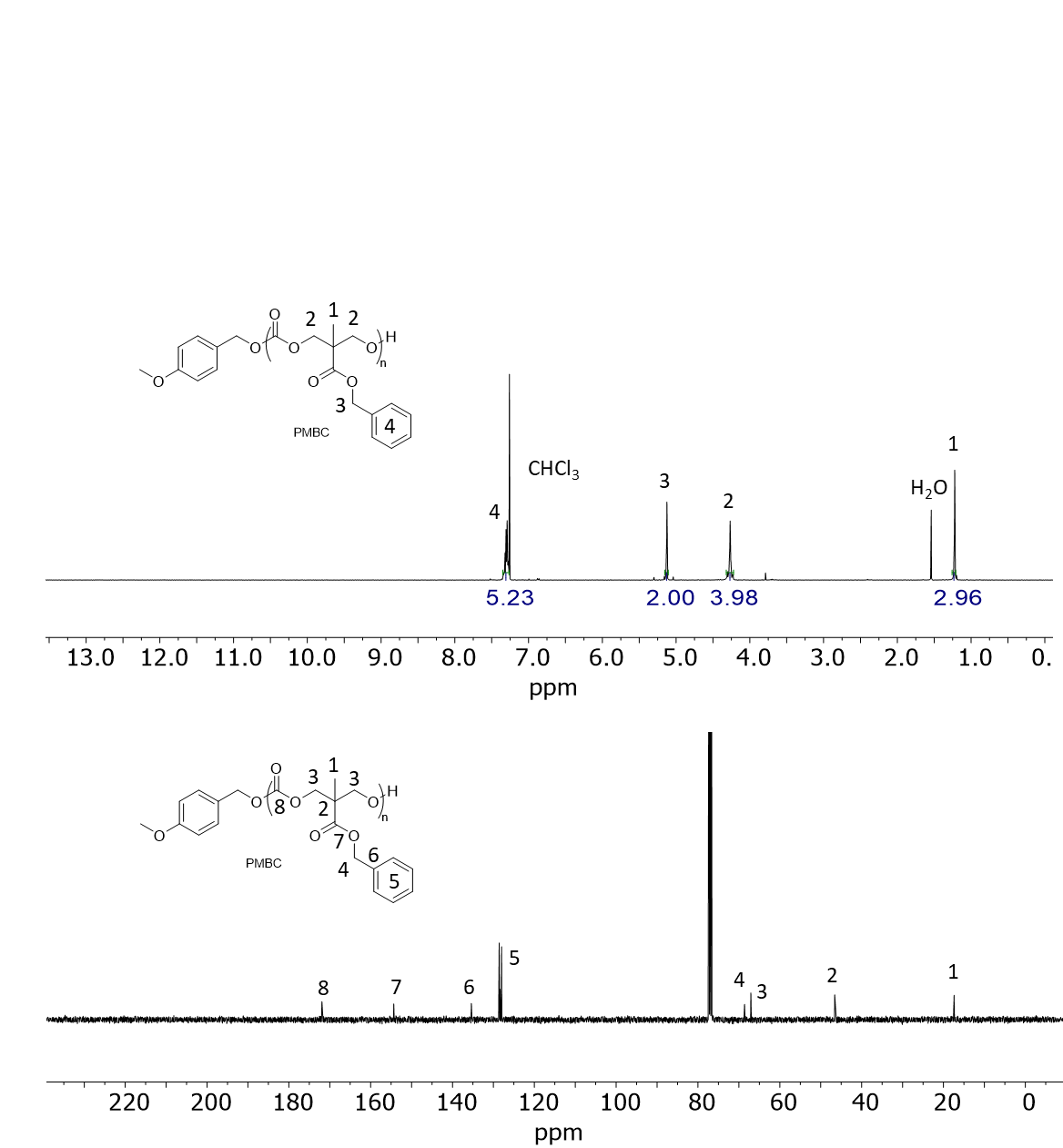
**

**Figure S1**. ^1^H NMR (CDCl_3_, 400 MHz, 298 K) and ^13^C NMR (100.57 MHz) of protected PMBC polymer, DP 240.


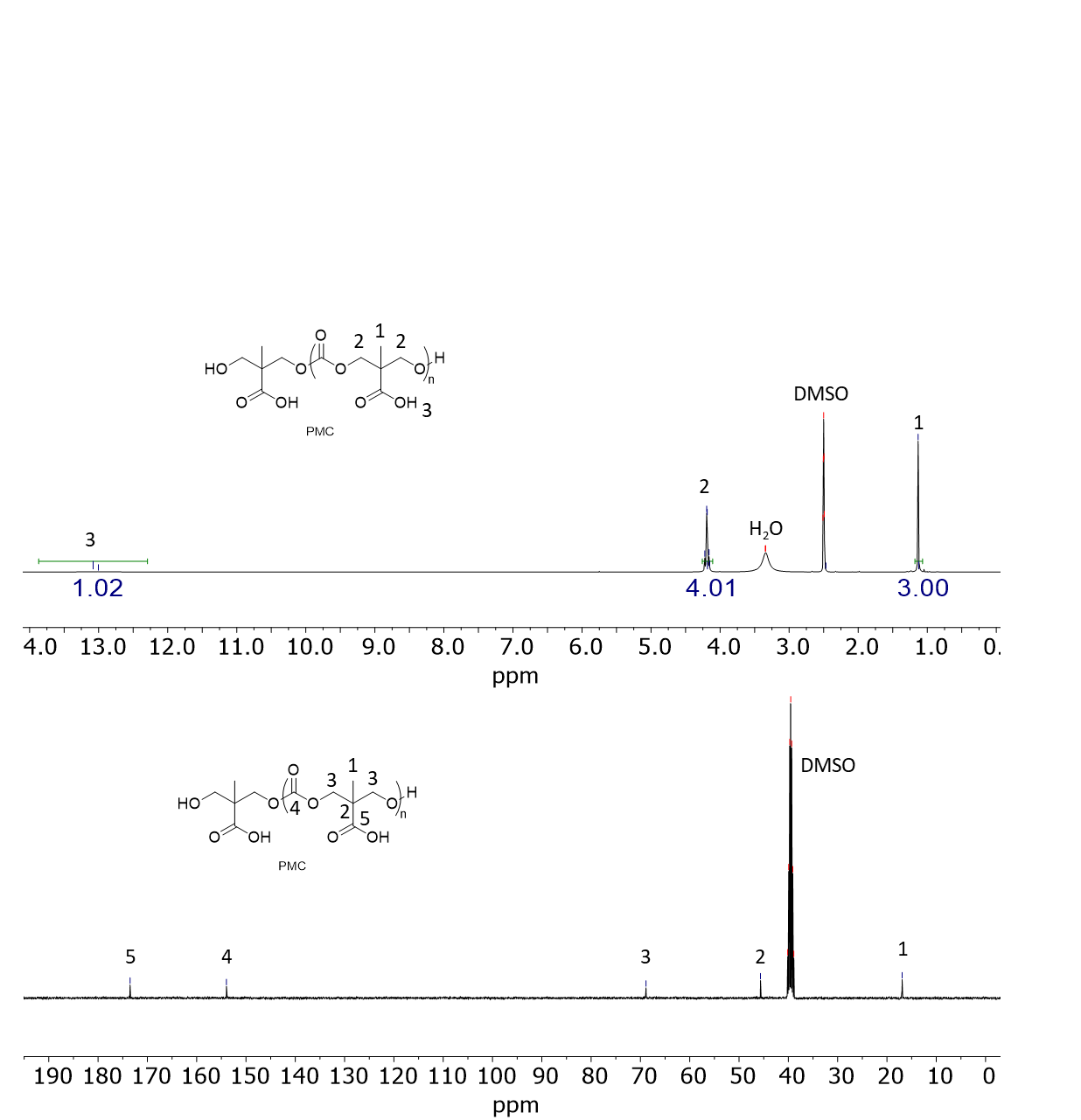


**Figure S2** ^1^H NMR (DMSO-d_6,_ 400 MHz, 298 K) and ^13^C NMR (100.57 MHz) of deprotected PMC, DP 240.


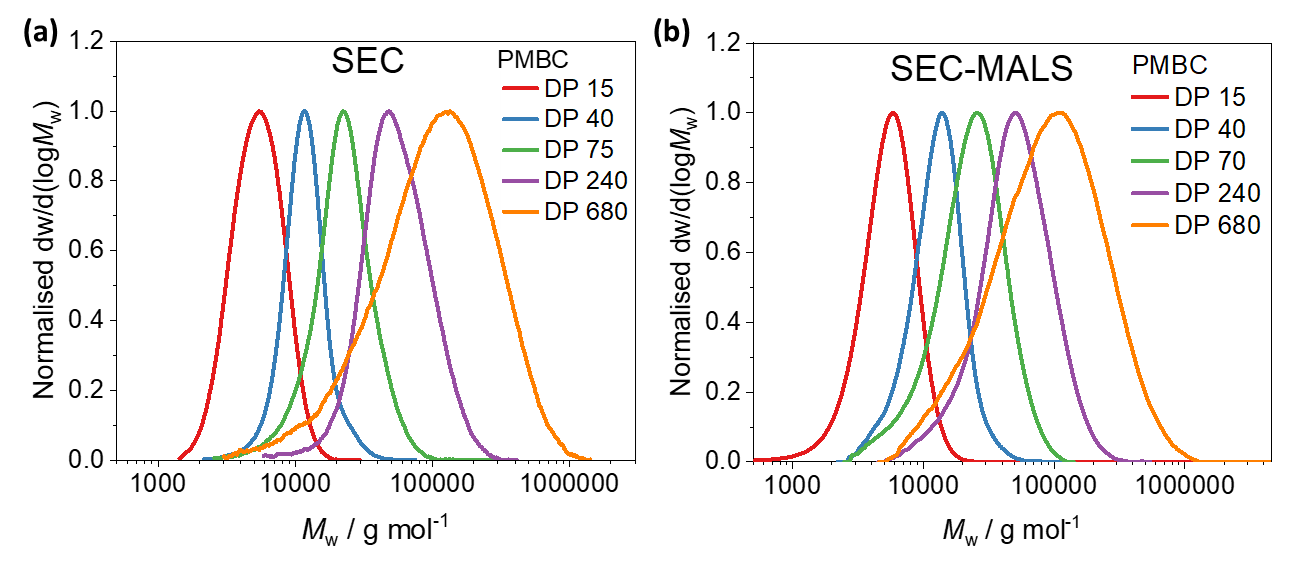


**Figure S3 (a)** Size exclusion chromatograms of protected PMBC polymers (CHCl_3_ with 0.5% NEt_3_, PS standards, RI detector) **(b)** Size exclusion chromatograms of PMBC polymers measured in DMF (containing 5 mM NH_4_BF_4_) coupled with a multi angle light scattering (MALS) detector.

**Figure S4** Size exclusion chromatograms of deprotected PMC polymers (80:20 H_2_O:MeOH, 0.1 M NaNO_3_, PEG standards, RI detector).

*
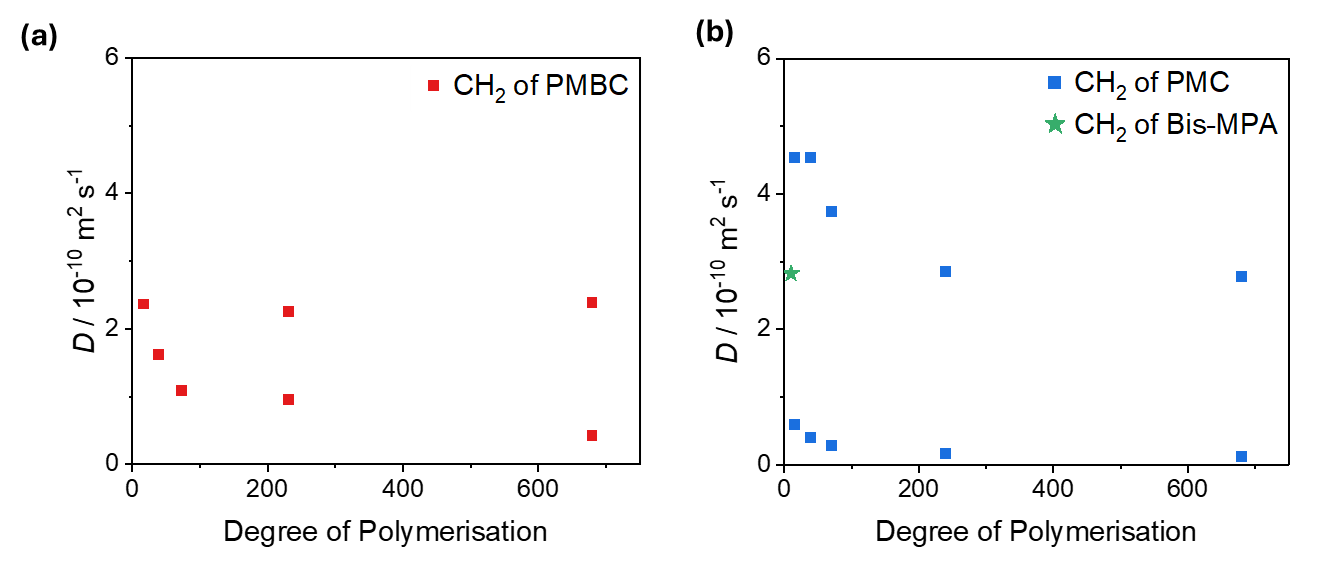
*

**Figure S5 (a)** Diffusion coefficients (D) for Bn-MBC monomer and protected PMBC (CH_2_ environment) in CDCl_3_ plotted against the degree of polymerisation (DP). **(b)** D for Bn-MBC and deprotected PMC (CH_2_ environment) in DMSO-d_6_ plotted against DP.

**Figure S6** Hydrodynamic radius of PMBC and PMC calculated from diffusion coefficients measured above via the Stokes-Einstein equation.


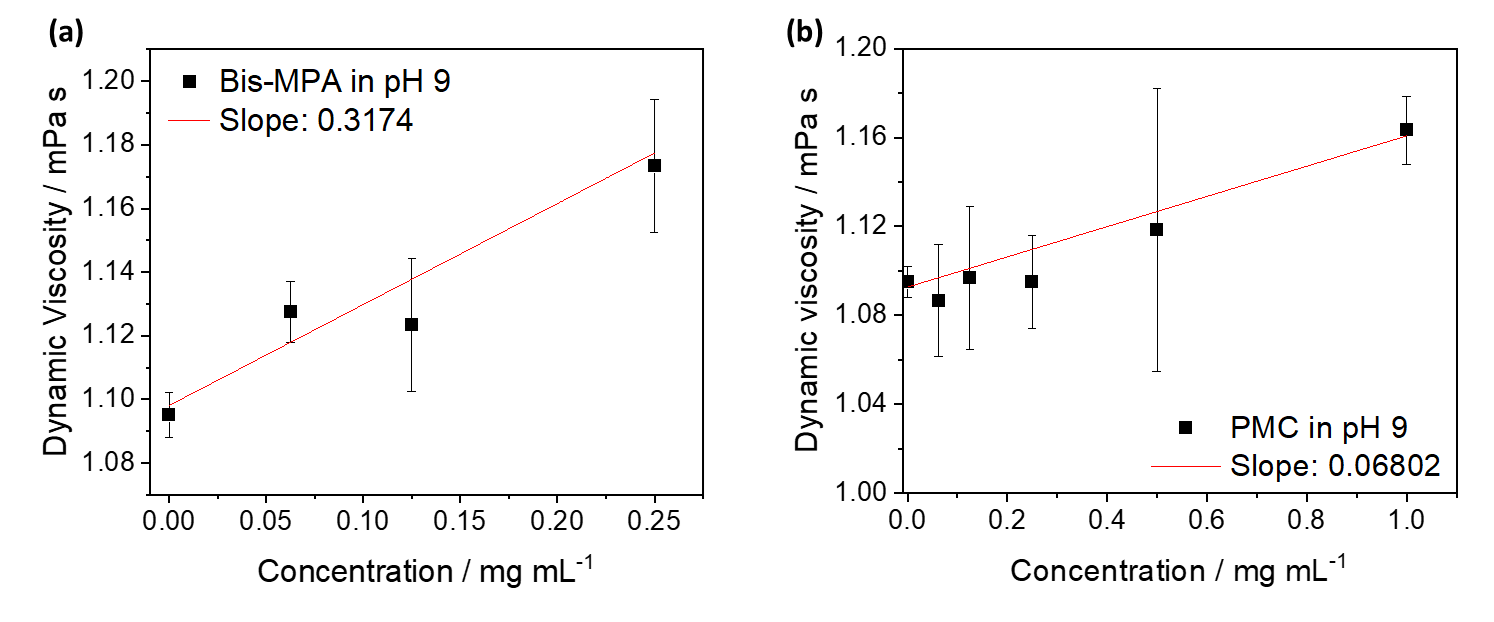


**Figure S7** Viscosity plotted against concentration of **(a)** bis-MPA and **(b)** PMC measured with a rheometer as described above.


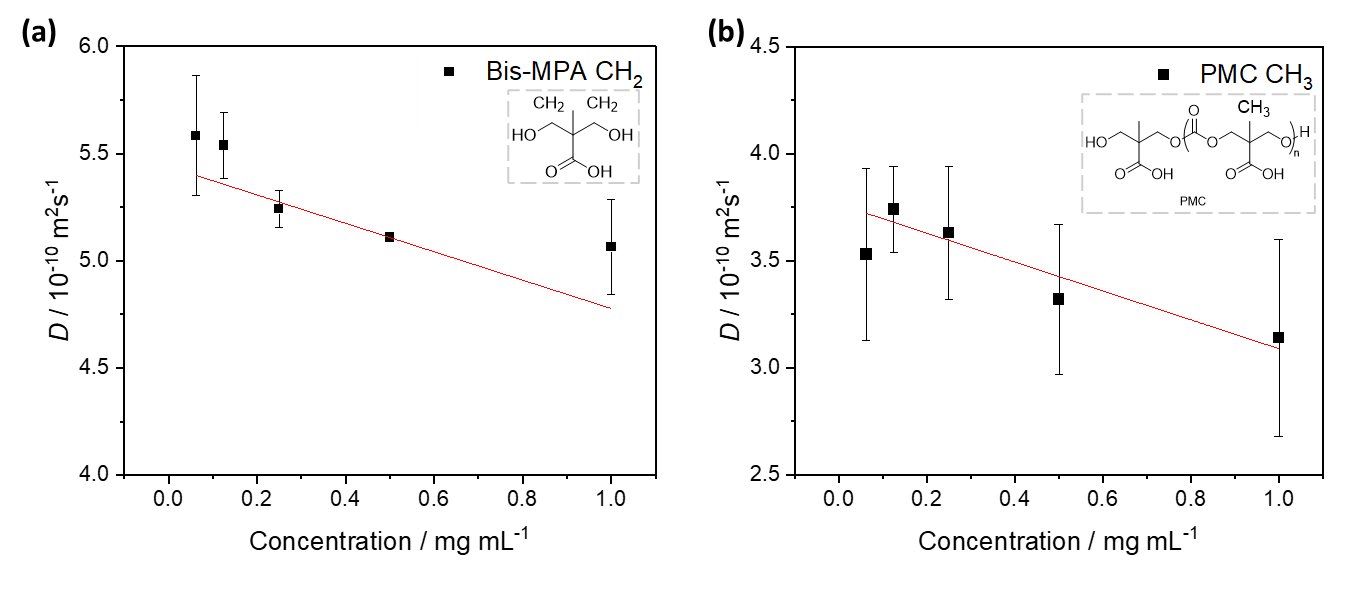


**Figure S8** Diffusion coefficient plotted against concentration of **(a)** bis-MPA and **(b)** PMC (300 MHz, 298 K, G_max_ = 2.8 T·m^–1^, G_min_ = 0.14 T·m^–1^, δ=2 ms, Δ= 100 ms, and repetition time = 1 s).

**Figure S9** Diffusion coefficient plotted against concentration of PMC polymers over time to extrapolate to infinite dilution over the 65-daydegradation period at 19 °C, NaBS pH 9.1 made up with D_2_O.

**Figure S10** Diffusion coefficient plotted for 4 PMC polymer samples (DP 240, H_2_O, 300 MHz, 298 K, G_max_ = 2.5 T·m^–1^, G_min_ = 0.75 T m^–1^, δ= 2 ms, Δ= 100 ms, and repetition time = 1 s) indicating the minimum variability of the diffusion NMR measurements.


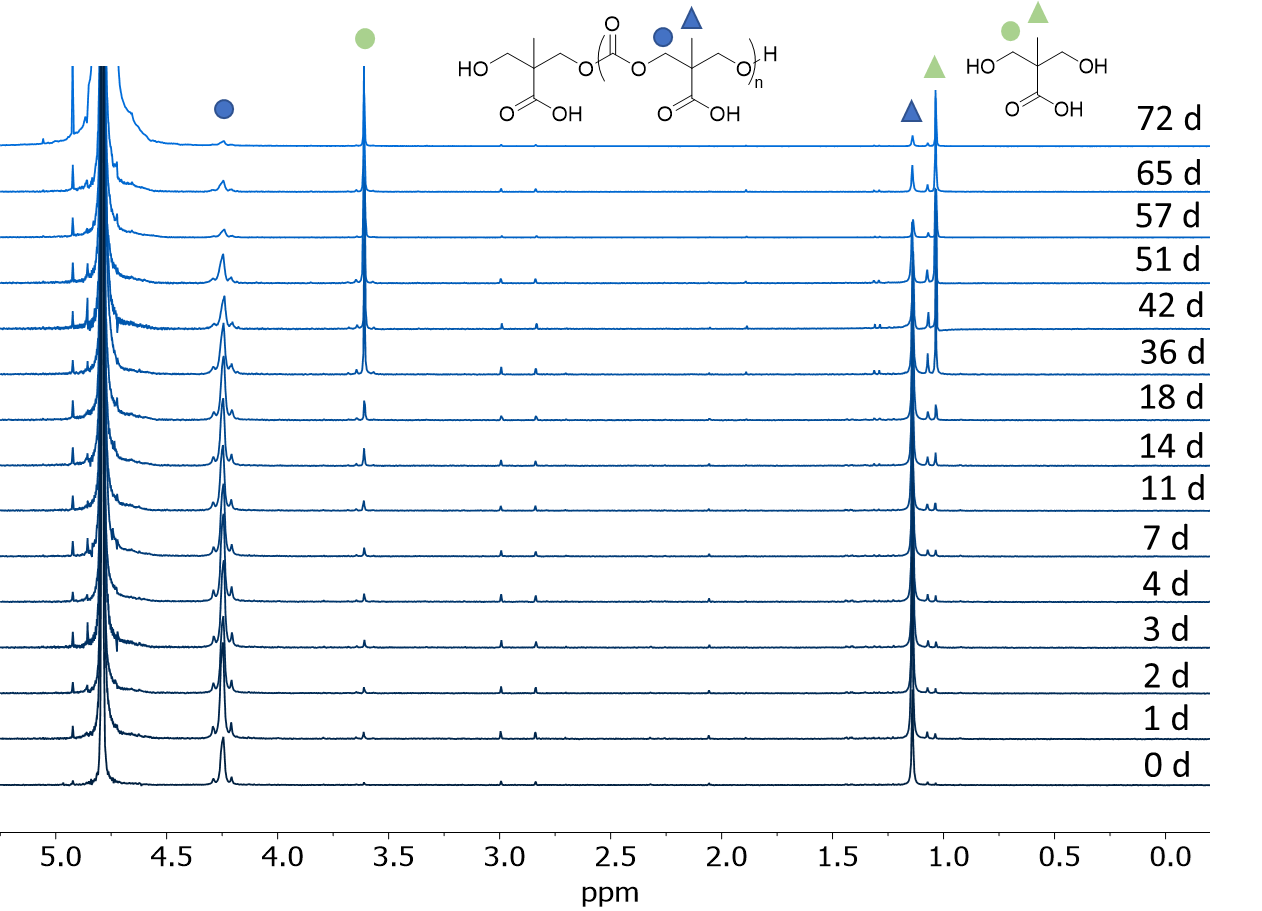


**Figure S11** Stacked ^1^H NMR spectra (300 MHz, D_2_O. 298 K) of PMC hydrolysis in NaBS (pH 9.1) with the identified peaks of PMC and bis-MPA.


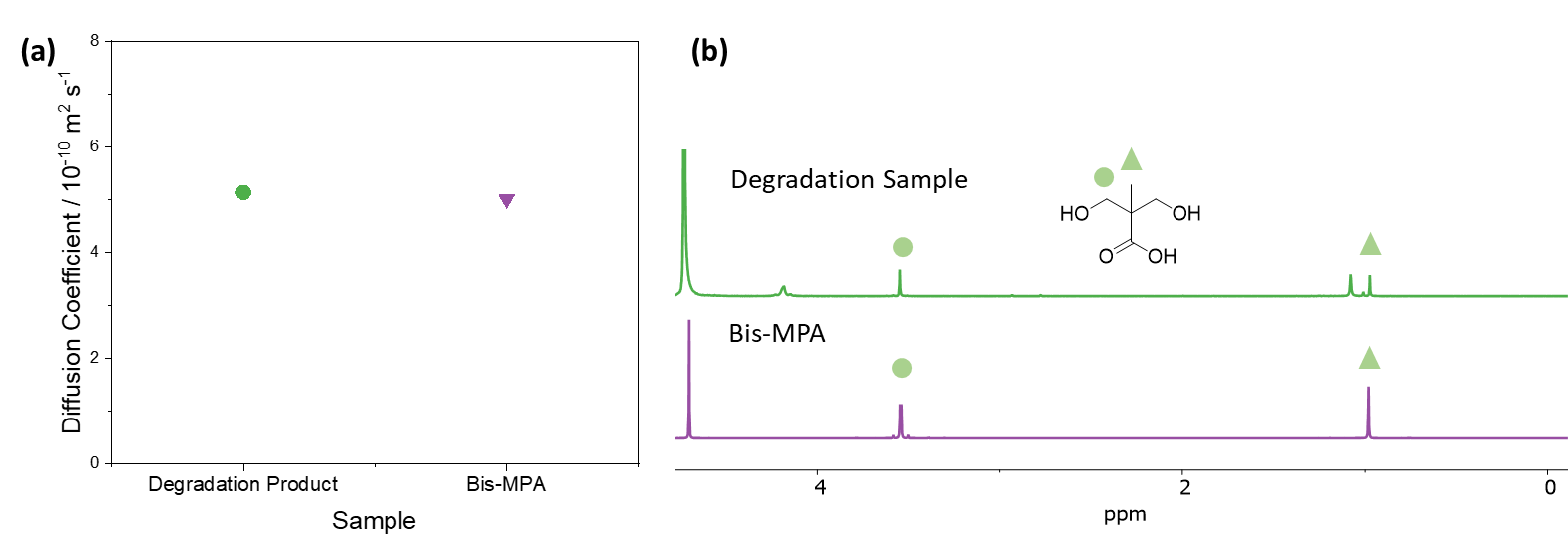


**Figure S12** Comparison of the diffusion coefficient of the degradation product and commercial bis-MPA **(a)** Diffusion coefficients (300 MHz, 298 K, G_max_ = 2.8 T·m^–1^, G_min_ = 0.14 T m^–1^, δ= 2 ms, Δ= 100 ms, and repetition time = 1 s) **(b)** ^1^H NMR spectra (300 MHz, D_2_O, 298 K).


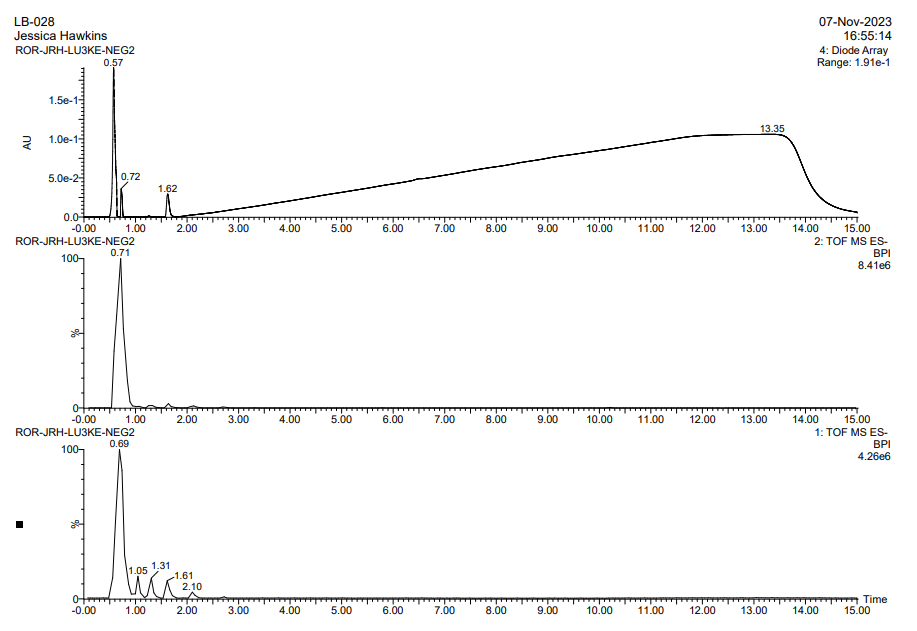


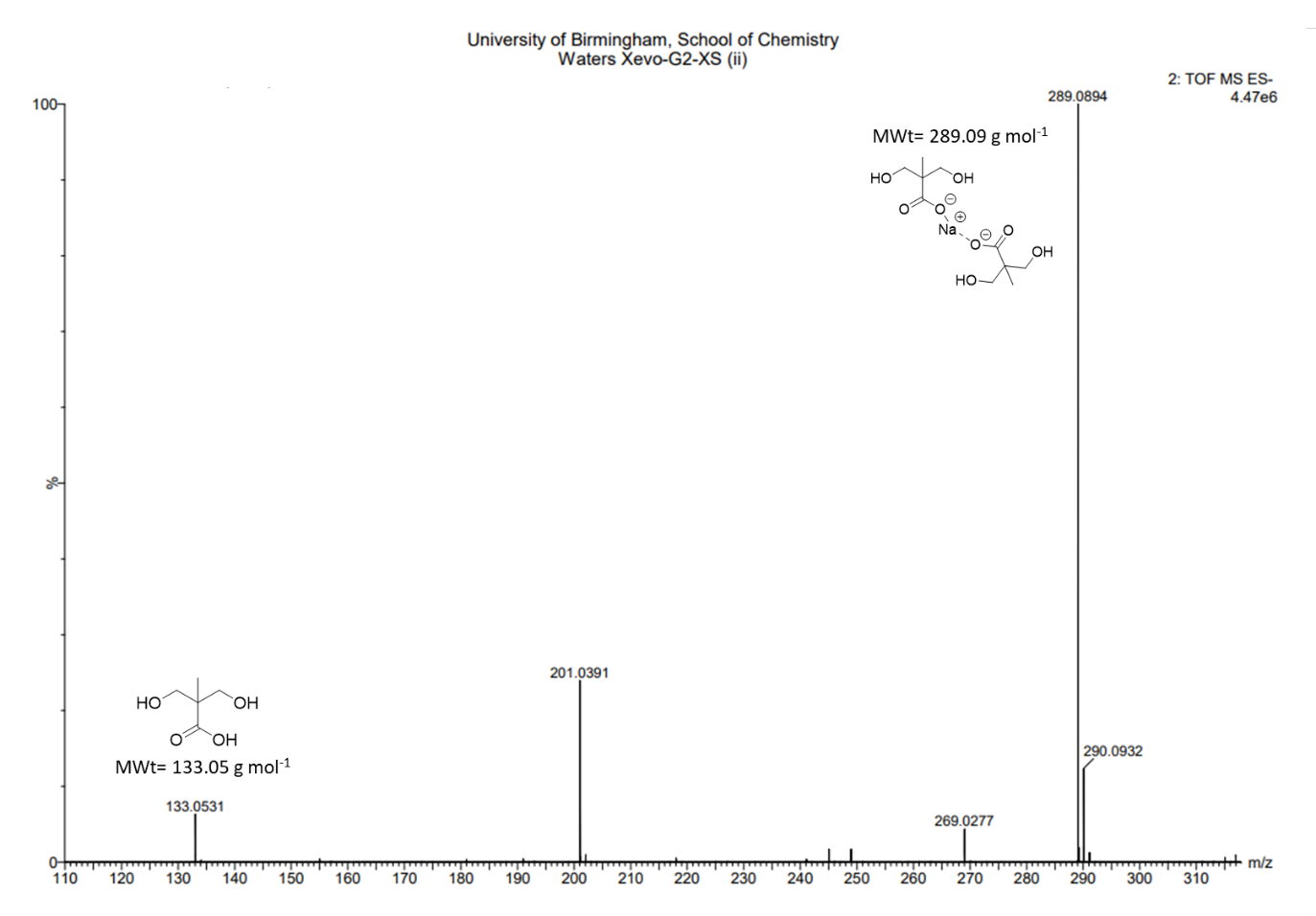


**Figure S13** LC-MS (ESI –TOF) (negative) of PMC degradation sample in pH 9.1 (D_2_O), 19 °C after 120 days. Mobile phase was 70:30 to 40:60% H_2_O:ACN, with gradient at 10 min and flow rate of mobile phase was 0.2 mL·min^–1^.

**Table S5** Showing diffusion coefficients at infinite dilution, log (molar mass), molar mass calculated from D, M_w_ and M_p_ measured with SEC (PEG calibrant) for PMC hydrolysis (19 °C, pH 9.1 D_2_O).

| Time / days | *D* / 10 ^–11^ m^2^s^–1^ | Log(*M*) | *M* *D* NMR | *M_w_* SEC | *M_p_* SEC |
| --- | --- | --- | --- | --- | --- |
| 0 | 3.16 | 4.761895 | 57796 | 45149 | 46994 |
| 1 | 3.18 | 4.758379 | 57330 | 44179 | 46065 |
| 2 | 3.43 | 4.691878 | 49190 | 43635 | 47308 |
| 3 | 3.52 | 4.669404 | 46709 | 42224 | 45760 |
| 4 | 3.85 | 4.588966 | 38812 | 40573 | 43392 |
| 7 | 4.04 | 4.547558 | 35282 | 35471 | 33107 |
| 11 | 4.63 | 4.427476 | 26759 | 28243 | 24033 |
| 14 | 4.95 | 4.368829 | 23379 | 26658 | 22227 |
| 18 | 5.15 | 4.335095 | 21632 | 22313 | 19518 |
| 23 | 6.05 | 4.194158 | 15637 | 19149 | 17591 |
| 30 | 7.42 | 4.014832 | 10347 | 13508 | 12713 |
| 36 | 8.85 | 3.859599 | 7238 | 11571 | 10944 |
| 42 | 9.47 | 3.800119 | 6311 | 10589 | 9989 |
| 51 | 10.9 | 3.674048 | 4721 | 9738 | 9235 |
| 57 | 11.0 | 3.66889 | 4665 | 10421 | 8592 |
| 65 | 11.9 | 3.60238 | 4003 | 8380 | 7941 |

**Figure S14** Size exclusion chromatograms (80:20 H_2_O:MeOH, 0.1 M NaNO_3_, PEG calibrants) for hydrolysis of PMC in pH 9.1, 19 °C monitored for 65 days.

**Figure S15** Inverse of the normalised molar mass of PMC vs time of PMC hydrolysis (19 °C, pH 9.1 D_2_O) comparing SEC and diffusion NMR spectroscopy showing the second order rate constant calculated via M_w_.


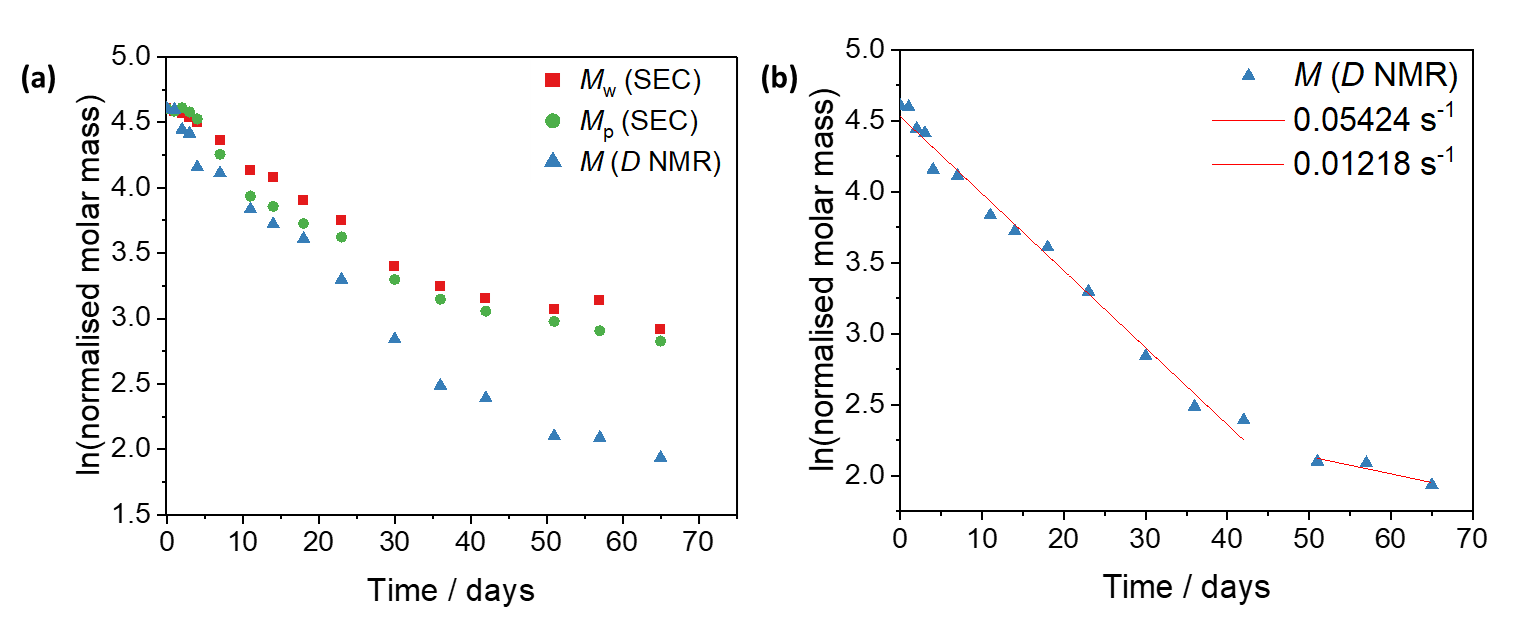


***Figure S16*** ***(a)*** *Natural log of the normalised molar mass of PMC vs time of PMC hydrolysis (19 °C, pH 9.1 D_2_O) comparing SEC and diffusion NMR spectroscopy showing the first order rate constant calculated via diffusion NMR spectroscopy* ***(b)*** *Natural log of the normalised molar mass of PMC vs time of PMC hydrolysis in the same conditions from diffusion NMR spectroscopy showing the two first order rate constants calculated via M_w_.*

**Table S6** Showing molar masses calculated from D, and SEC (M_w_ and M_p_) measured via the PMBC calibration curve for PMC hydrolysis (19 °C, pH 9.1 D_2_O).

| Time / days | *M* diffusion NMR | *M_w_* SEC | *M_p_* SEC |
| --- | --- | --- | --- |
| 0 | 57796 | 71226 | 68223 |
| 1 | 57330 | 70996 | 66247 |
| 2 | 49190 | 68691 | 68895 |
| 3 | 46709 | 65257 | 65601 |
| 4 | 38812 | 62016 | 60657 |
| 7 | 35282 | 50550 | 40592 |
| 11 | 26759 | 41850 | 26638 |
| 14 | 23379 | 33674 | 22332 |
| 18 | 21632 | 25234 | 18358 |
| 23 | 15637 | 20079 | 15695 |
| 30 | 10347 | 11764 | 9617 |
| 36 | 7238 | 9198 | 7677 |
| 42 | 6311 | 8161 | 6759 |
| 51 | 4721 | 7126 | 5951 |
| 57 | 4665 | 8152 | 5343 |
| 65 | 4003 | 7699 | 4797 |

**Figure S17** Natural log of the normalised molar mass of PMC over the 65-day degradation period (19 °C, pH 9.1 D_2_O).


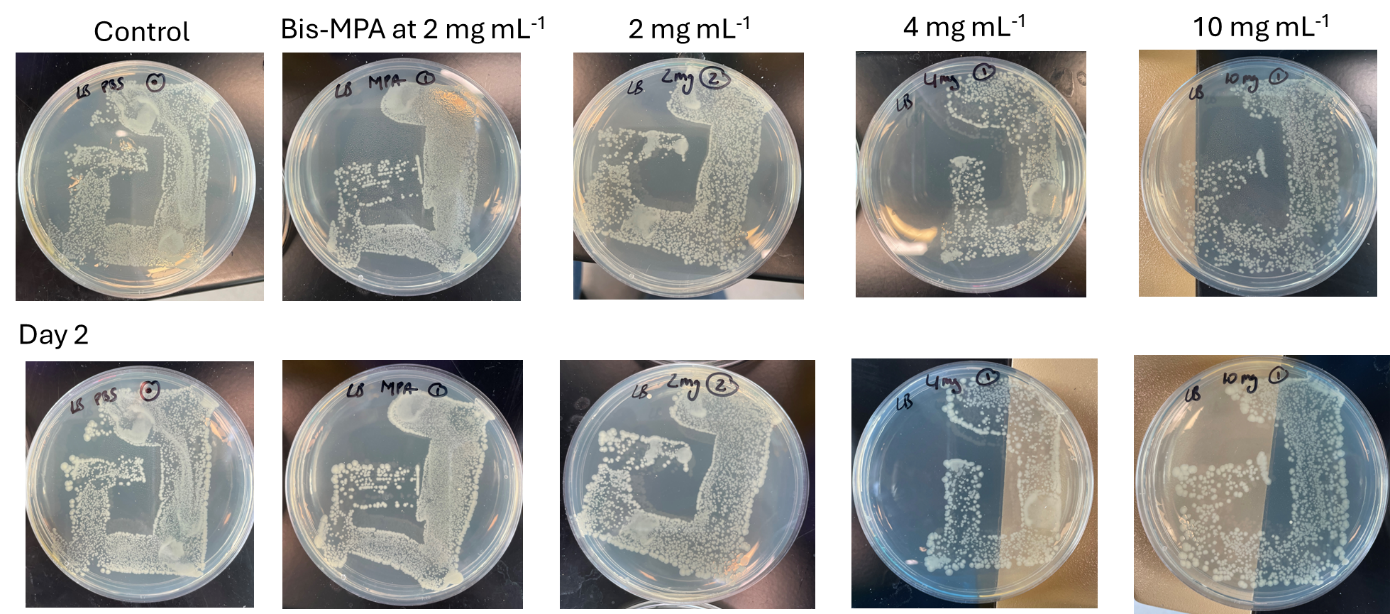


**Figure S18** E. coli growth on nutrient rich LB agar for control Bia-MPA and PMC at concentrations of 2, 4, 10 mg·mL^–1^, incubated at 37 °C for 2 days done in triplicates.


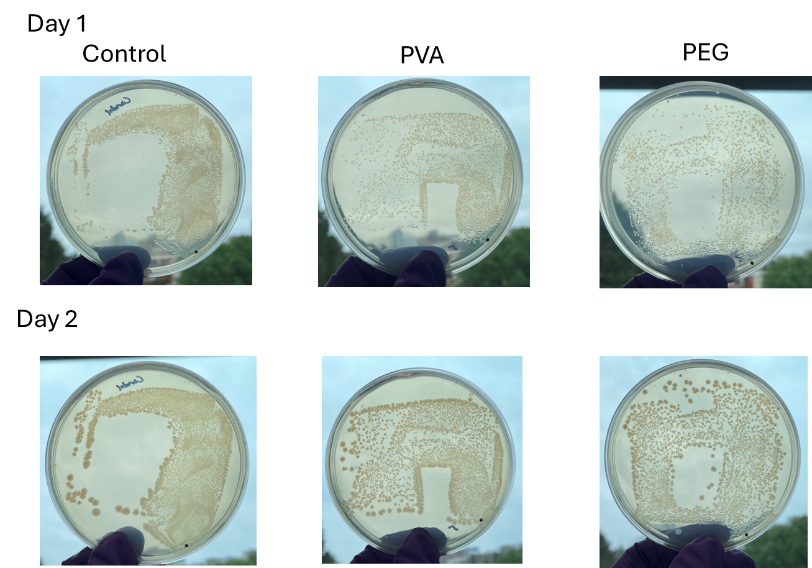


**Figure S19** E. coli growth on nutrient rich LB agar, incubated at 37 °C for 2 days for control, PVA and PEG at 2 mg·mL^–1^ performed in triplicates.


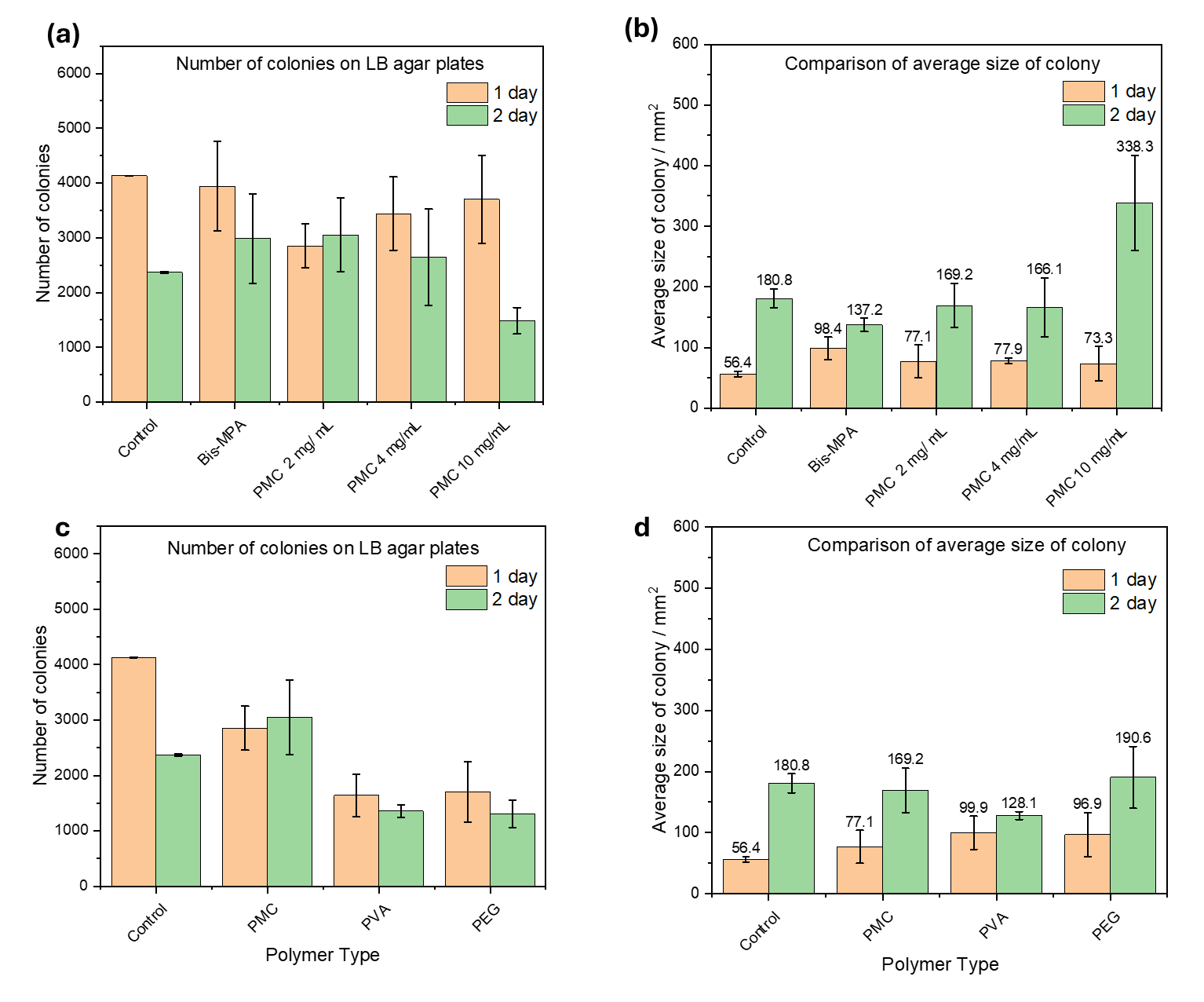


**Figure S20** Comparison of bacterial growth comparing **(a)** number of colonies **(b)** size of colonies **(c)** type pf polymer comparing number of colonies grown and **(d)** size of colonies. All plates were stored at 37 °C and performed in triplicates.


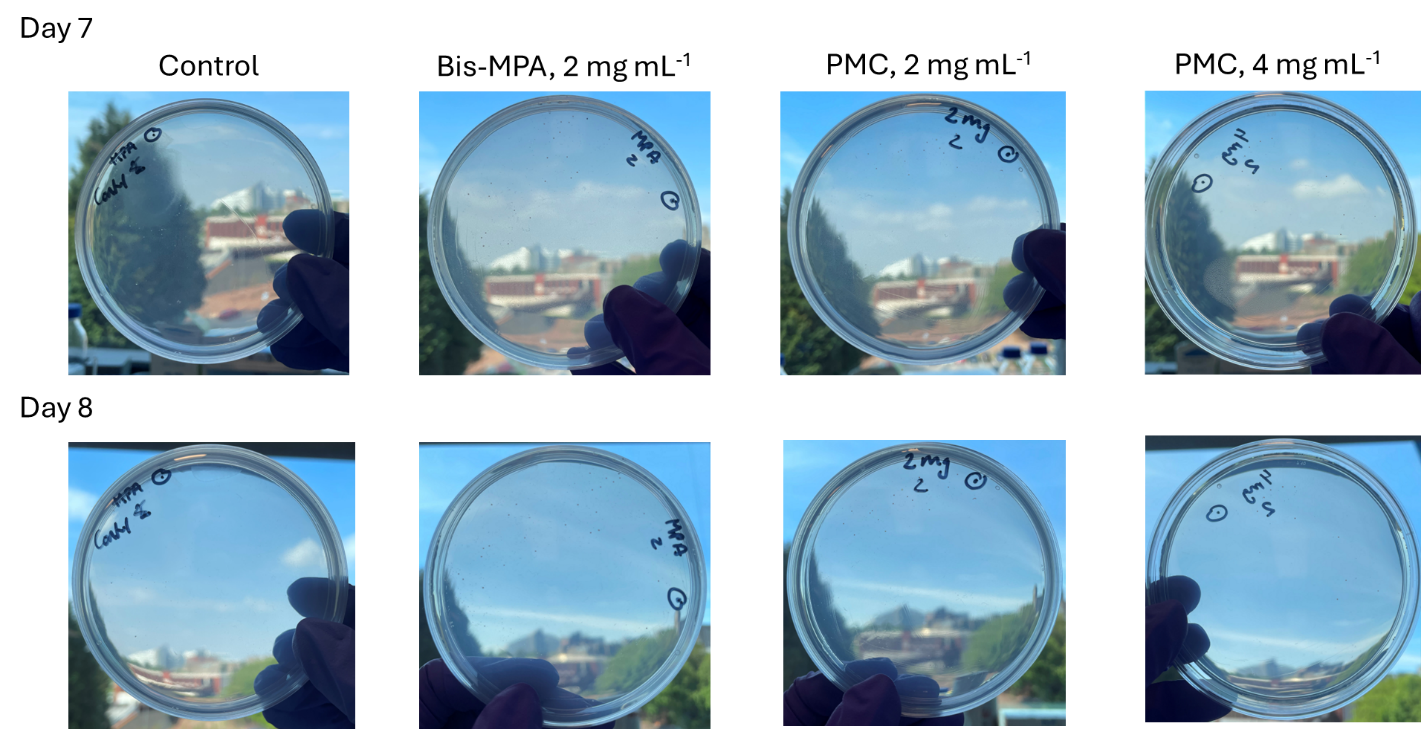


**Figure S21** E. coli growth on pure agarose plates with control Bia-MPA and PMC at concentrations of 2, 4, 10 mg·mL^–1^ as food source, incubated at 37 °C for 8 days performed in triplicates.


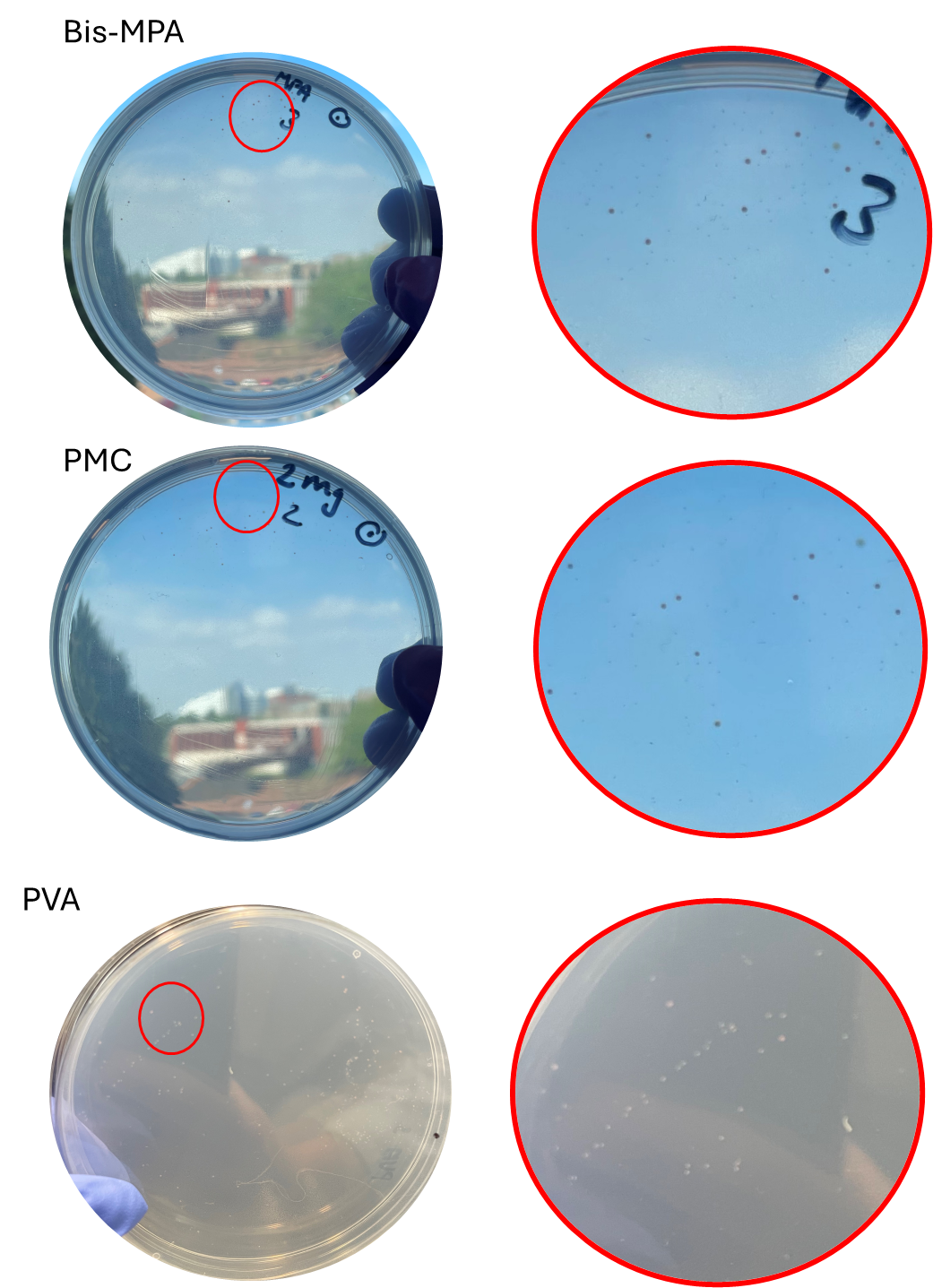


**Figure S22** Representative pure agarose plates of bis-MPA, PMC and PVA (2 mg·mL^–1^) after 7 days stored at 37 °C.


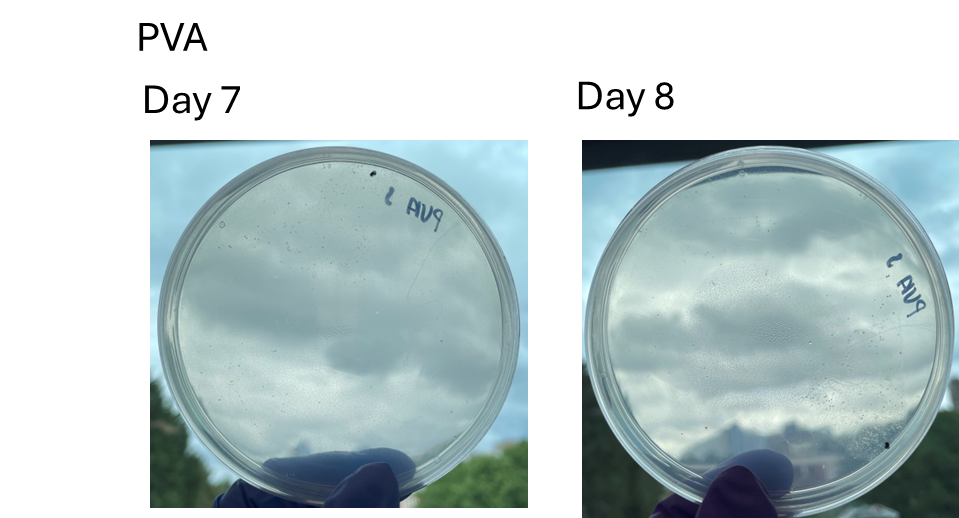


**Figure S23** E. coli growth on pure agarose plates with PVA (2 mg·mL^–1^) as food source incubated at 37 °C for 8 days done in triplicates.


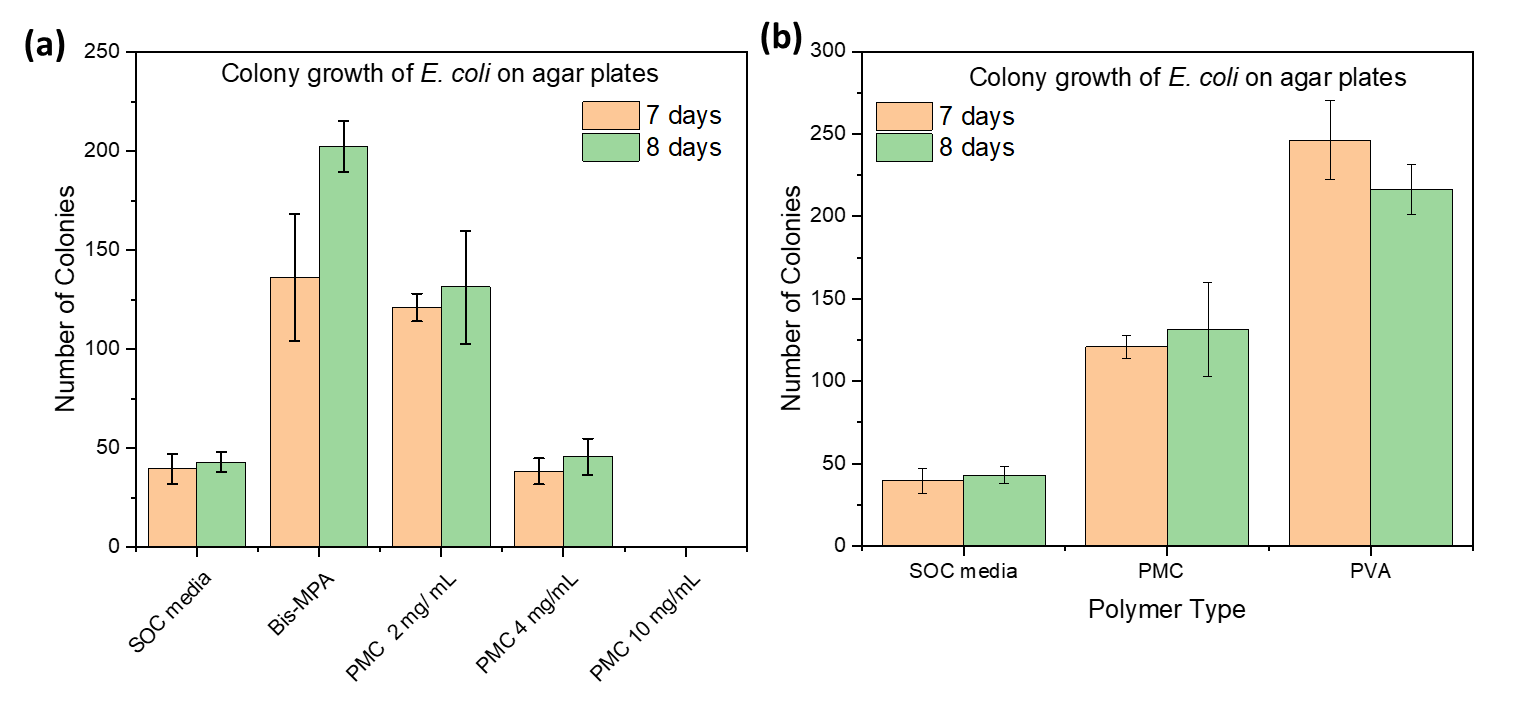


**Figure S24** Comparison of E. coli growth on pure agarose plates (37 °C) comparing **(a)** PMC concentrations and bis-MPA and **(b)** PMC and PVA polymers. Experiments conducted in triplicates.


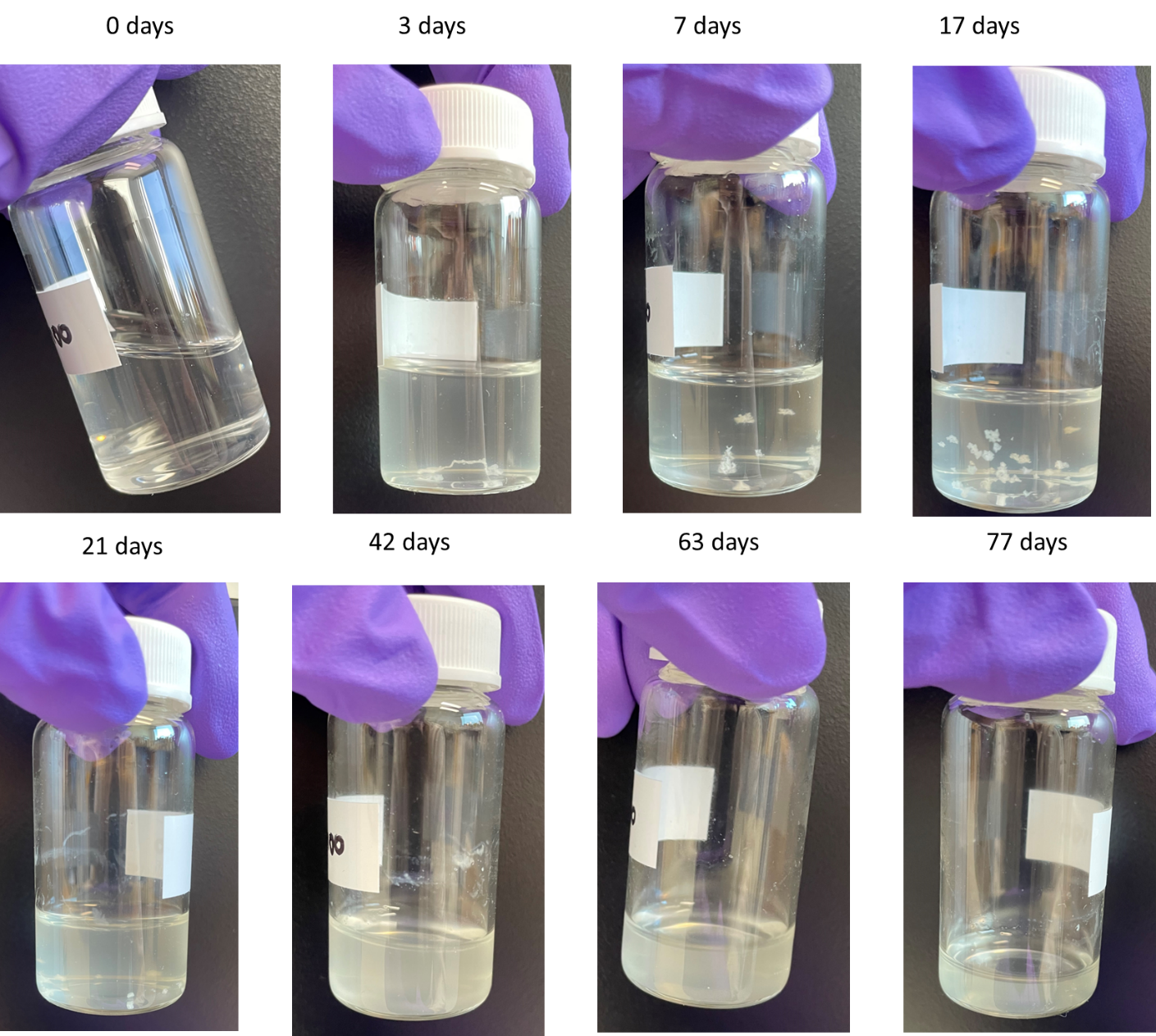


**Figure S25** Pictures of PMC (2 mg·mL^–1^) with E. coli incubated at 37 °C shaken at 180 rpm.


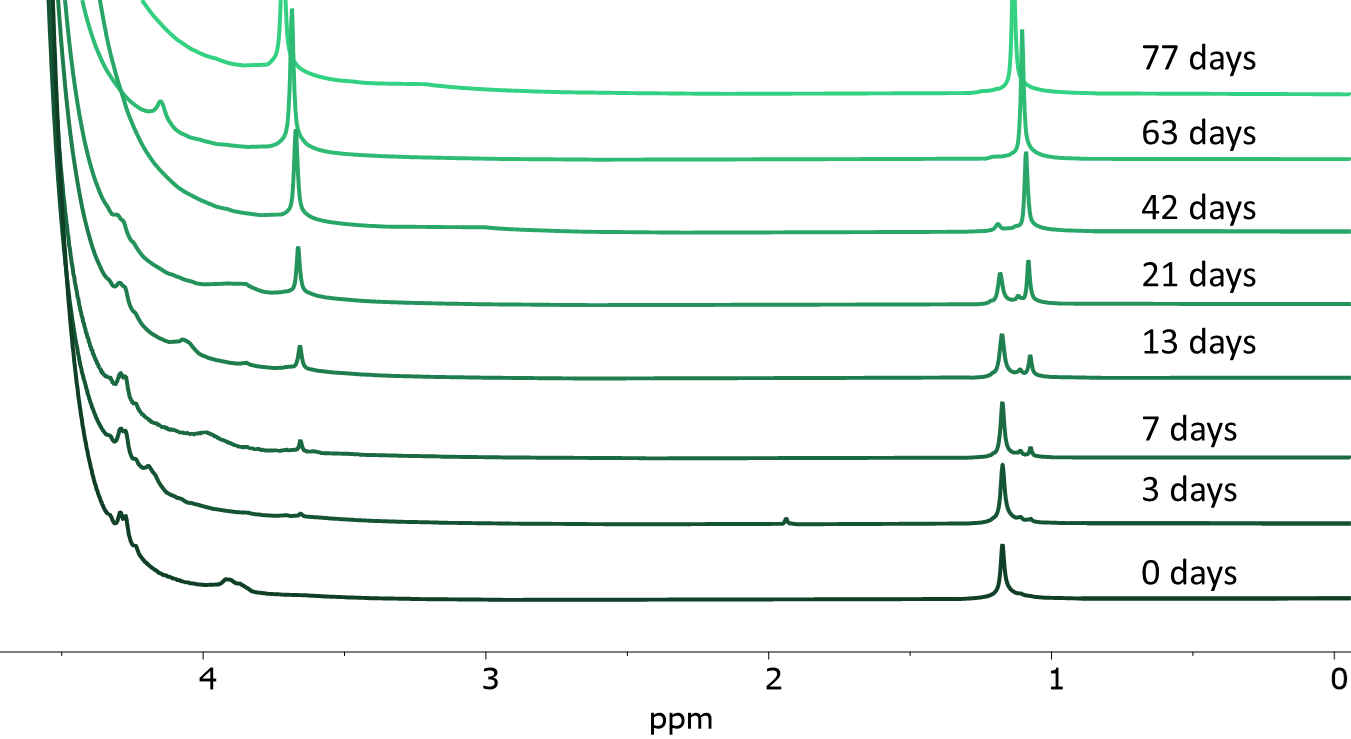


**Figure S26** ^1^H NMR spectra (300 MHz, H_2_O, 298 K) of PMC biodegradation (E. coli, 0.05 M PBS (DI H_2_O), 37 °C, 180 rpm) sample over 77 days.

**Figure S27** Diffusion coefficients of PMC biodegradation (E. coli, 0.05 M PBS (DI H_2_O), 37 °C, 180 rpm) over the degradation period for concentrations 2, 1 and 0.5 mg·mL^–1^ followed by extrapolation to infinite dilution, which was then used for the subsequent calculations.

**Figure S28** Molar mass of bis-MPA (using diffusion NMR spectroscopy) over the biodegradation period (E. coli, 0.05 M PBS (DI H_2_O), 37 °C, 180 rpm).


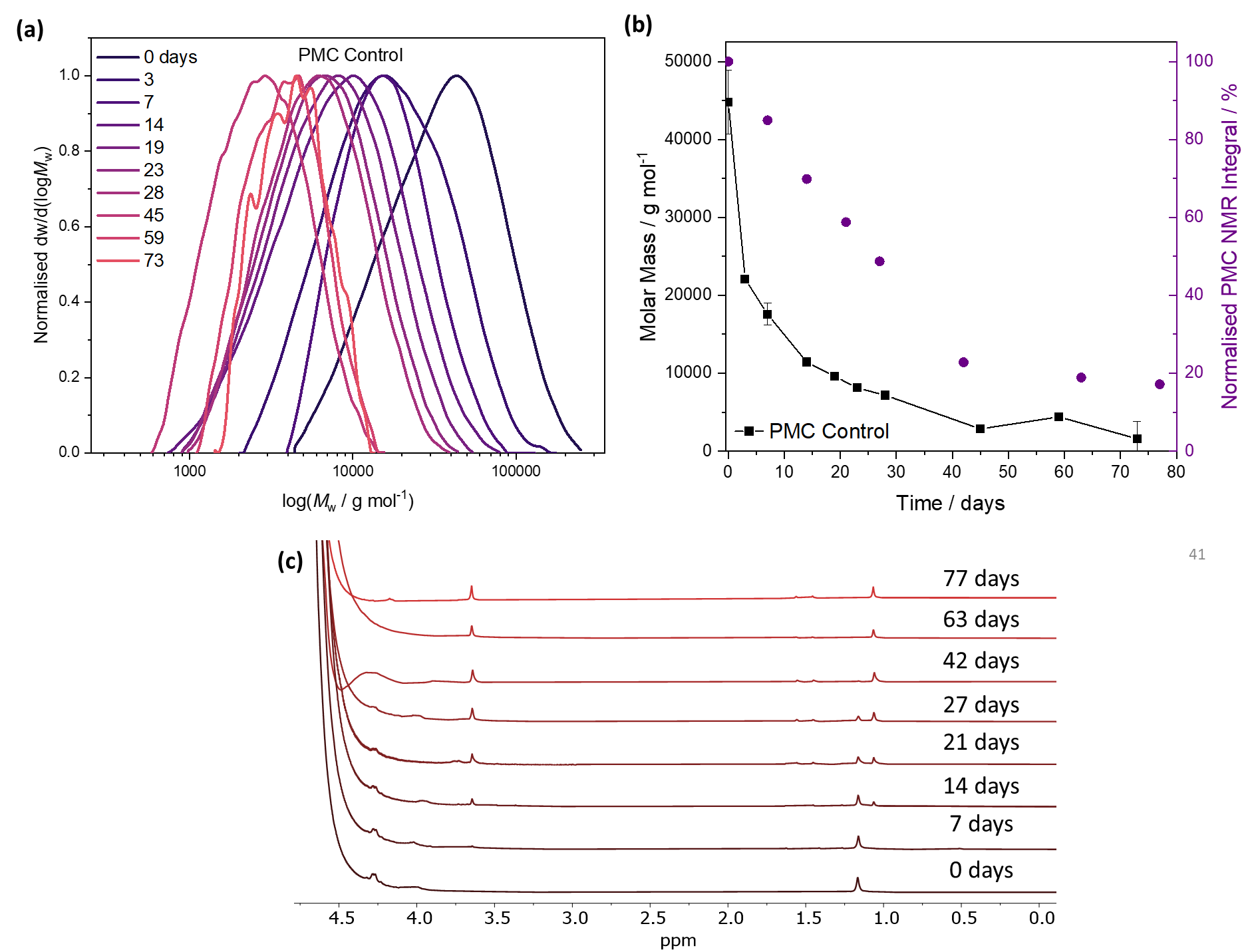


**Figure S29** **(a)** Size exclusion chromatograms of PMC (80:20 H_2_O:MeOH, 0.1 M NaNO_3_, PMC calibrants) without bacteria present (0.05 M PBS DI H_2_O, 37 °C, 180 rpm, 0.083 equiv. ampicillin sodium salt) **(b)** mass loss (black line) and NMR integrals (purple dots) of PMC signal plotted against time for PMC biodegradation in the same conditions. **(c)** ^1^H NMR spectra (300 MHz, H_2_O, 298 K) of PMC degradation without bacteria present over 77 days. Peaks at δ= 1.06 and 3.64 ppm correspond to bis-MPA, peaks at δ= 1.16 and 4.21 correspond to PMC and peaks between δ= 1.40 -1.56 ppm correspond to the ampicillin sodium salt.


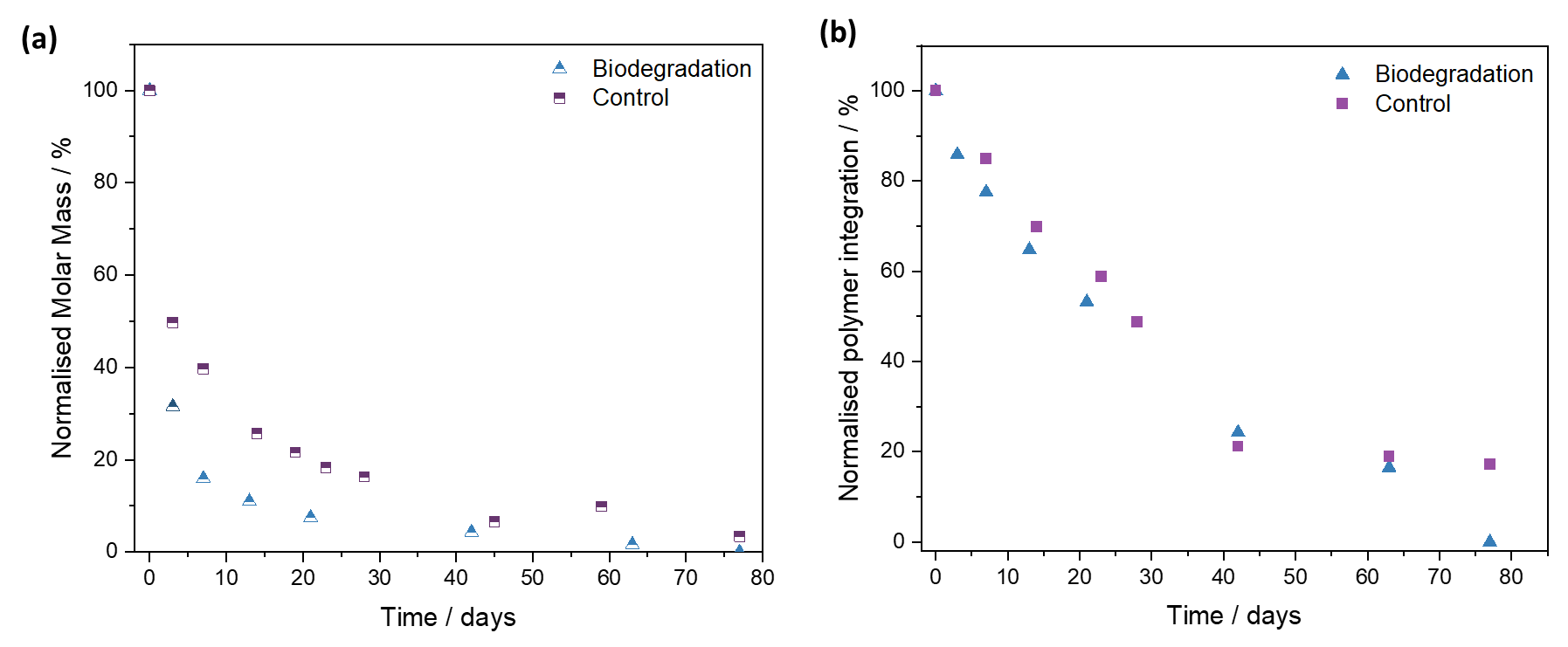


**Figure S30** **(a)** Normalised molar mass plotted against time of PMC in the presence of E. coli (biodegradation) and without (control) containing 0.083 equiv. ampicillin sodium salt in 0.05 M PBS (DI H_2_O) at 37 °C, 180 rpm. **(b)** Normalised absolute integral of PMC in presence of E. coli (biodegradation with E. coli) and without (named control) containing 0.083 equiv. ampicillin sodium salt in 0.05 M PBS (DI H_2_O) at 37 °C, 180 rpm.


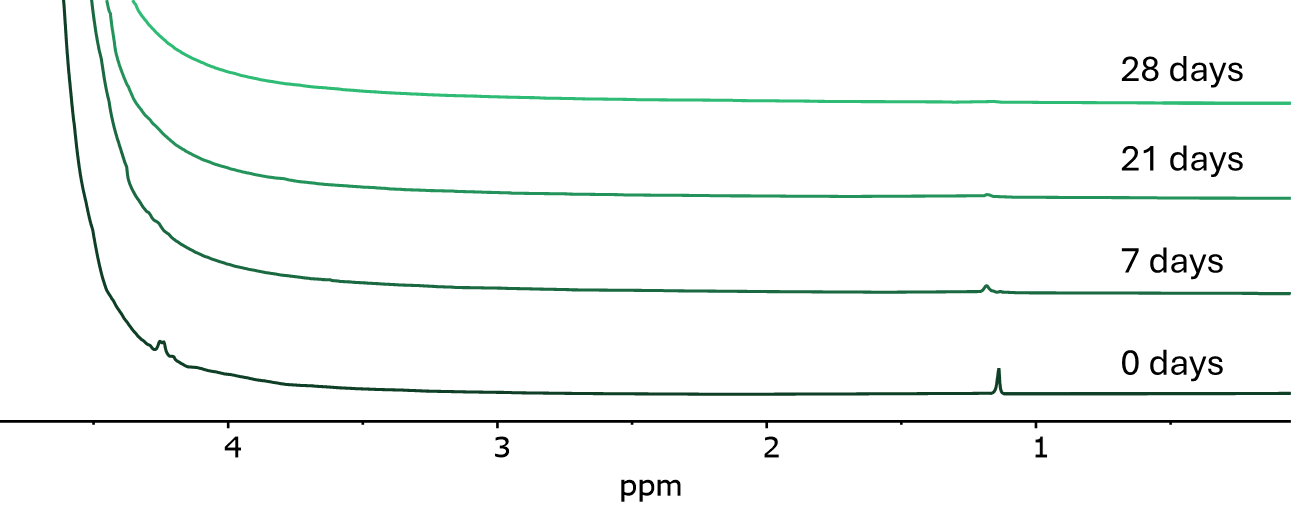


**Figure S31** ^1^H NMR spectra (300 MHz, H_2_O, 298 K) over 28 days for PMC biodegradation (2 mg·mL^–1^, E. coli, 0.05 M PBS (DI H_2_O)) in a dialysis bag at 37 °C, 180 rpm.


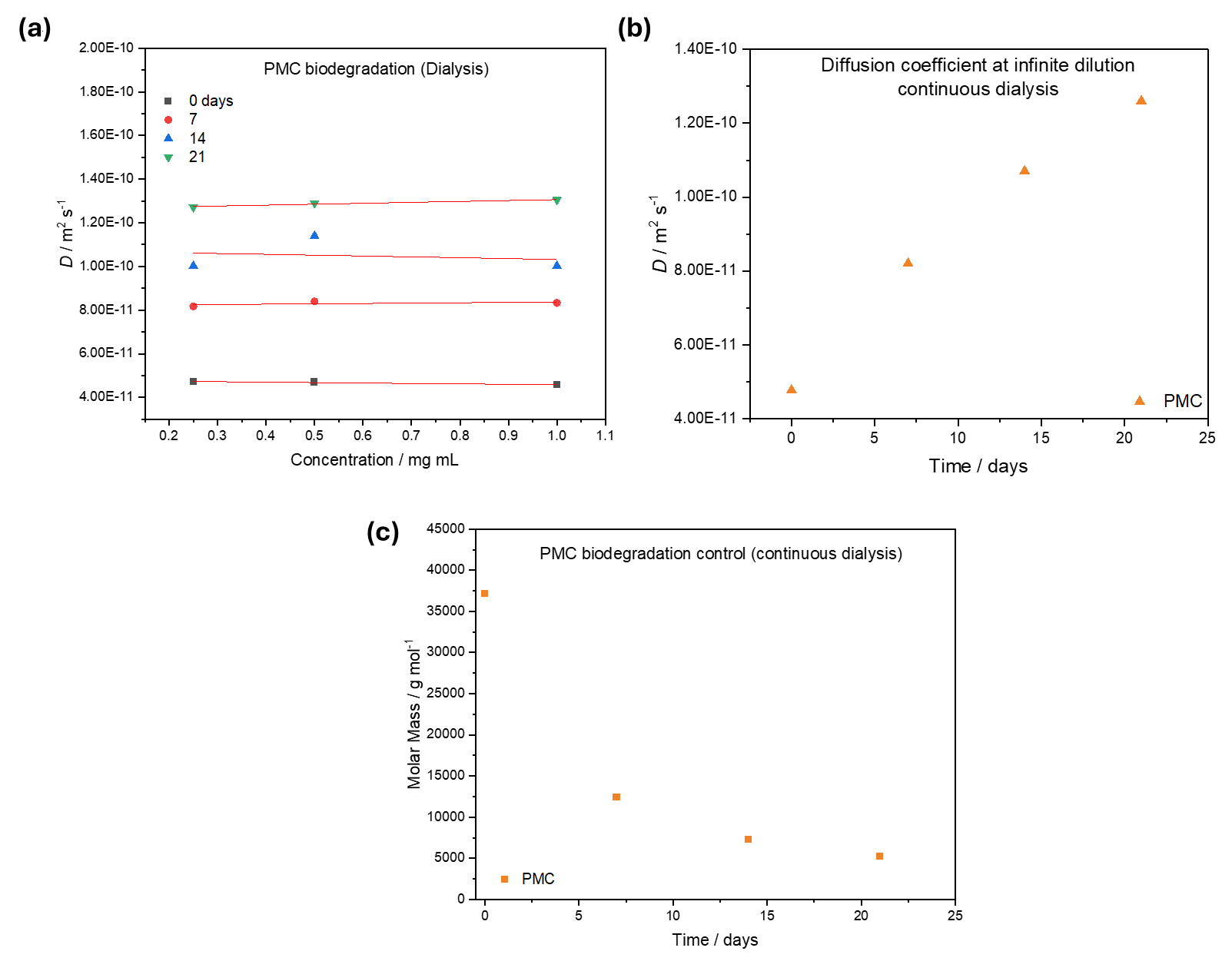


**Figure S32 (a)** Diffusion coefficients of PMC within the dialysis bag (E. coli, 0.05 M PBS (DI H_2_O), 37 °C, 180 rpm) over the 22 day degradation period at concentrations of 2, 1 and 0.5 mg·mL^–1^ followed by extrapolation to infinite dilution to afford the corrected D. **(b)** The corrected D at infinite dilution, which was then used for the subsequent calculations. **(c)** Final molar mass results for PMC biodegradation with continuous removal of degradation products under same conditions.


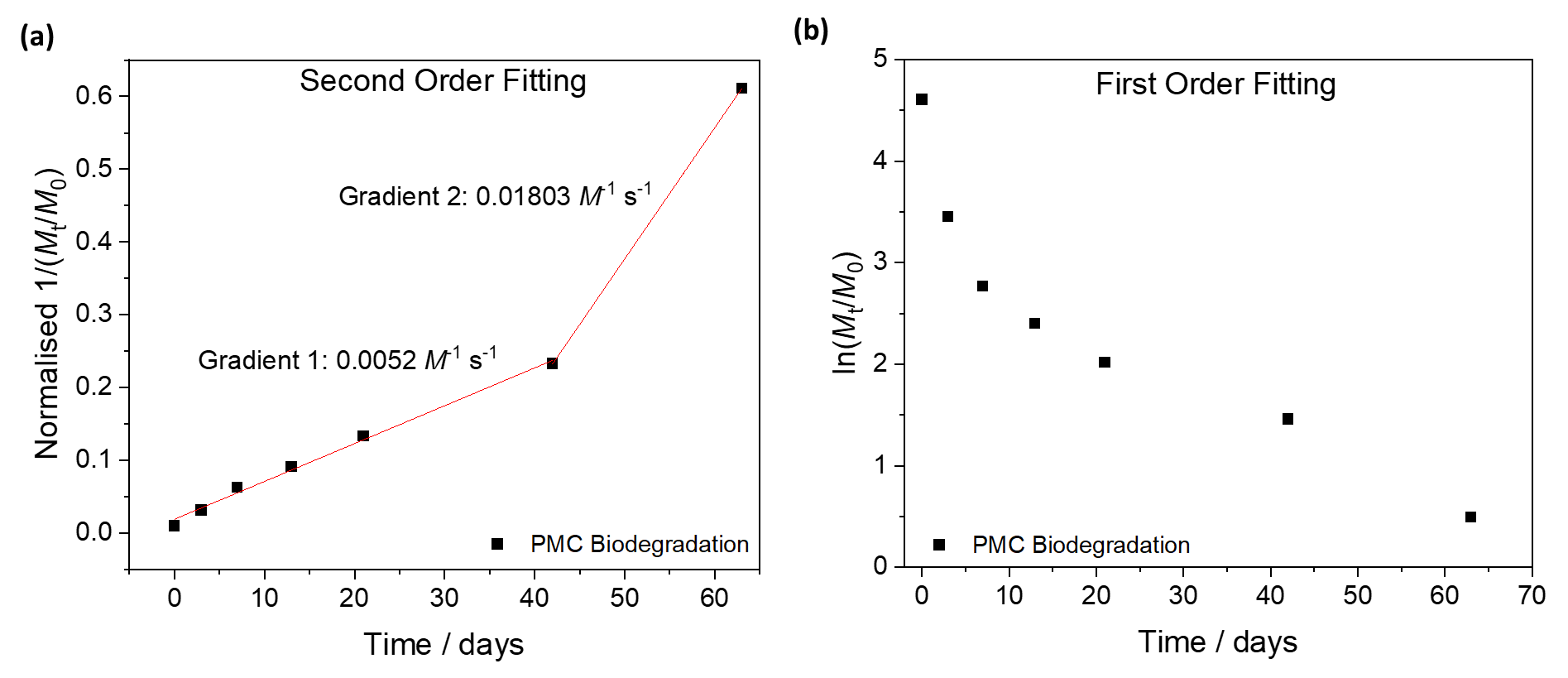


**Figure S33** **(a)** Second order rate kinetics of PMC biodegradation (E. coli, 0.05 M PBS (DI H_2_O), 37 °C, 180 rpm) **(b)** First order fitting of PMC biodegradation (E. coli, 0.05 M PBS (DI H_2_O), 37 °C, 180 rpm).

**Figure S34** Comparison of rate constants from PMC degradation with and without bacteria (0.05 M PBS (DI H_2_O), 37 °C, 180 rpm).


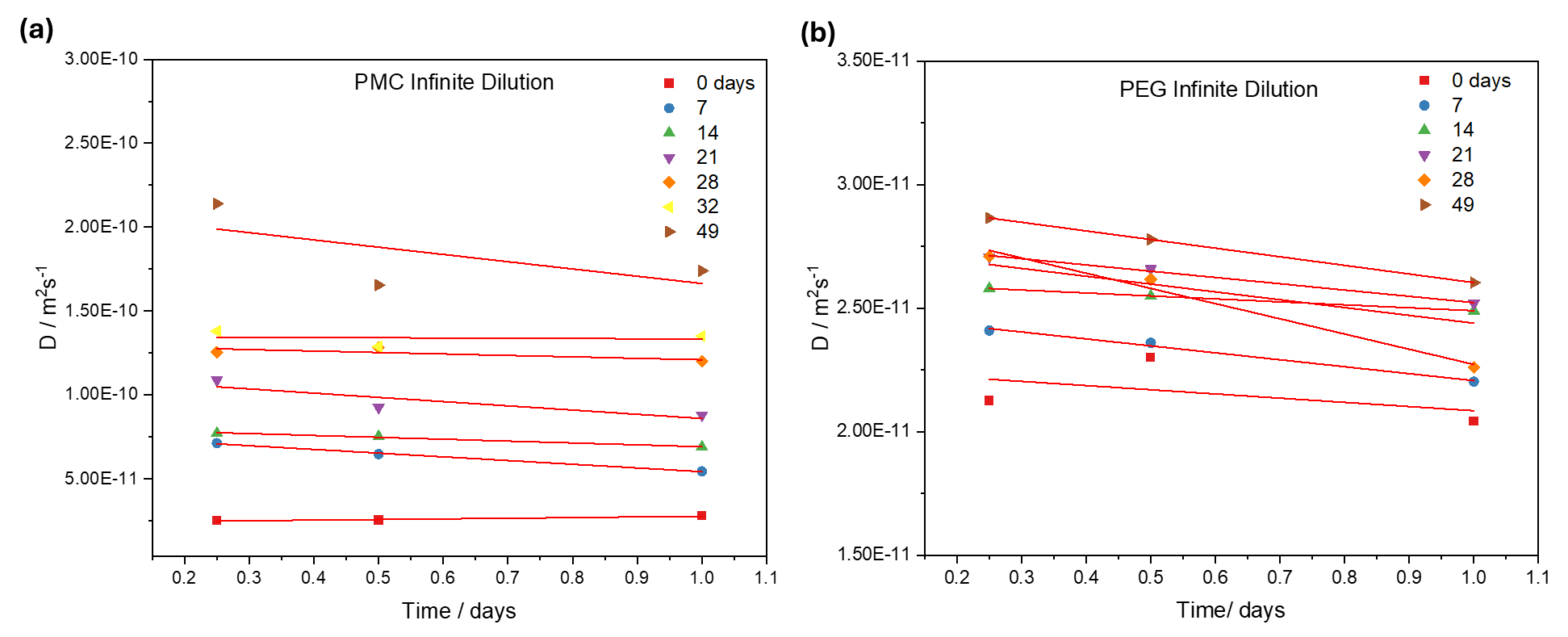


**Figure S35** Diffusion coefficients of **(a)** PMC and **(b)** PEG over the degradation period (E. coli, 0.05 M PBS (DI H_2_O), 37 °C, 180 rpm) at concentrations of 2, 1 and 0.5 mg·mL^–1^ followed by extrapolation to infinite dilution, which was then used for the subsequent calculations.


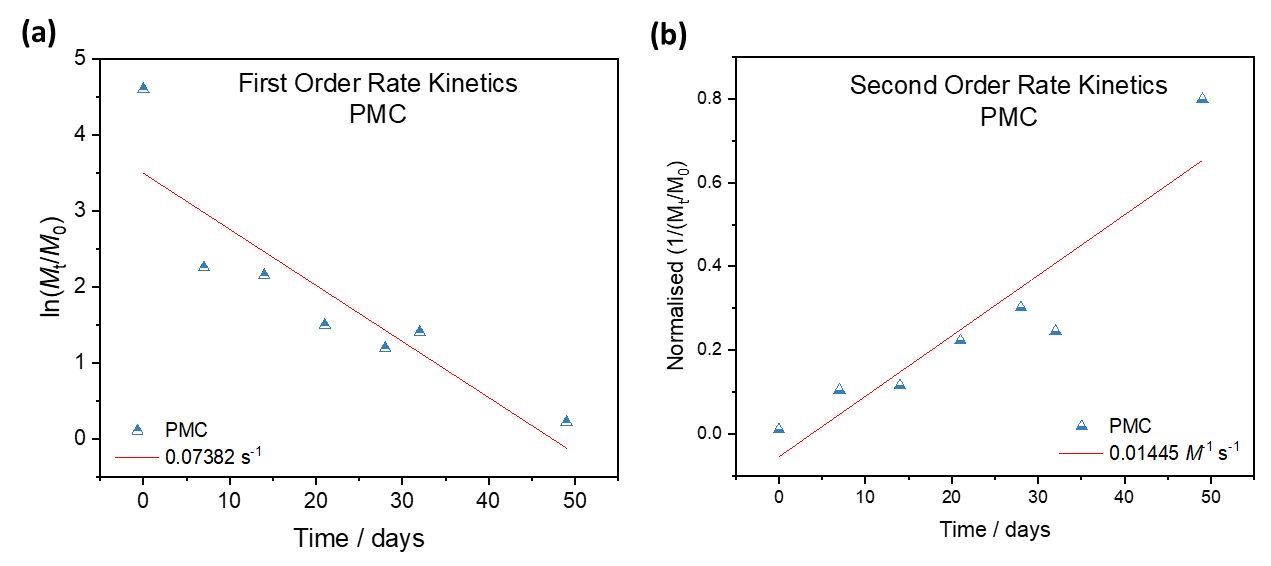


**Figure S36 (a)** First order rate fitting of PMC in the presence of E. coli and PEG (E. coli, 0.05 M PBS (DI H_2_O), 37 °C, 180 rpm) **(b)** Second order rate fitting of the same condition.

**Figure S37** Normalised second order plotted against time for PMC and PEG (E. coli, 0.05 M PBS (DI H_2_O), 37 °C, 180 rpm).

**Figure S38** Absolute peak intensity of PEG plotted against time during biodegradation (E. coli, 0.05 M PBS (DI H_2_O), 37 °C, 180 rpm).


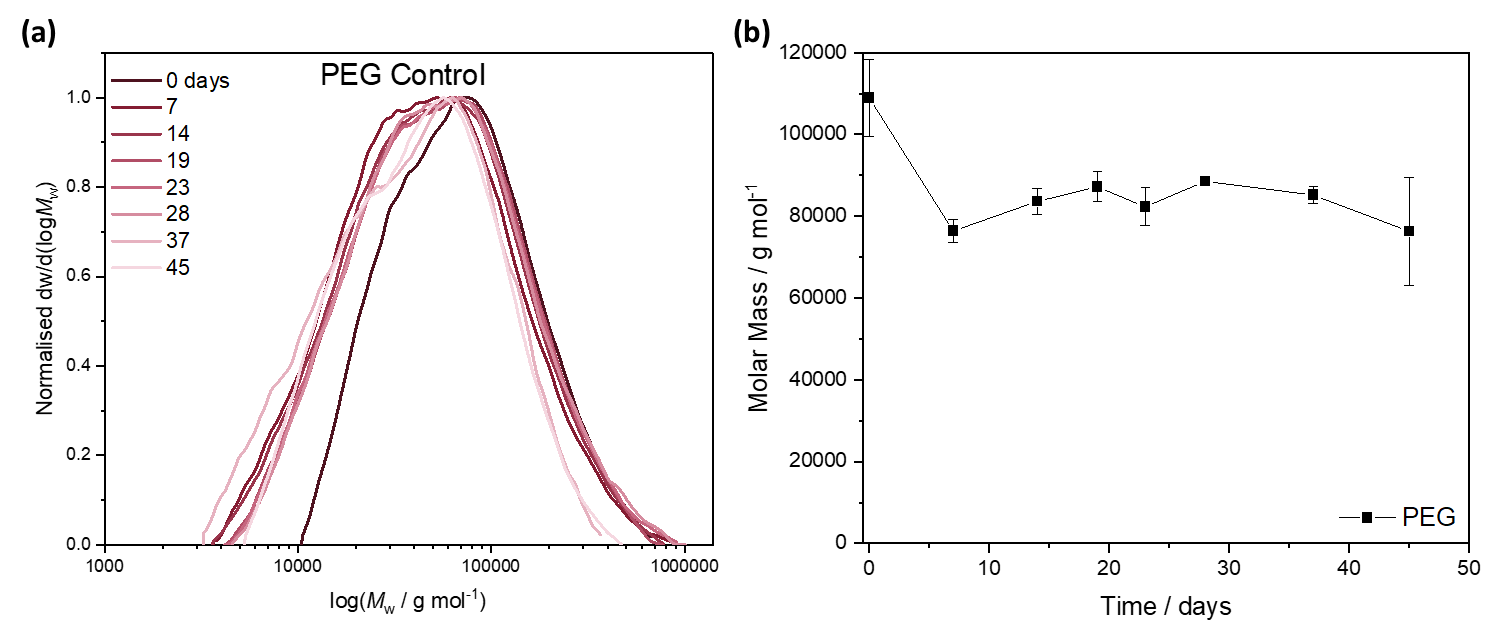


**Figure S39 (a)** Size exclusion chromatogram of PEG during control experiment (no bacteria) (0.05 M PBS (DI H_2_O), 0.083 equiv. ampicillin sodium salt, 37 °C, 180 rpm) **(b)** Molar mass of PEG during control (no bacteria).

**Figure S40** Comparison of molar mass of PEG from biodegradation (with E. coli, 0.05 M PBS (DI H_2_O), 37 °C and 180 rpm) and control experiments (0.083 equiv. ampicillin sodium salt, 0.05 M PBS (DI H_2_O), 37 °C and 180 rpm), both incubated at 37 C at 180 rpm.

# **Diffusion NMR spectroscopy signal decays**

Diffusion coefficients were calculated using the S-T equation using the peak signal decays as described previously. Signal decay graphs of all diffusion experiments are shown below.

**PMBC Calibrants**

Signal Decays of PMBC with different degrees of polymerisation of (a) 15 (b) 40 (c) 75 (d) 240 (e) 680.


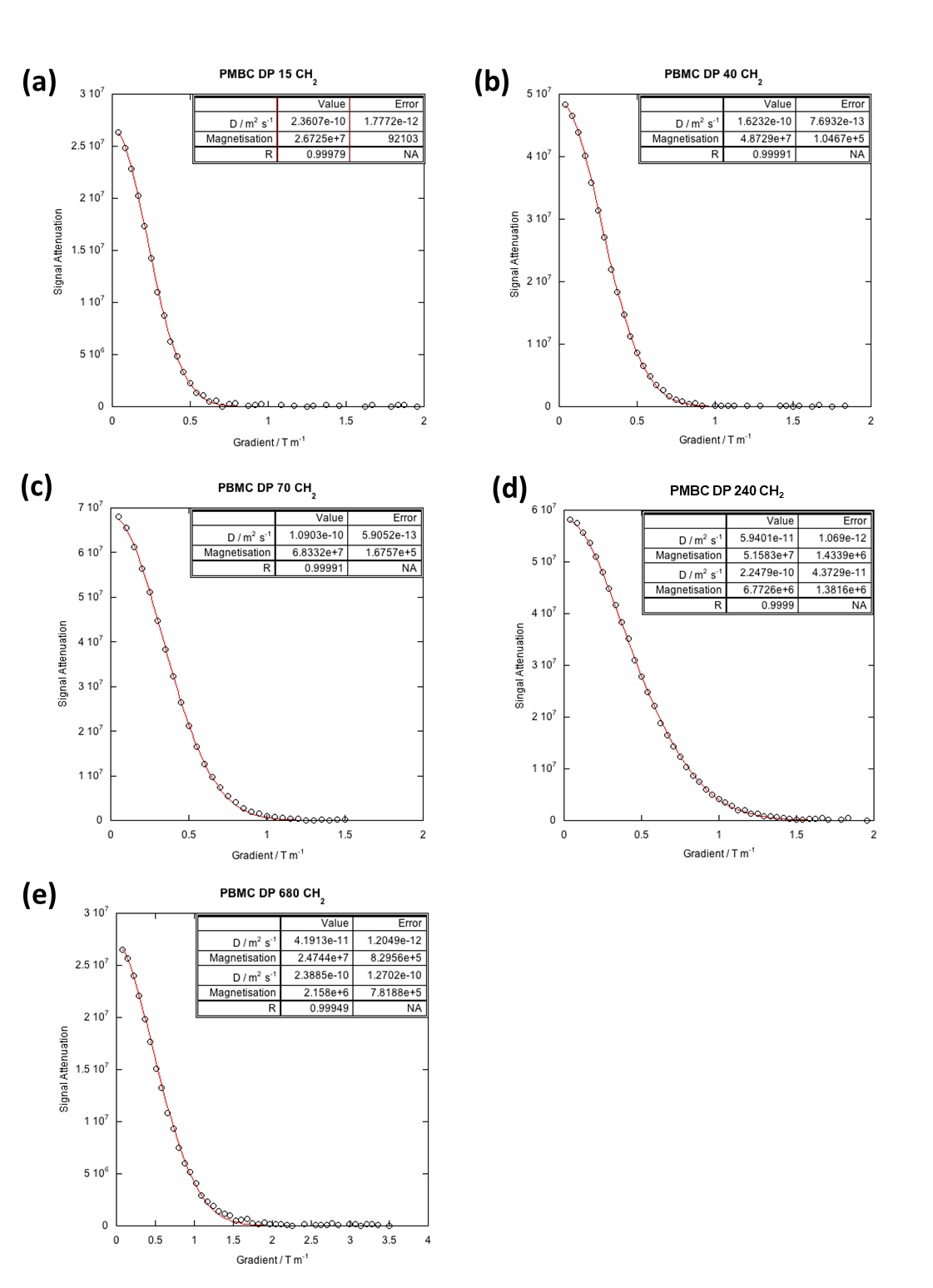


**PMC Calibrants**

Signal decays of PMC with different degrees of polymerisation of (a) 15 (b) 40 (c) 75 (d) 240 (e) 680.


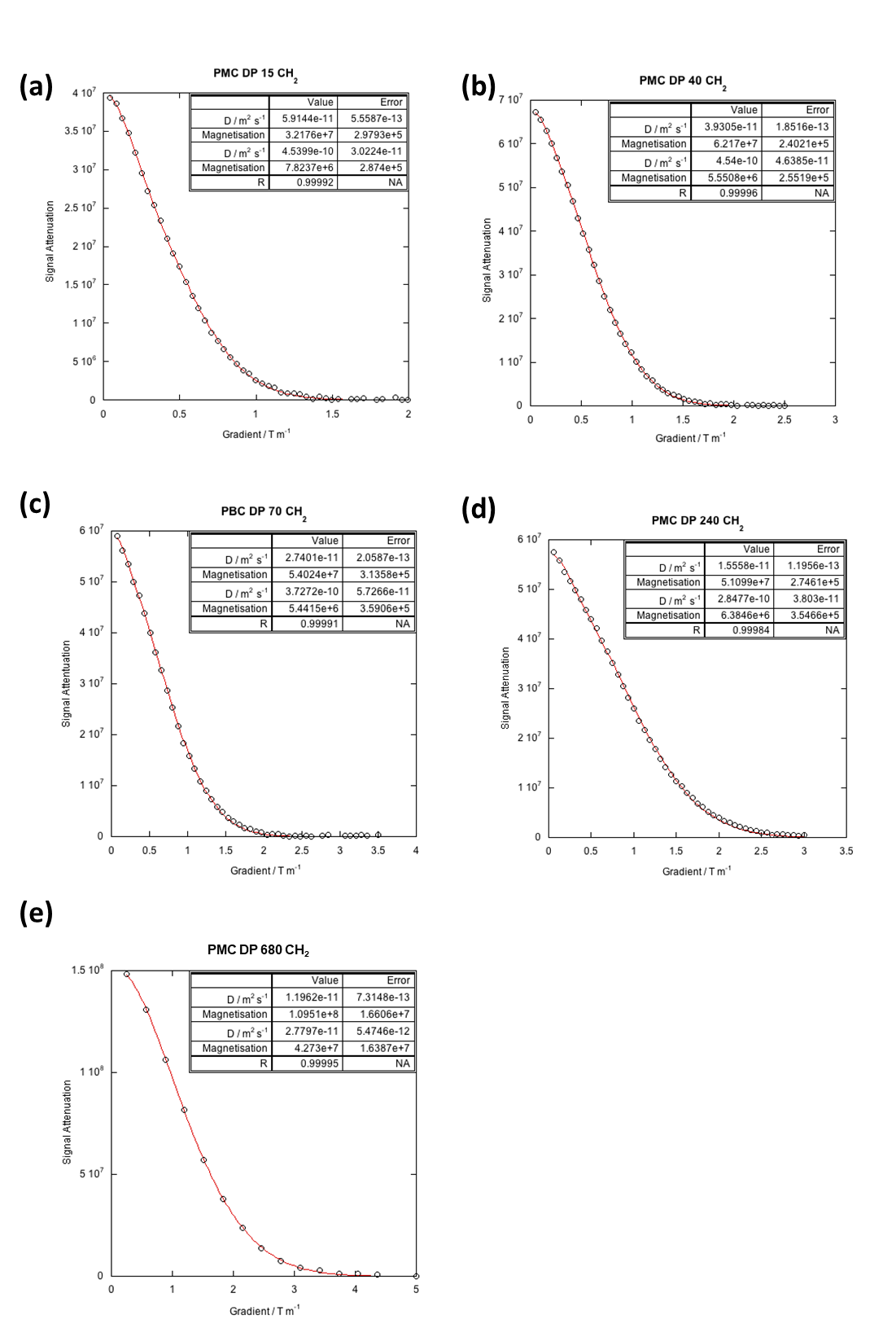


**PMC Hydrolysis (pH 9.1(D_2_O) 19 °C)**

Signal decays for each time point and concentration with (a) 1 mg·mL^–1^, (b) 0.5 mg·mL^–1^ (c) 0.125 mg·mL^–1^ for time points 0 and 1 days, and 0.25 mg·mL^–1^ for the remaining time points, (d) degradation product using 1 mg·mL^–1^ sample.

**
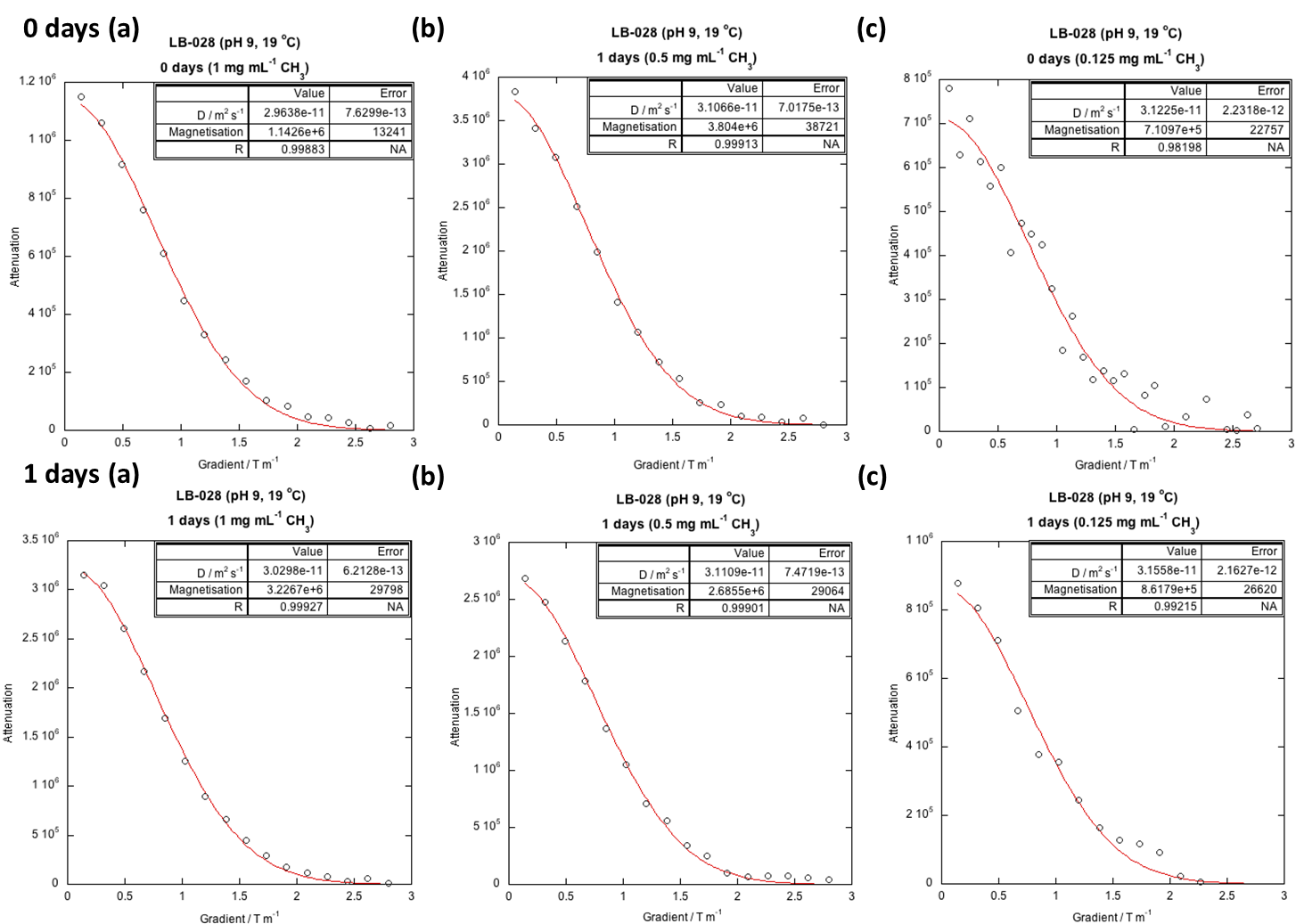

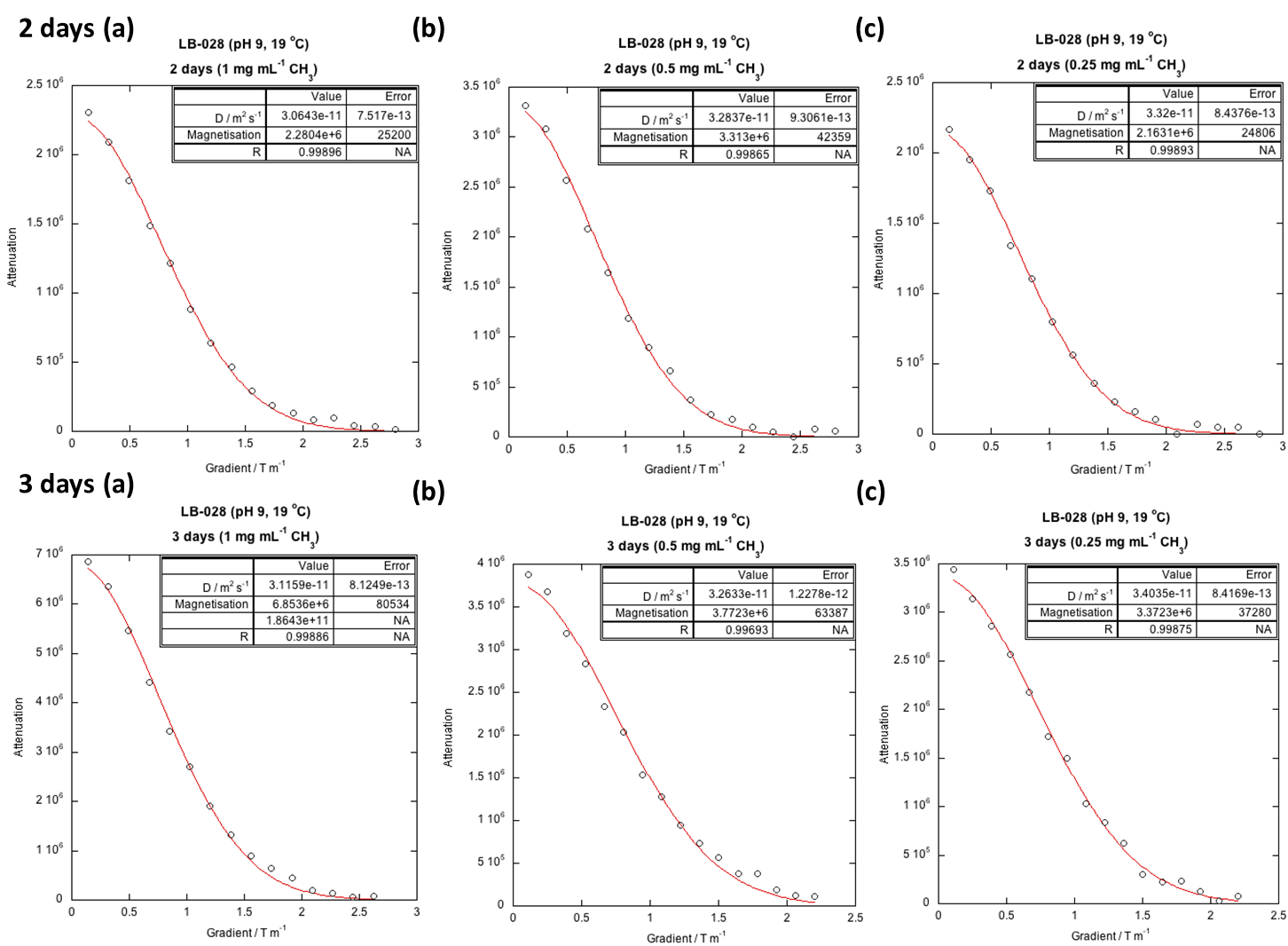
**

**
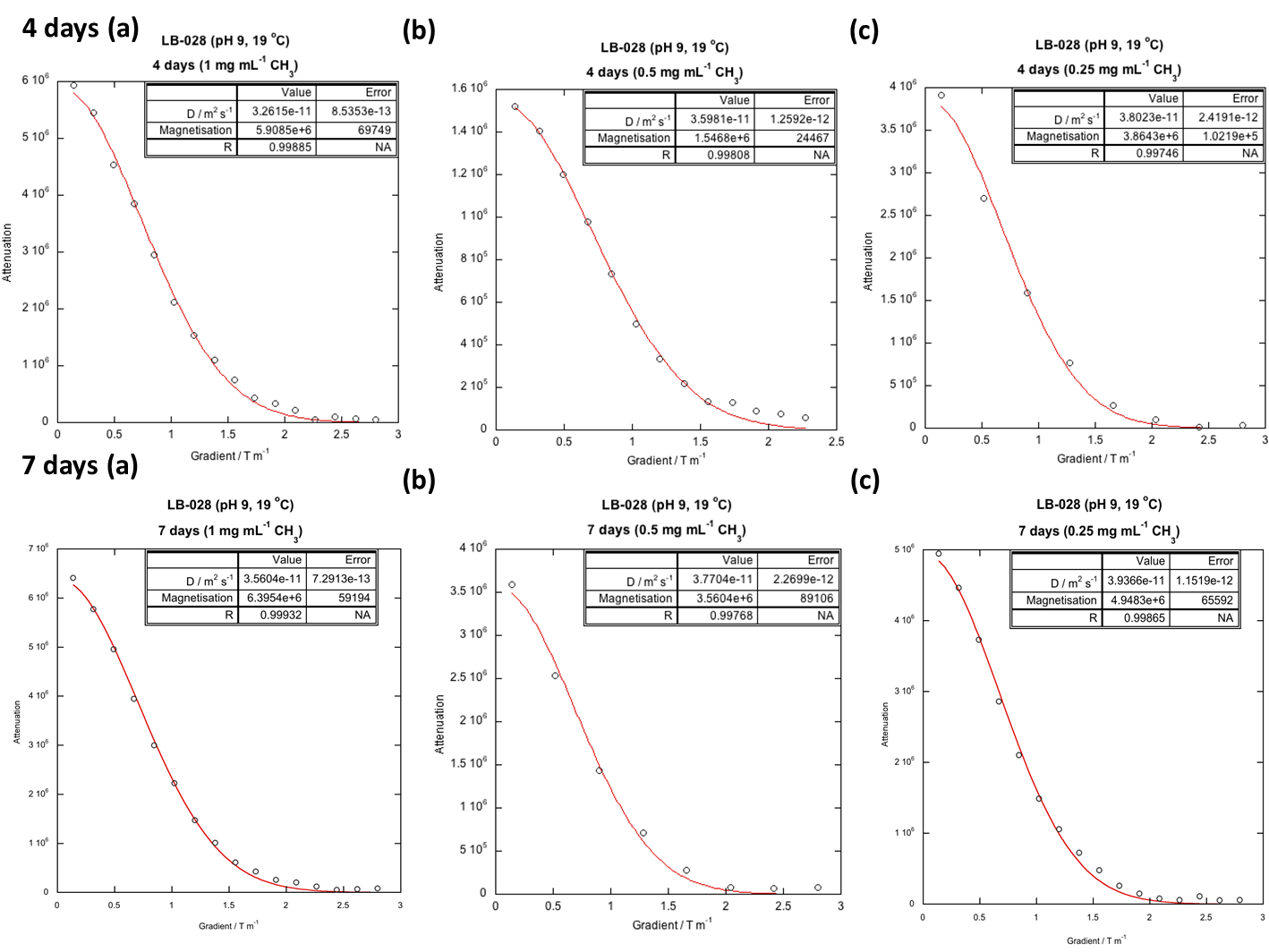

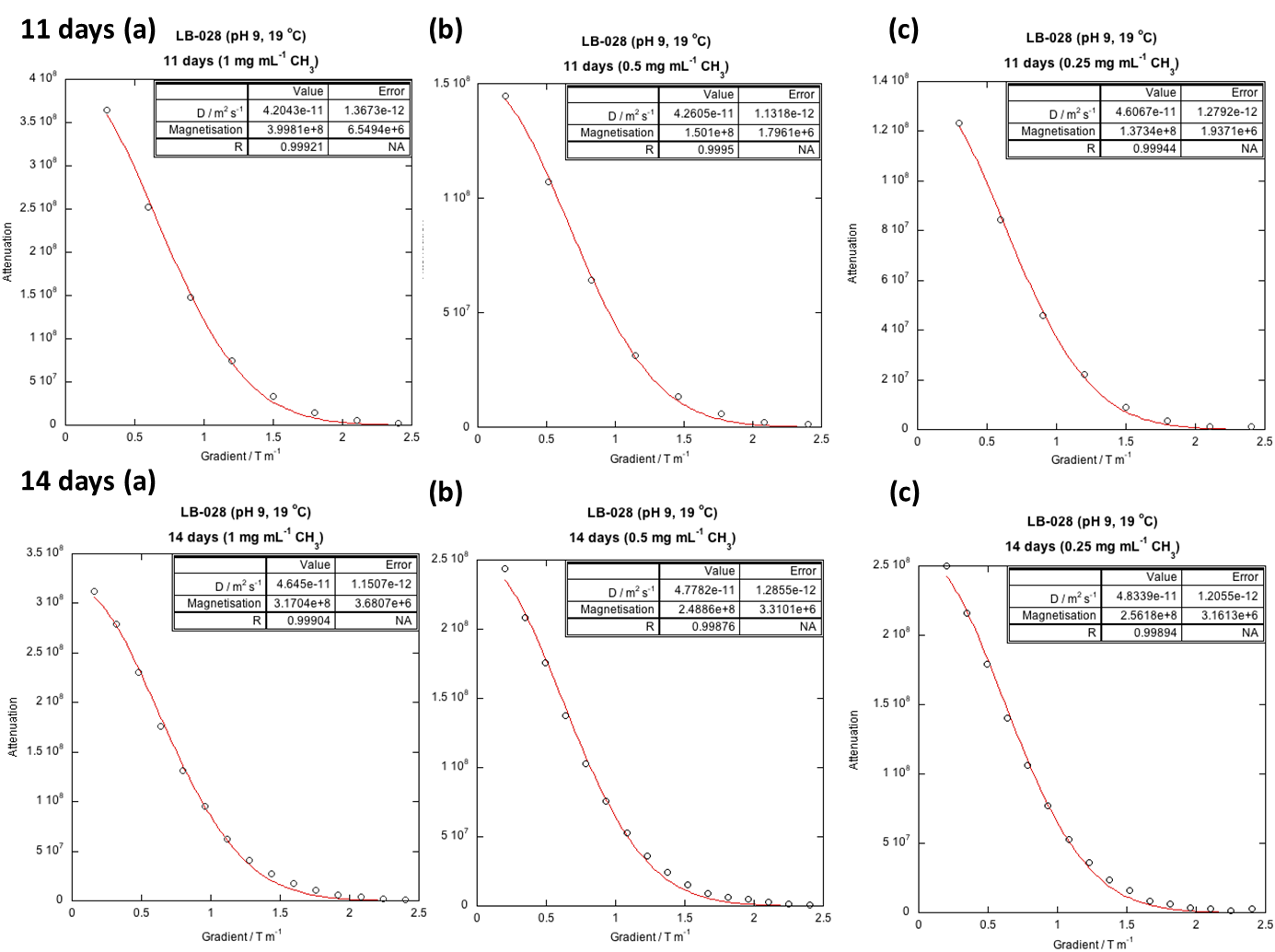
**

**
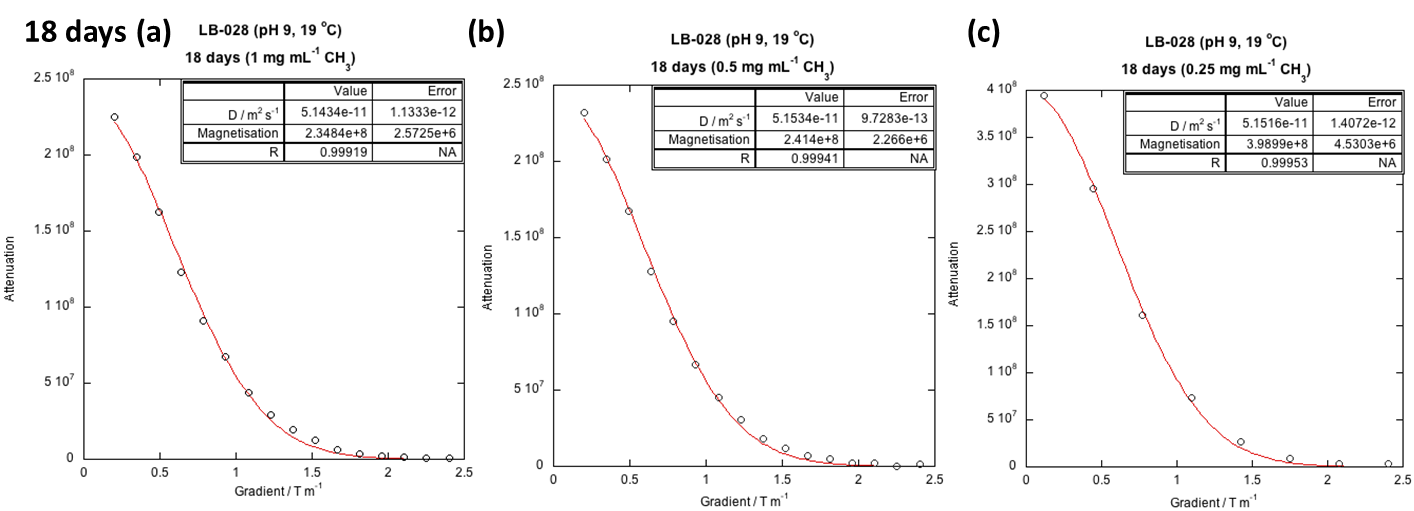
**

**
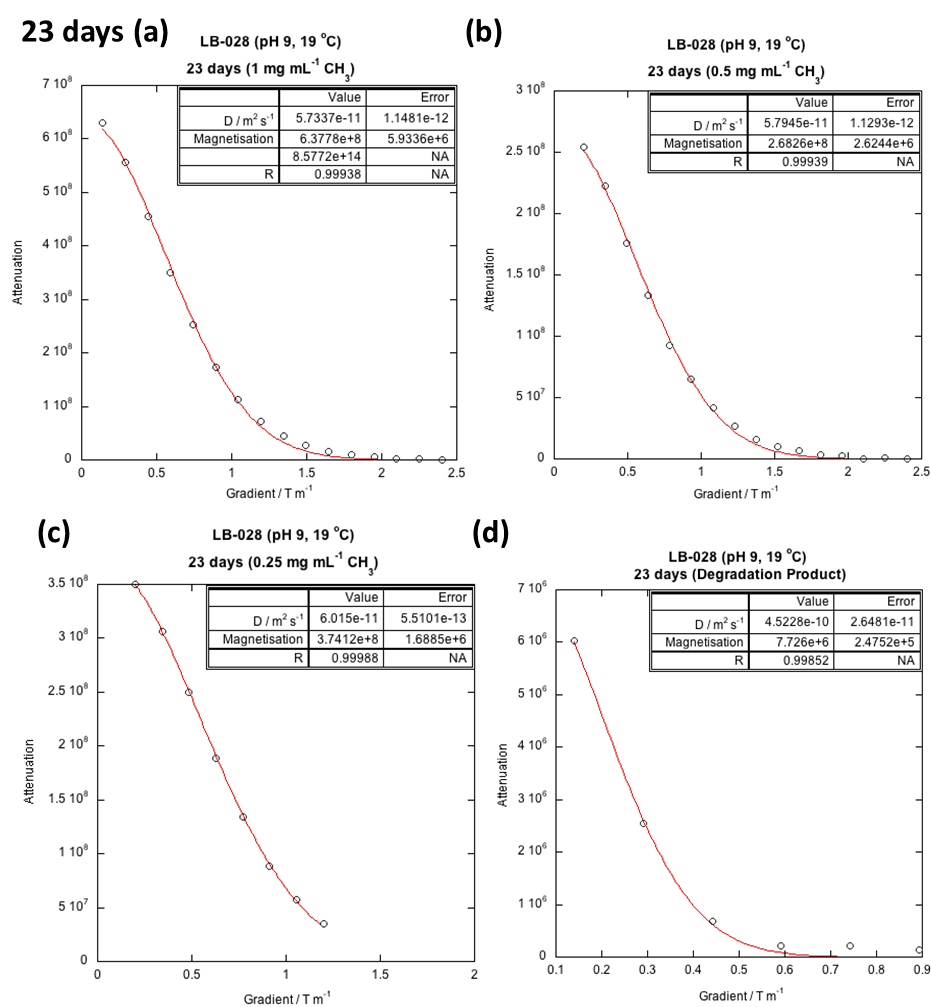
**

**
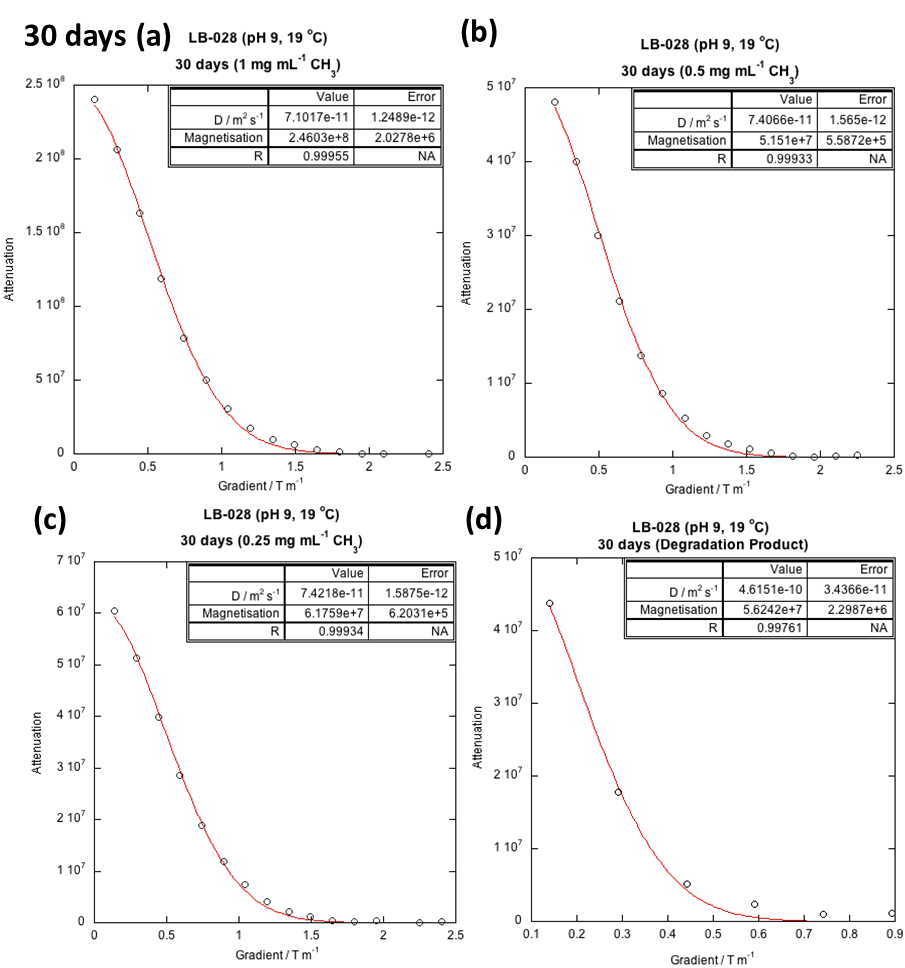
**

**
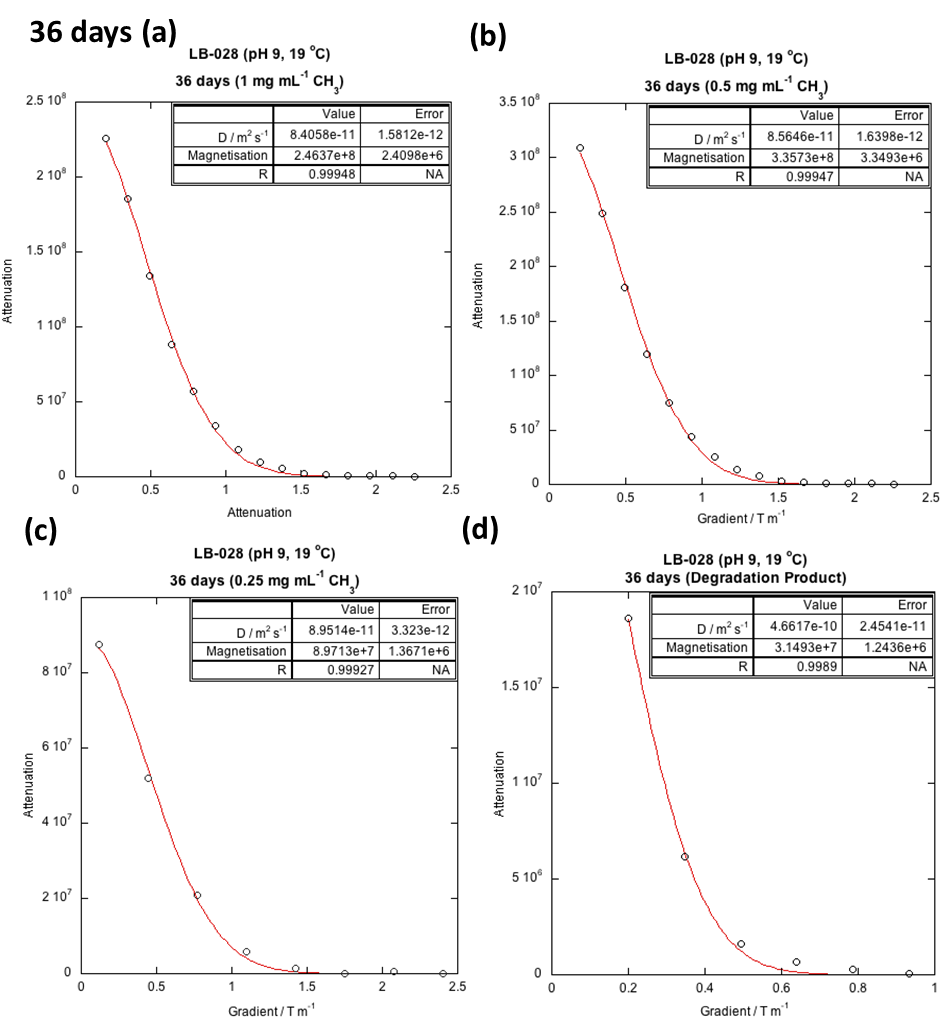
**

**
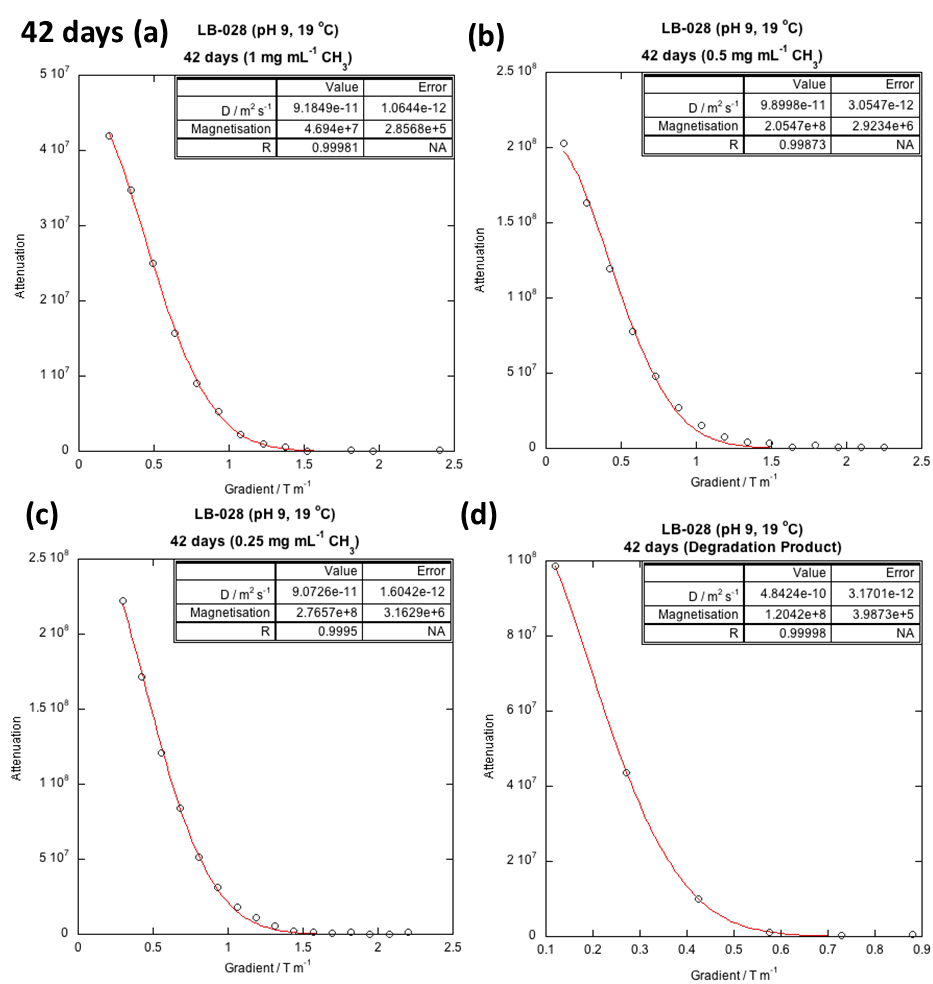
**

**
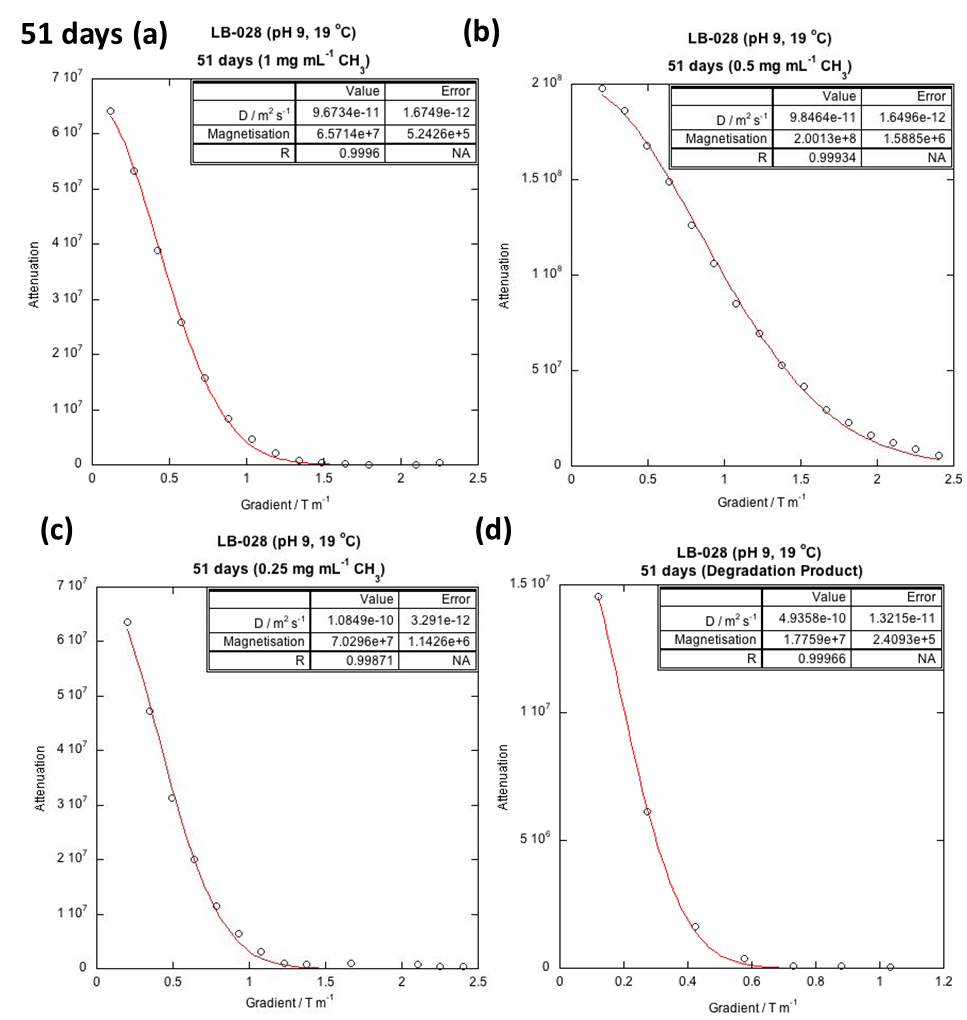
**

**
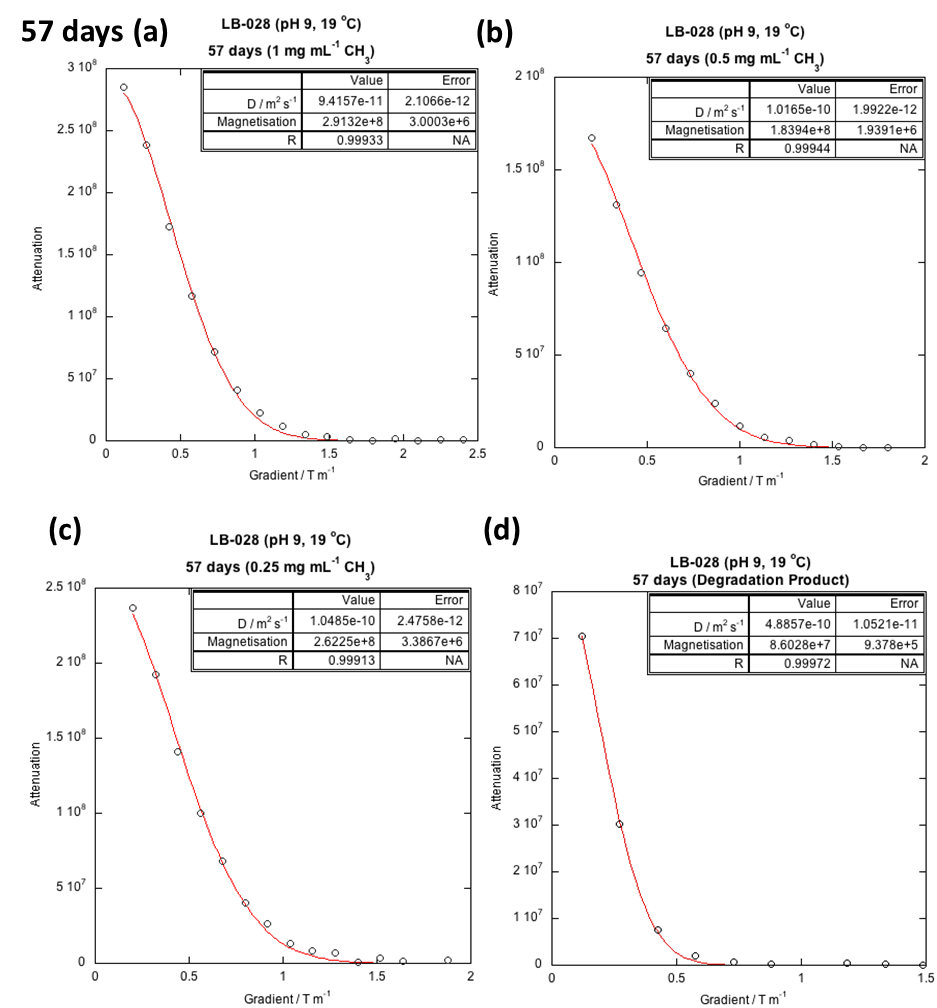
**

**
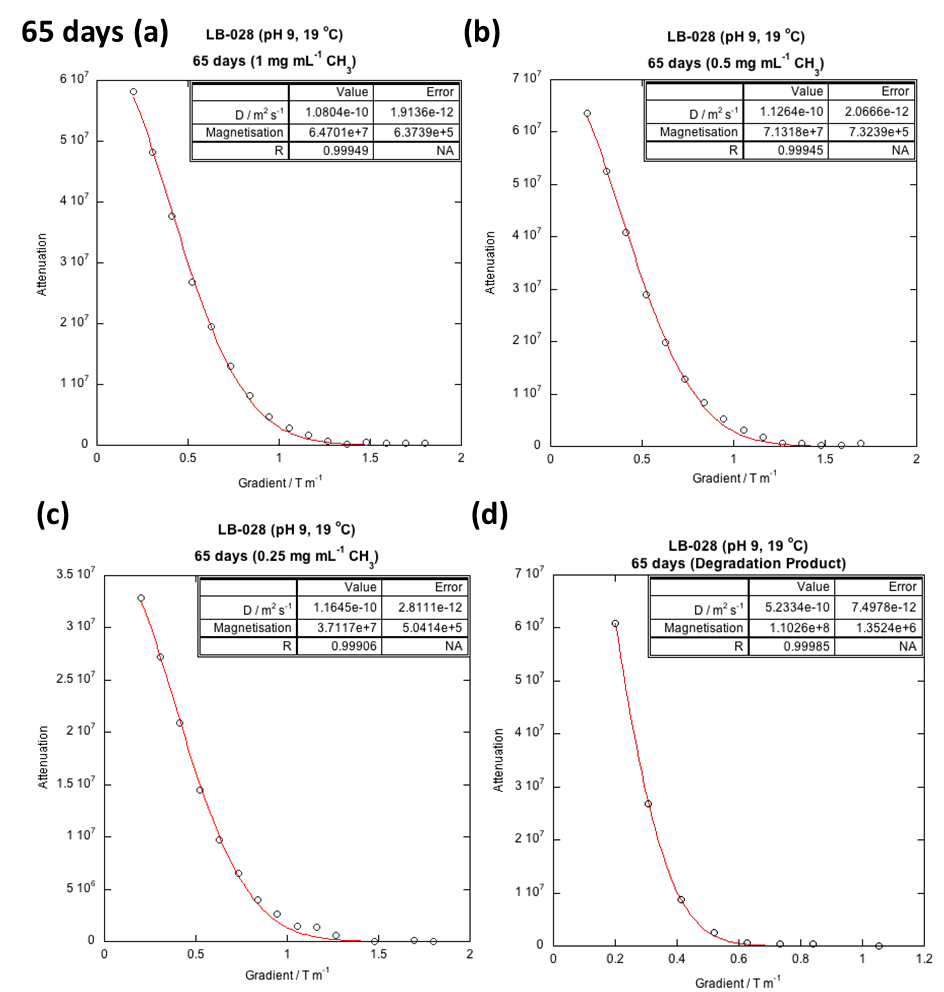
**

**PMC biodegradation 1 (*E. coli*, 0.05 M PBS (DI H2O), 37 °C, 180 rpm)**

Signal intensity decays for PMC biodegradation at (a) 2 mg mL^–1^ (b) 1 mg mL^–1^ (c) 0.5 mg mL^–1^ and (d) Bis-MPA at 2 mg mL^–1^.

**
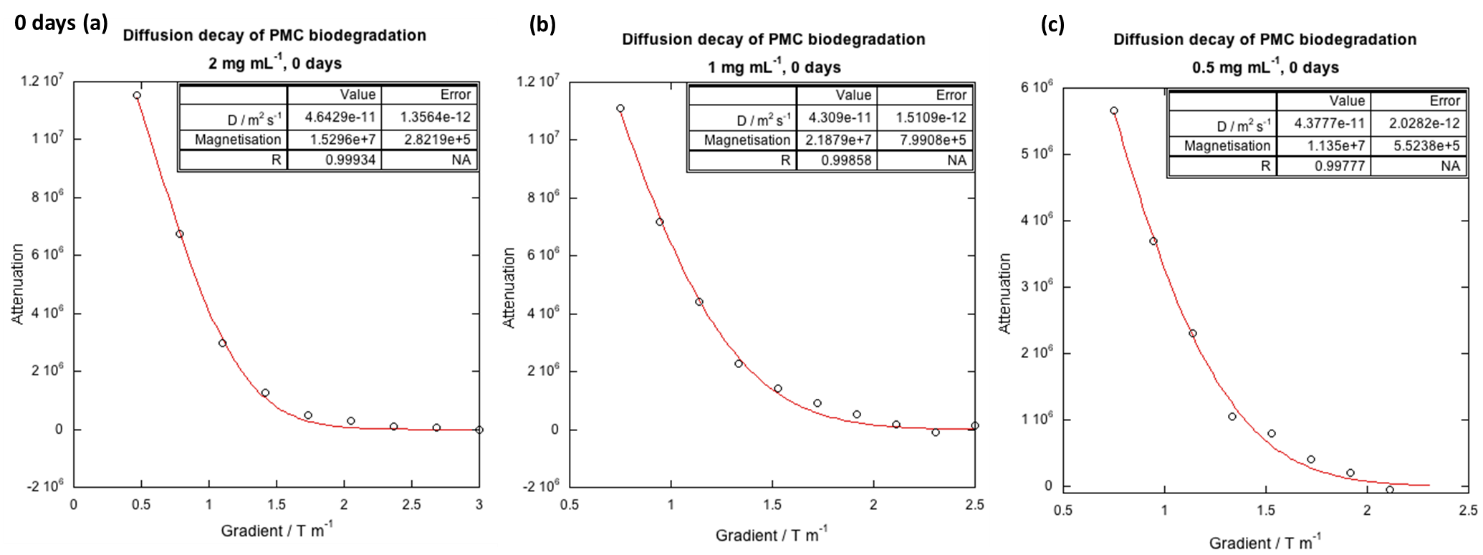
**

**
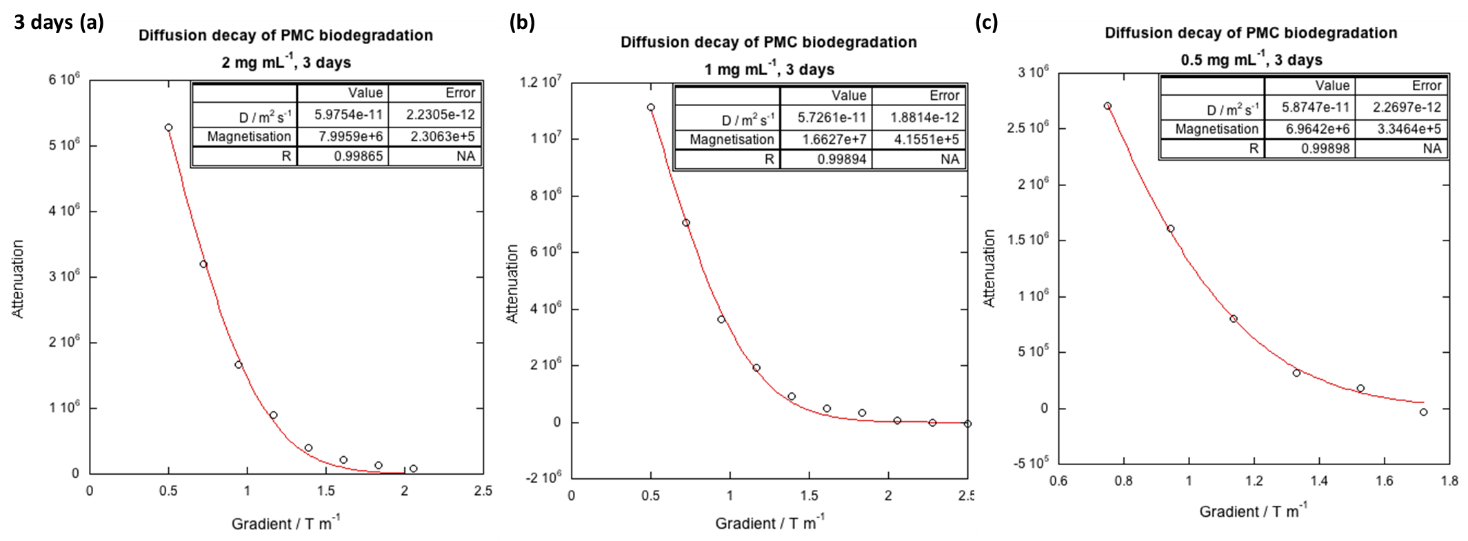
**

**
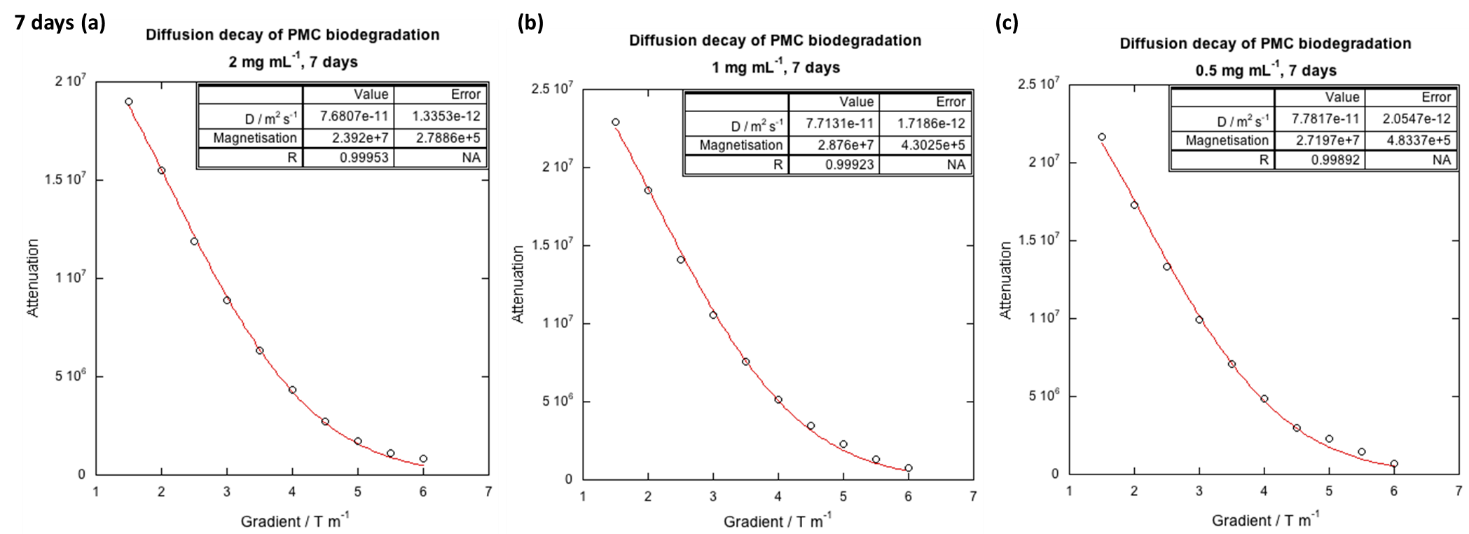
**

**
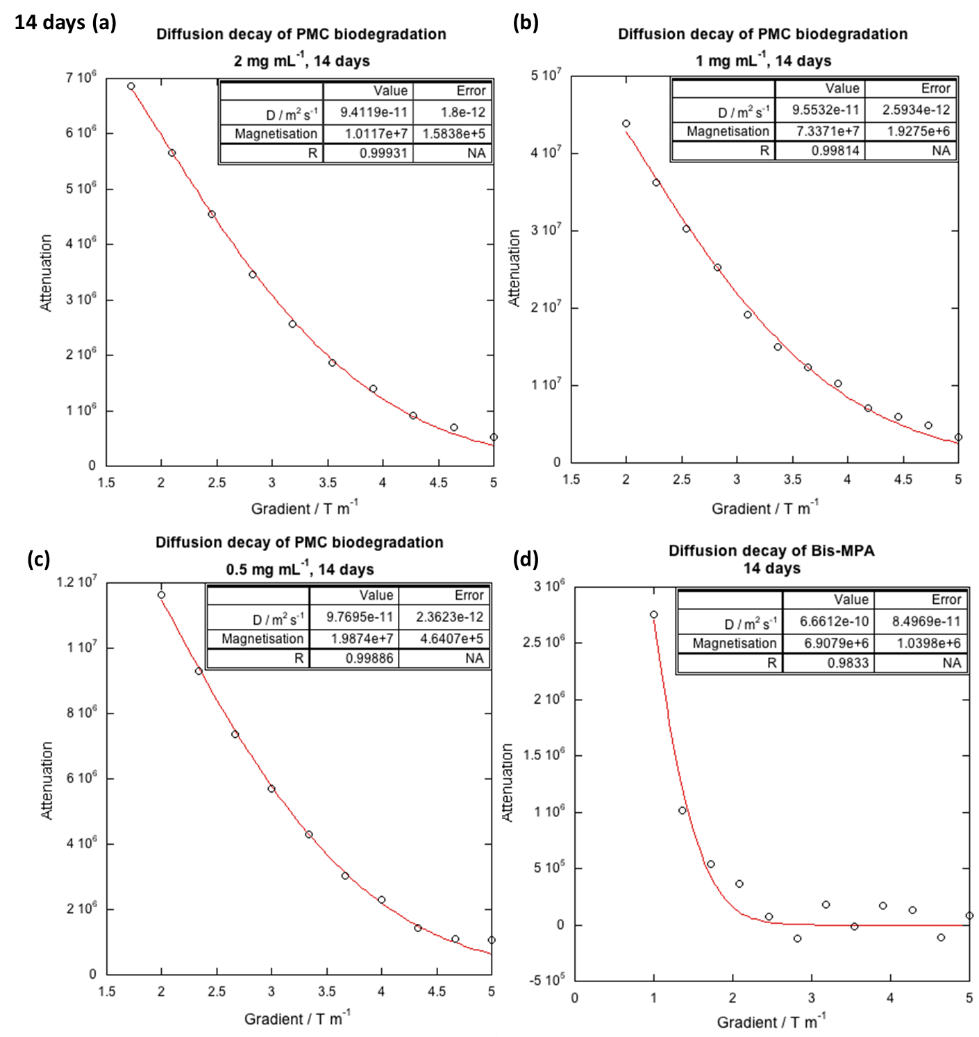

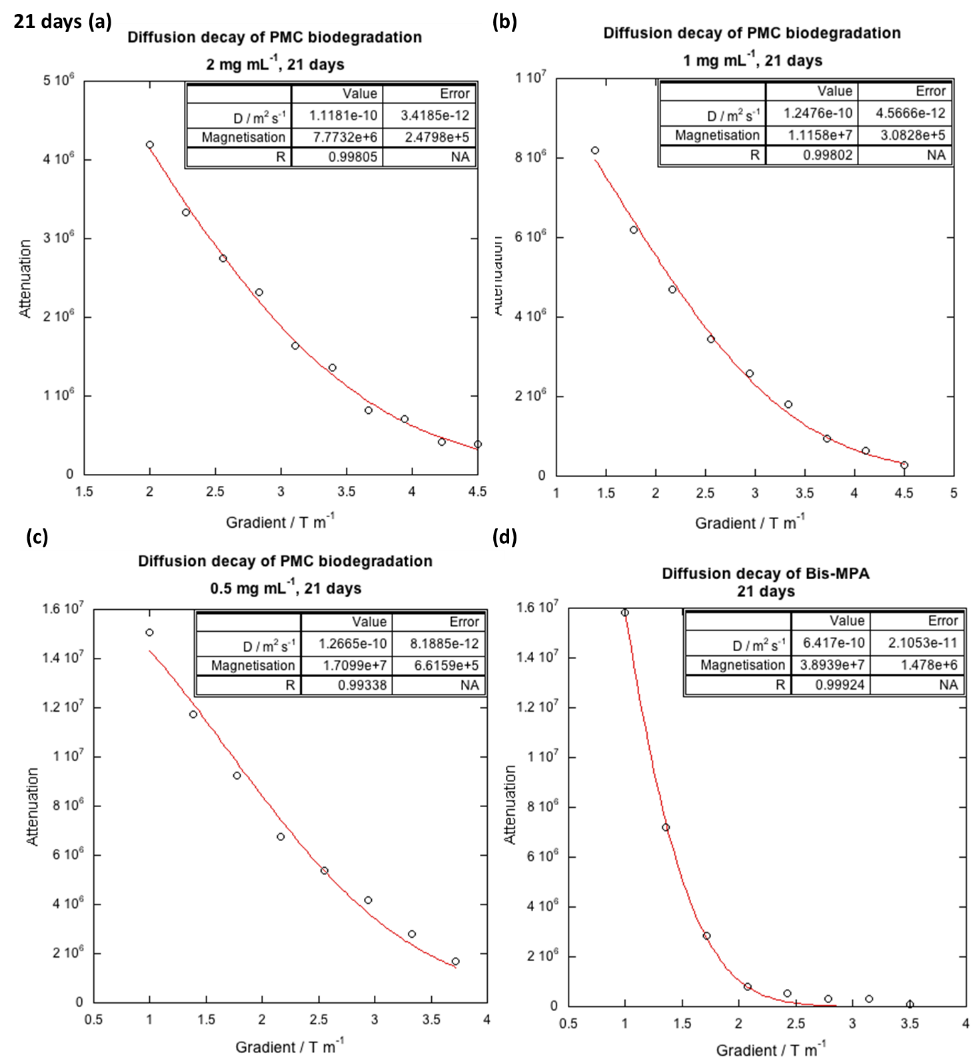
**

**
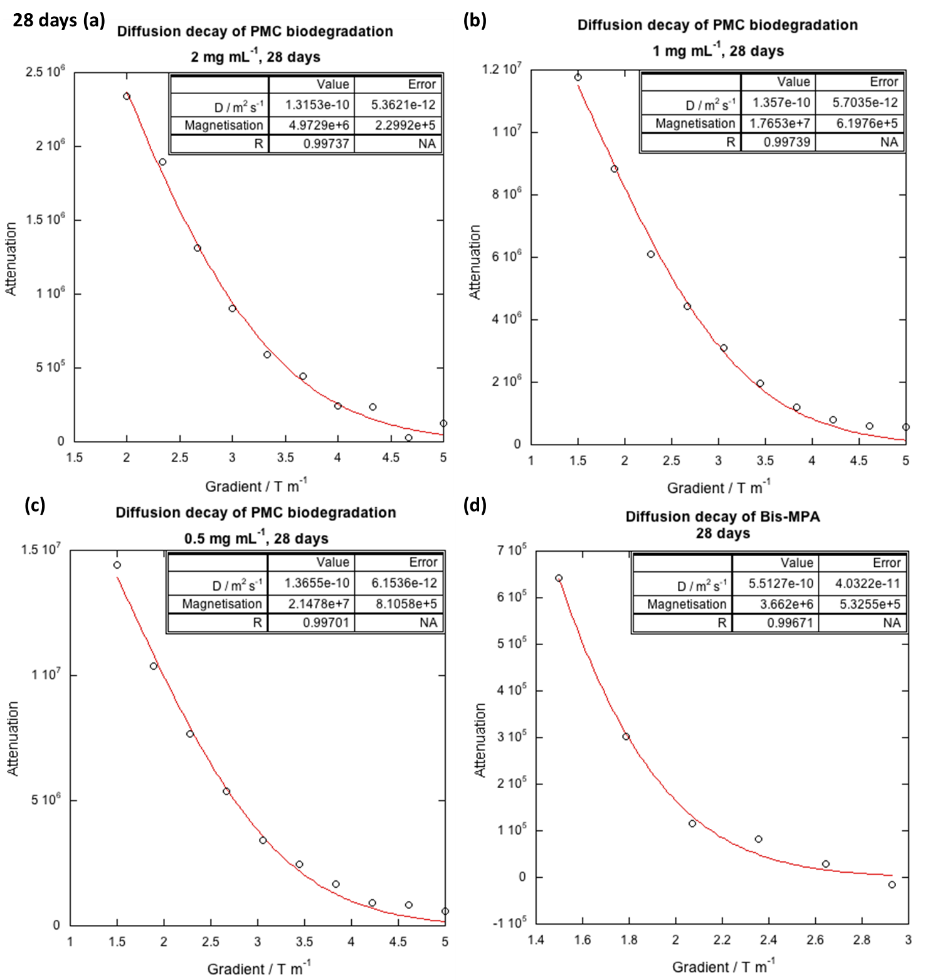
**

**
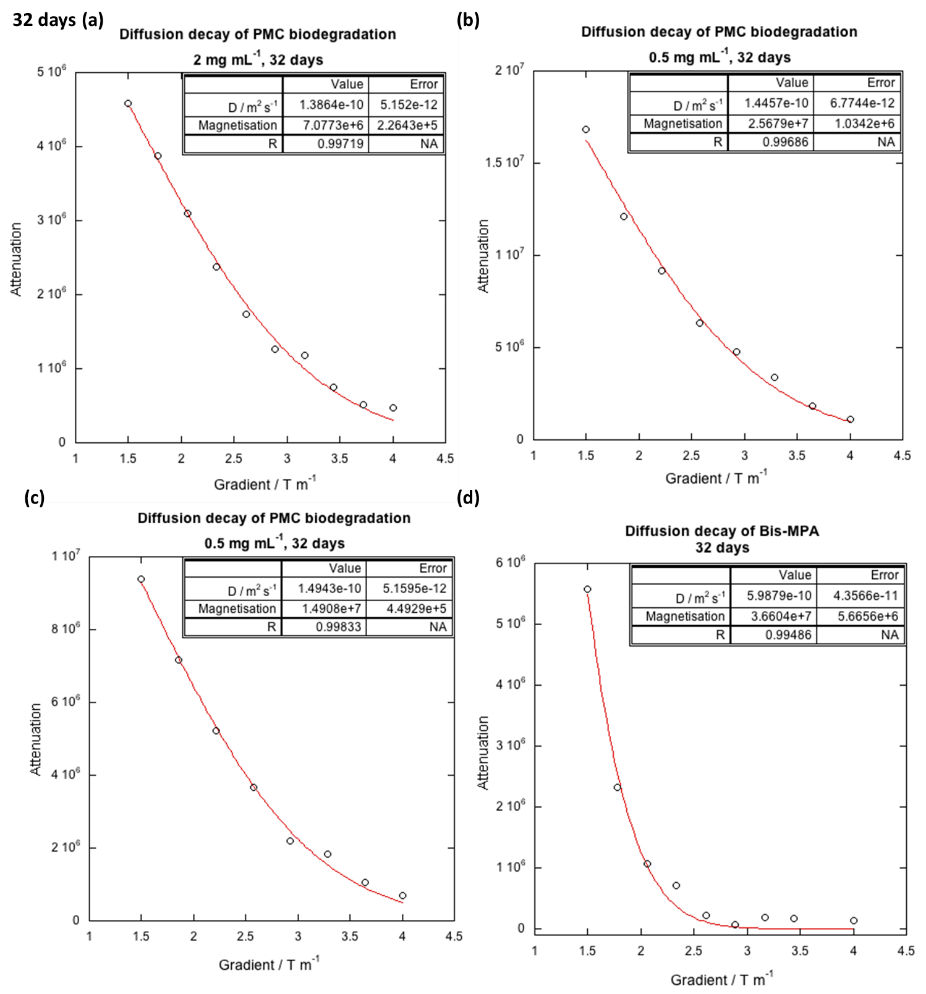
**

**
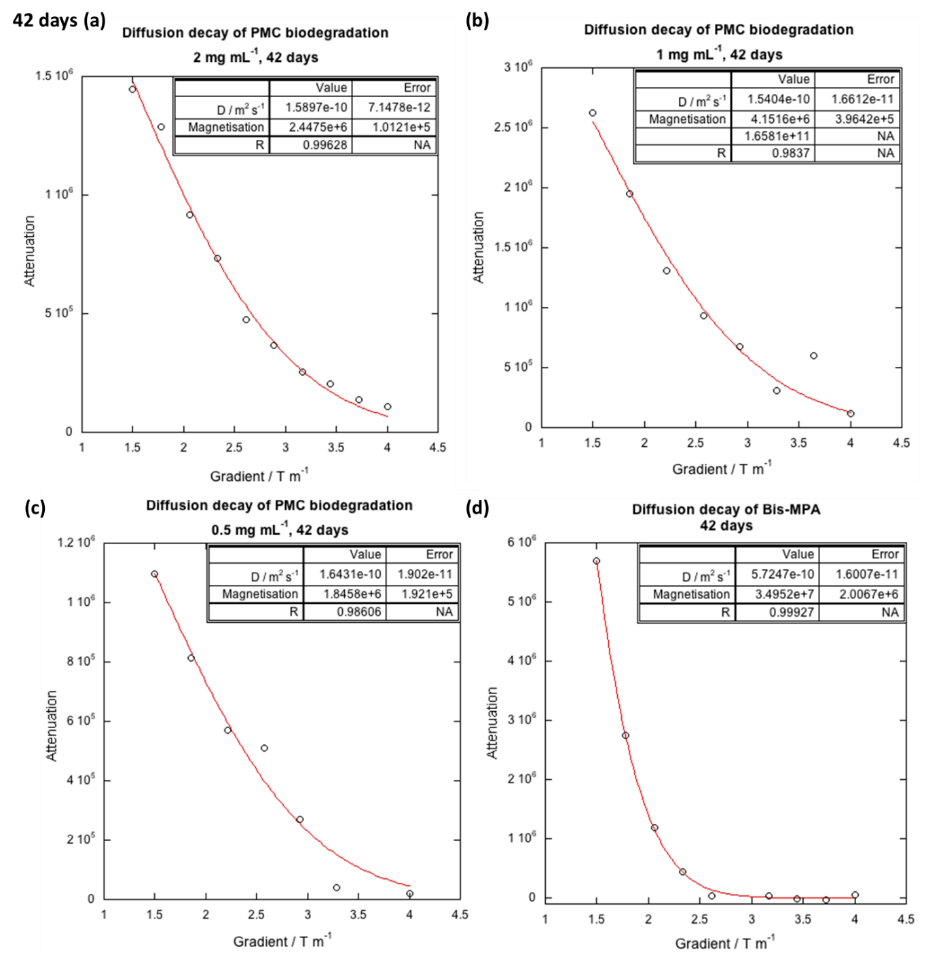
**

**PMC biodegradation 2 (*E. coli*, 0.05 M PBS (DI H2O), 37 °C, 180 rpm)**

Signal intensity decays for PMC at (a) 2 mg mL^–1^ (b) 1 mg mL^–1^ (c) 0.5 mg mL^–1^ and (d) bis-MPA at 2 mg mL^–1^.

**
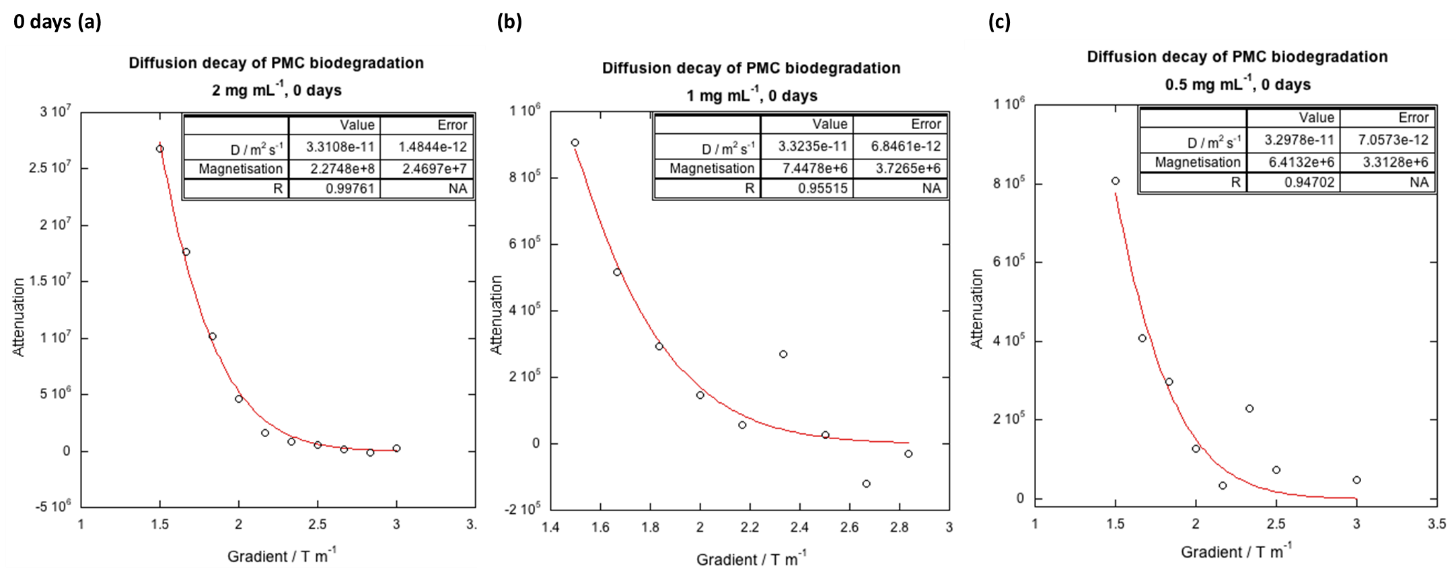
**

**
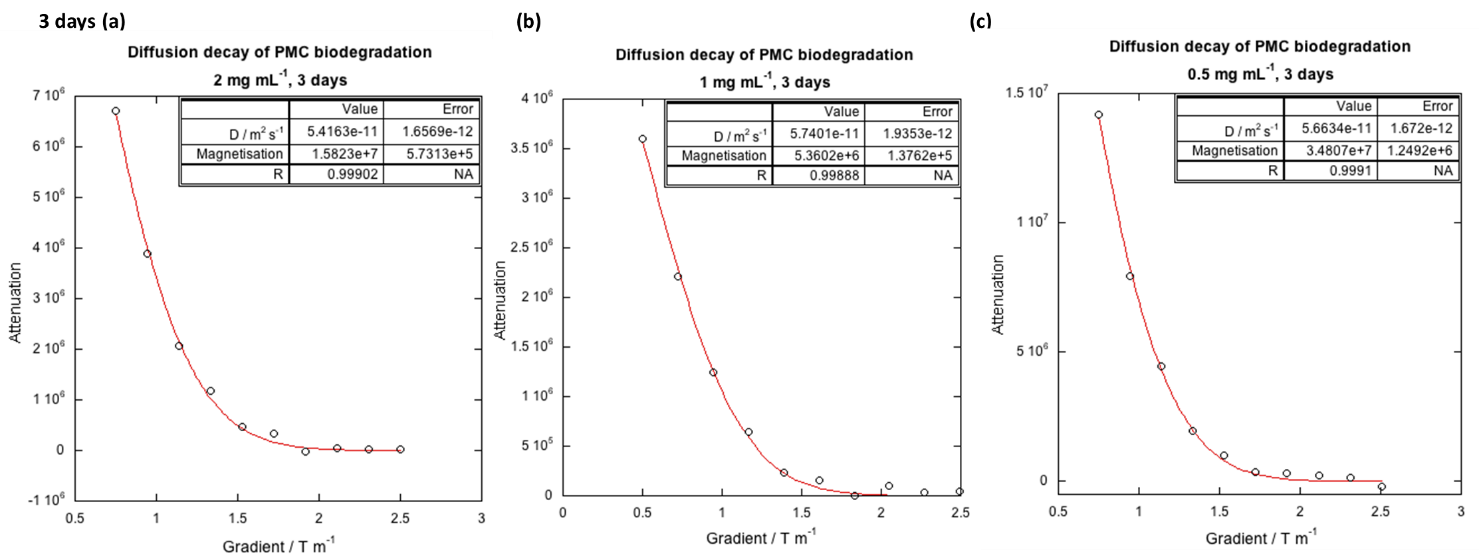
**

**
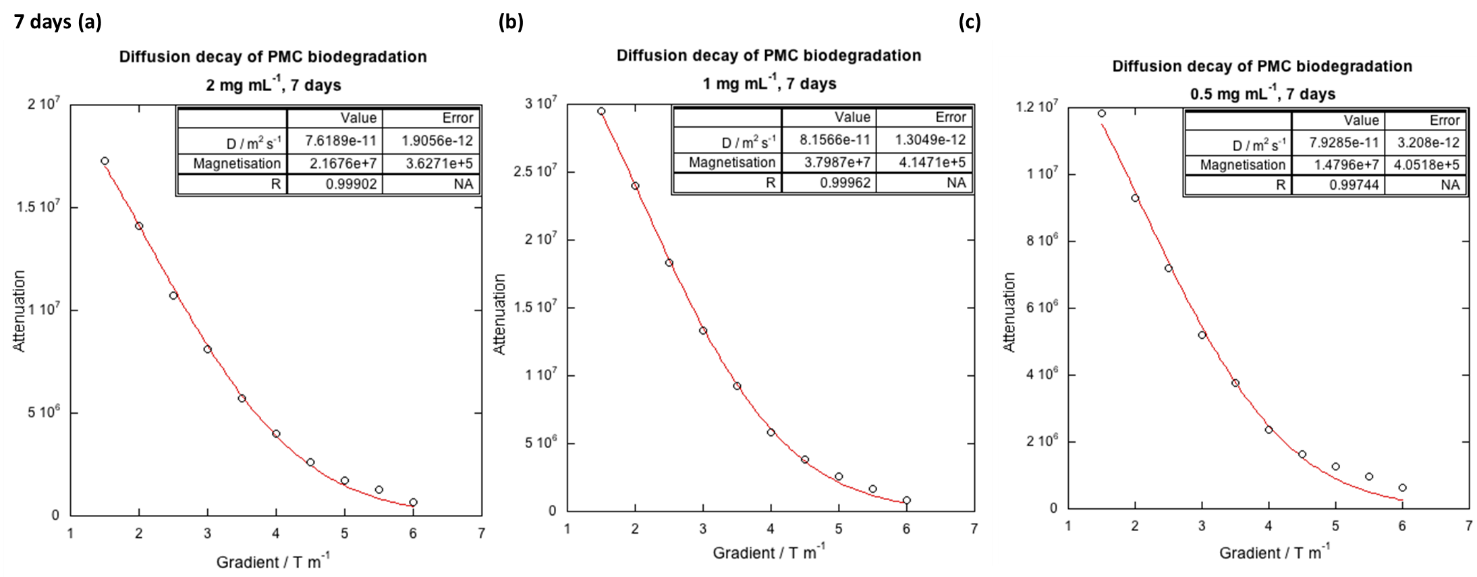
**

**
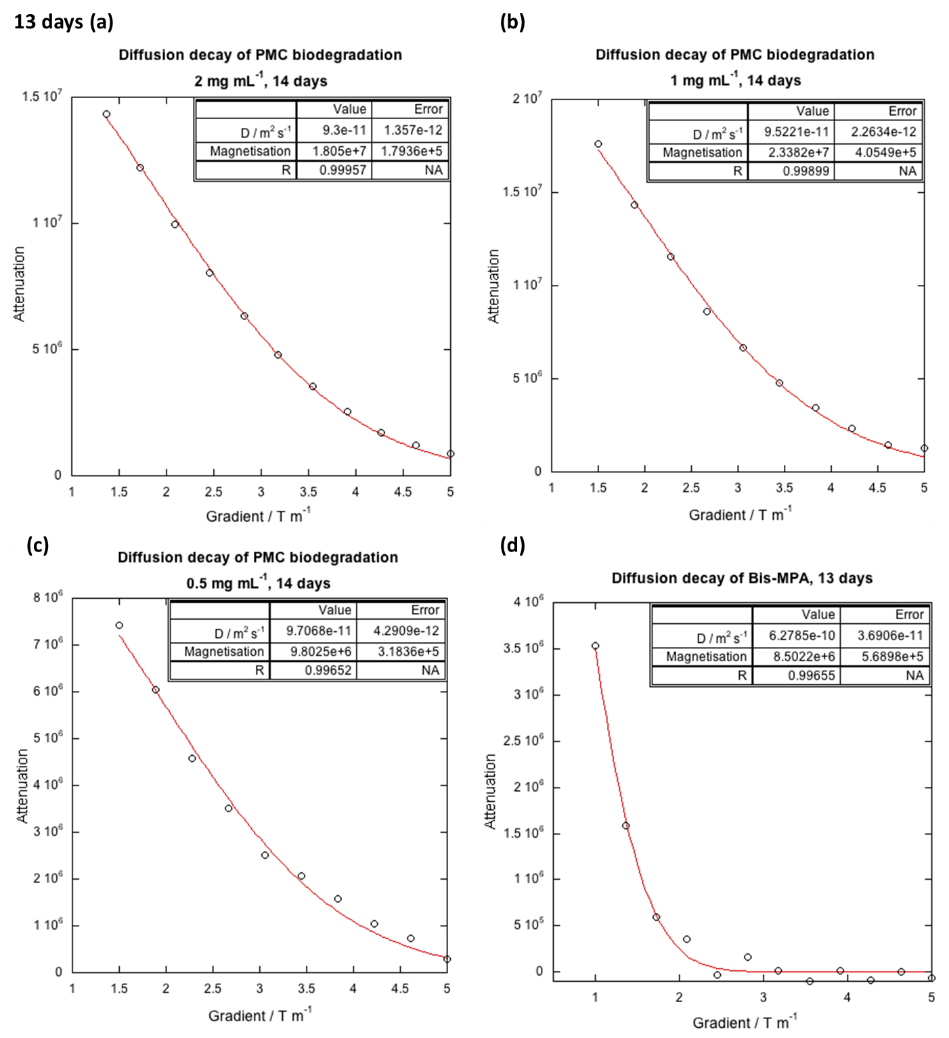
**

**
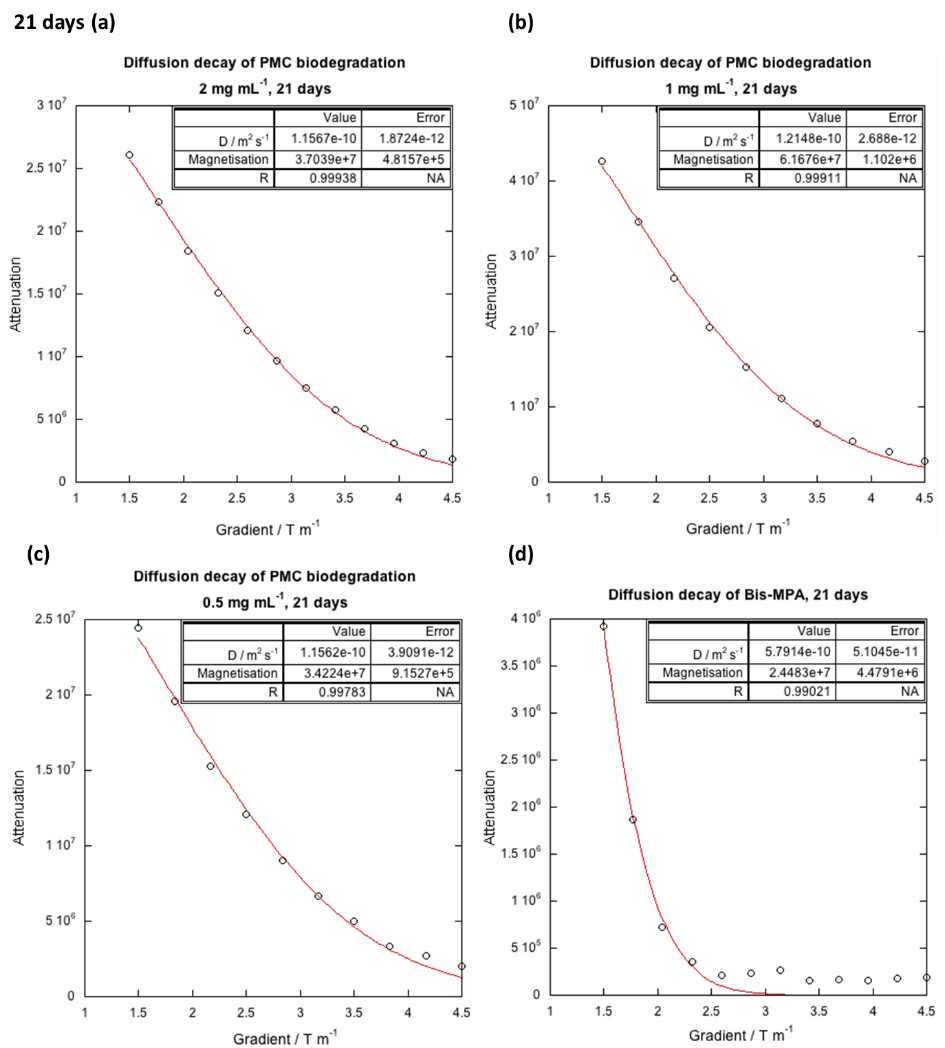
**

**
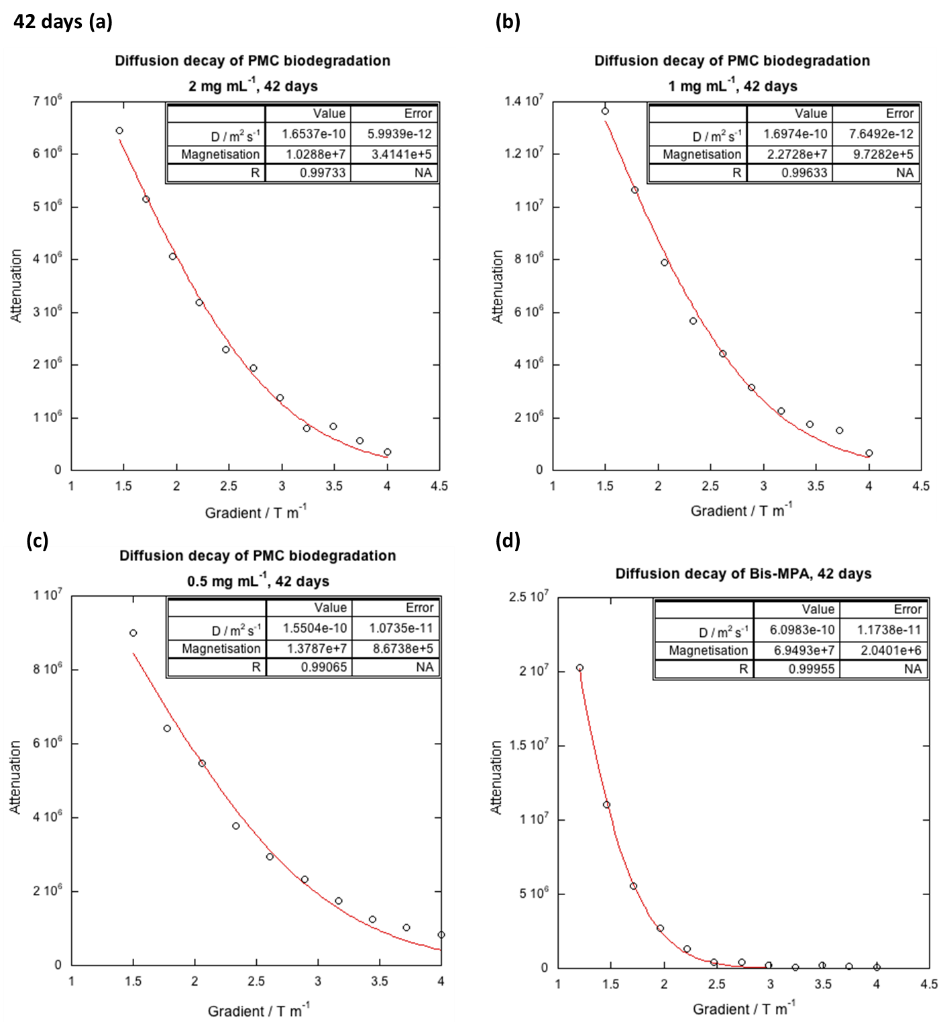
**

**
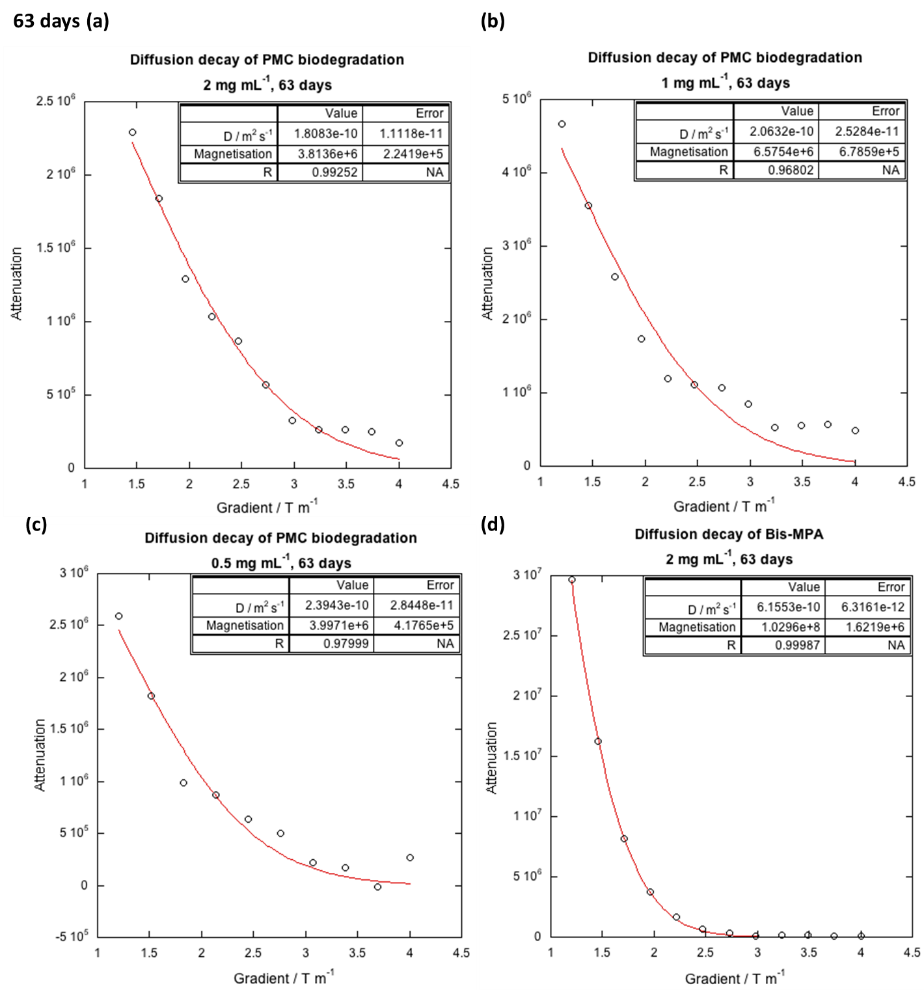
**


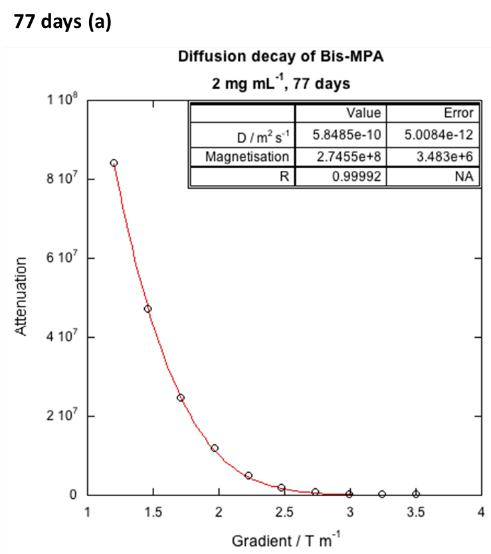


**PMC biodegradation (*E. coli*, 0.05 M PBS (DI H2O), 37 °C, 180 rpm) with continuous removal of degradation product**

Signal intensity decays for PMC at (a) 1 m·mL^–1^ (b) 0.5 mg·mL^–1^ and (c) 0.25
mg·mL^–1^.

**
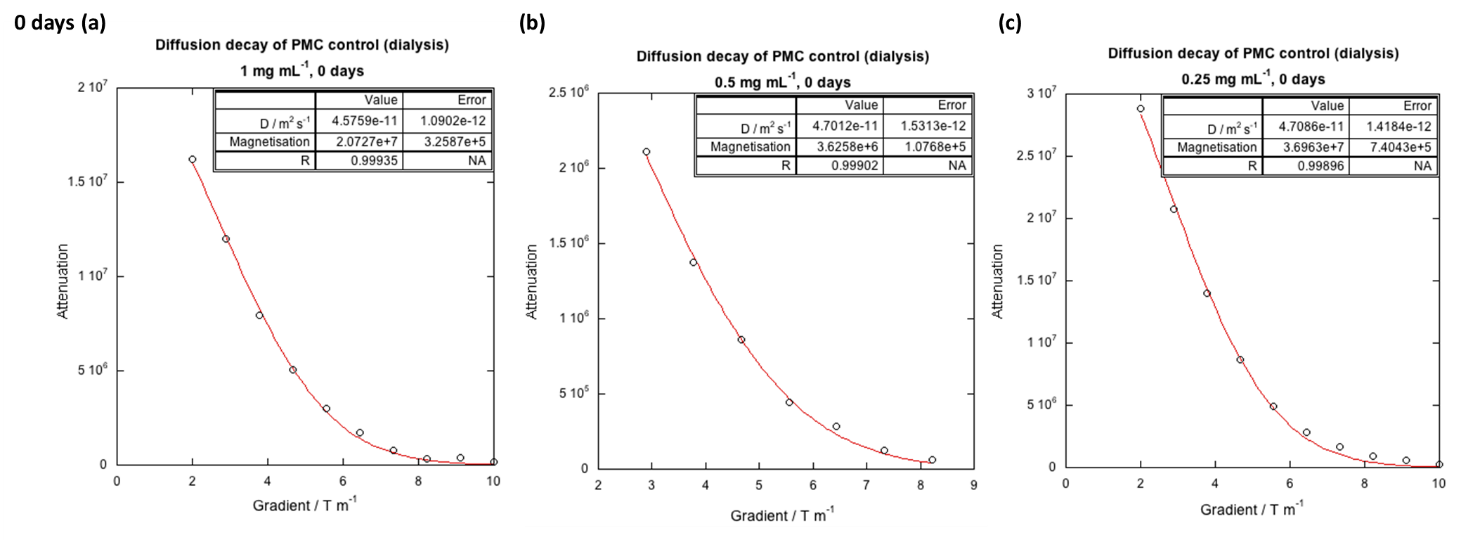
**

**
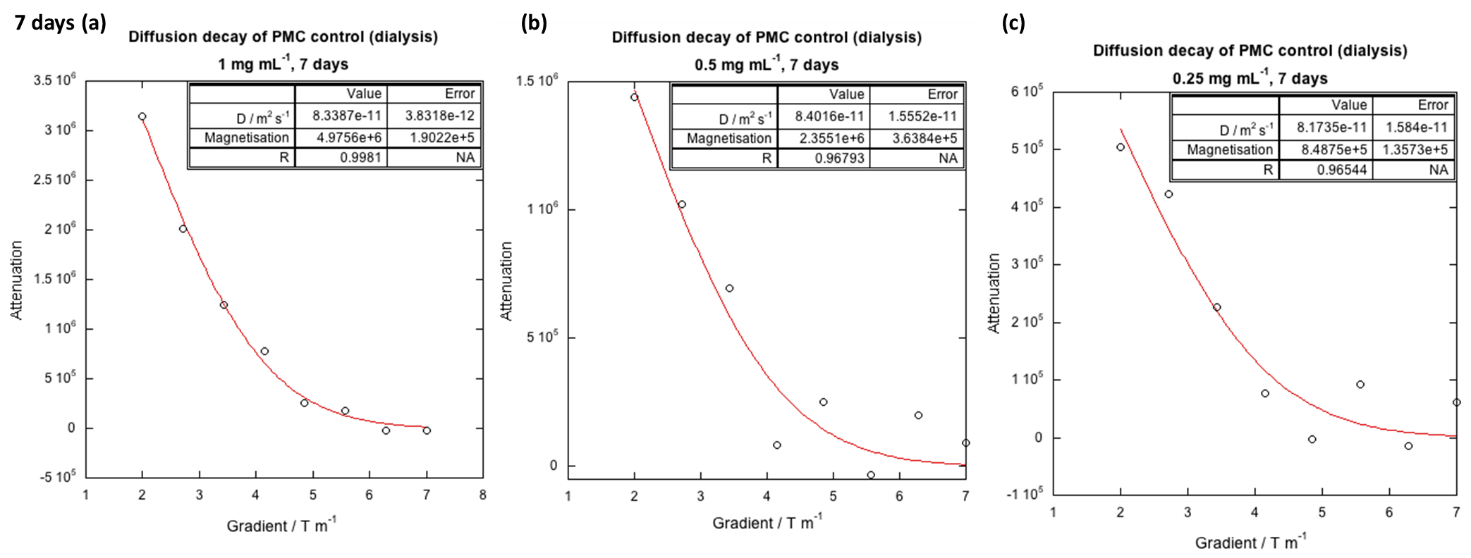
**

**
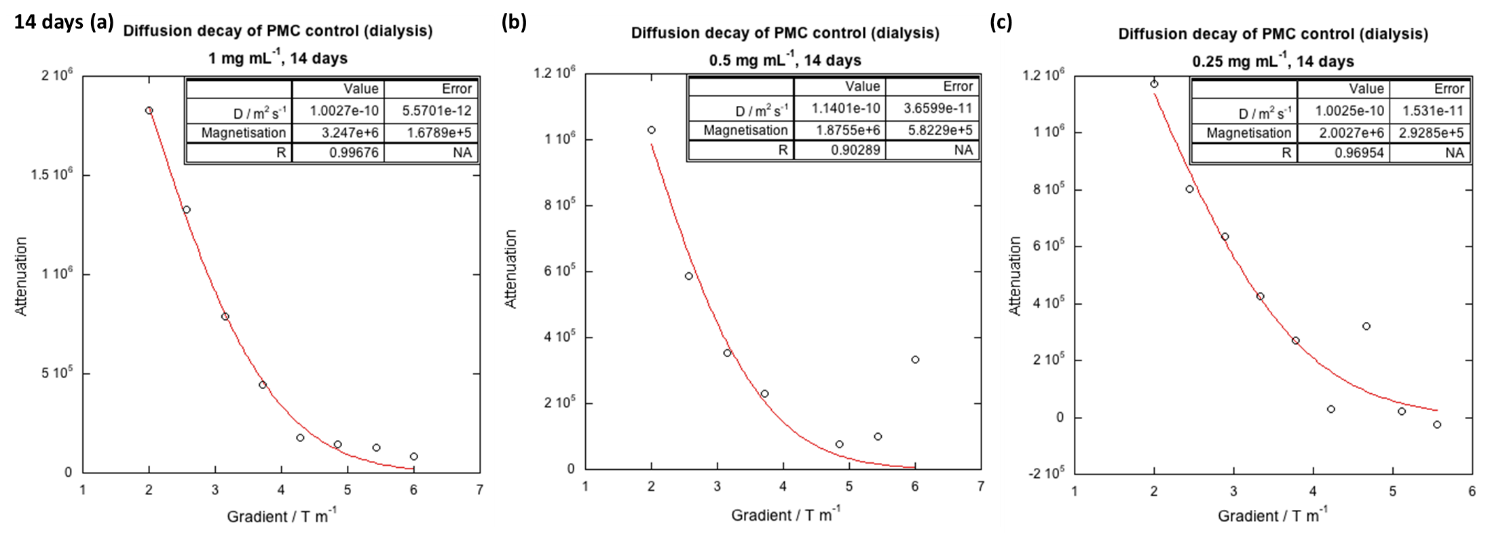
**

**
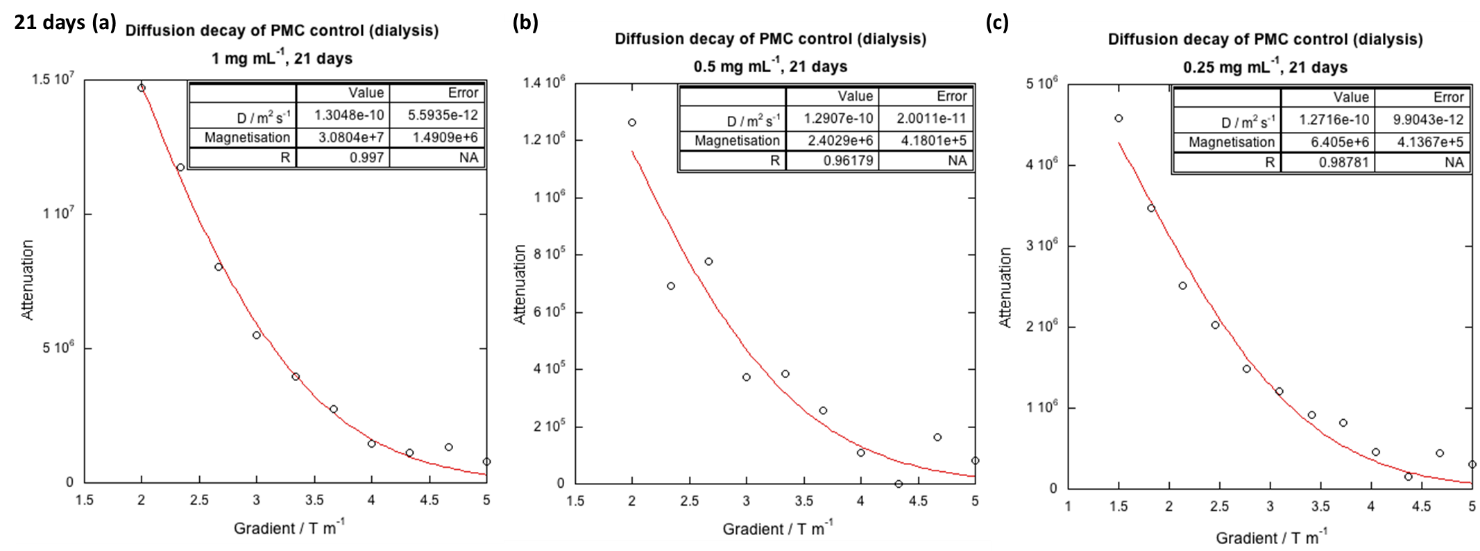
**

**PMC biodegradation mixed with PEG (*E. coli*, 0.05 M PBS (DI H2O), 37 °C, 180 rpm)**

Signal intensity decays for PMC at (a) 1 mg·mL^–1^ (b) 0.5 mg·mL^–1^ (c) 0.25 m· mL^–1^ and (d) Bis-MPA at 2 mg·mL^–1^.

**
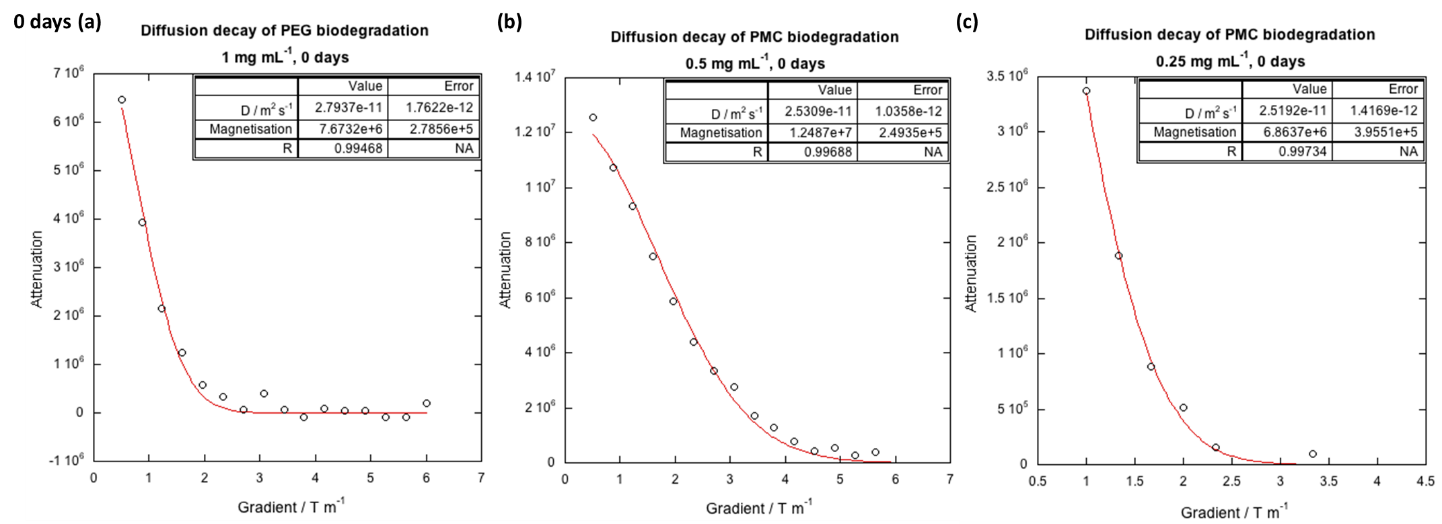
**

**
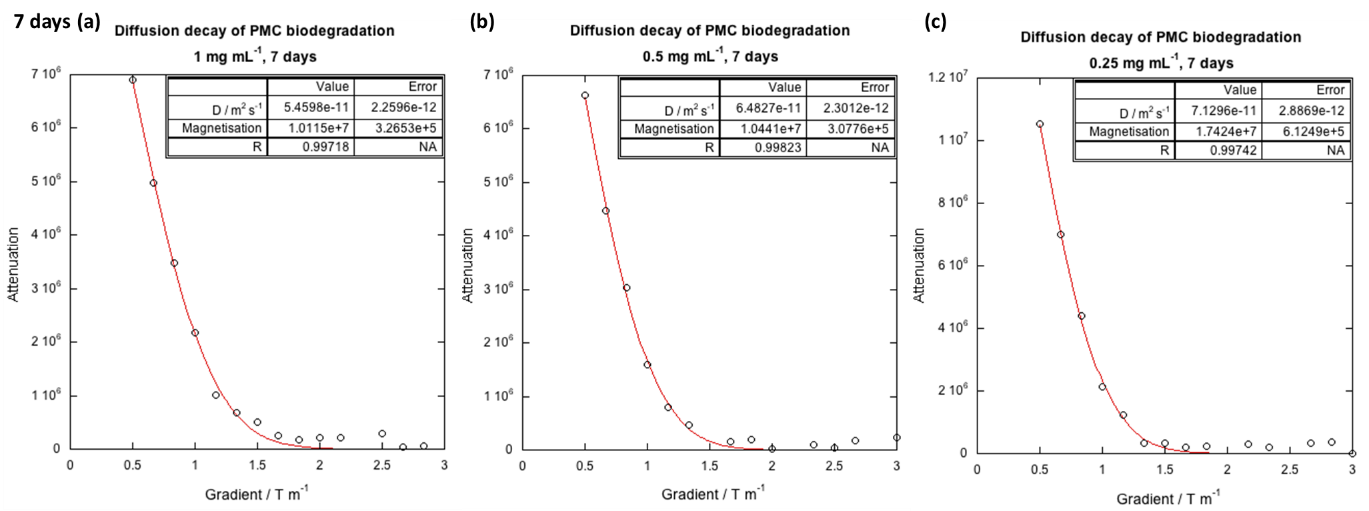
**

**
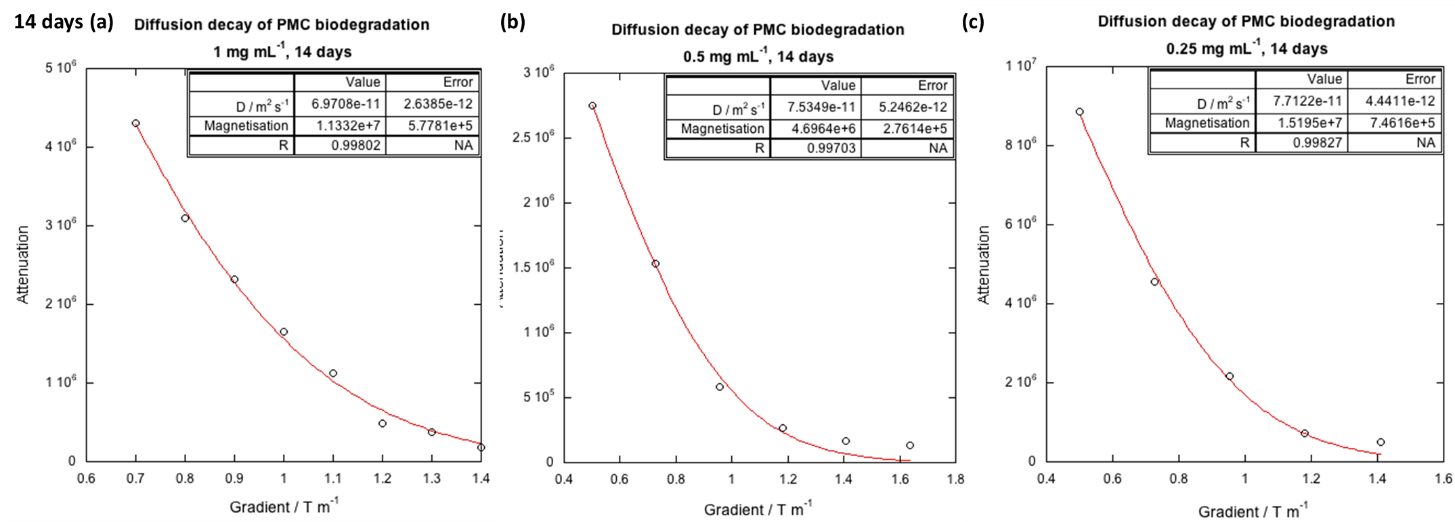
**

**
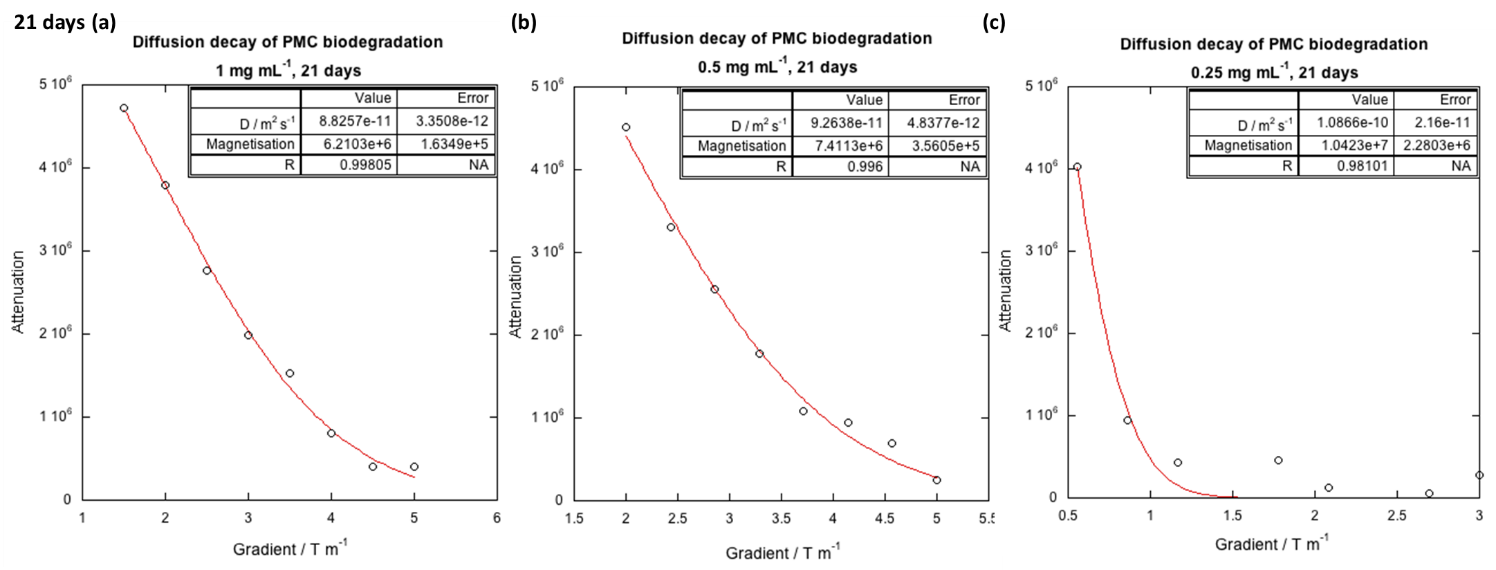
**

**
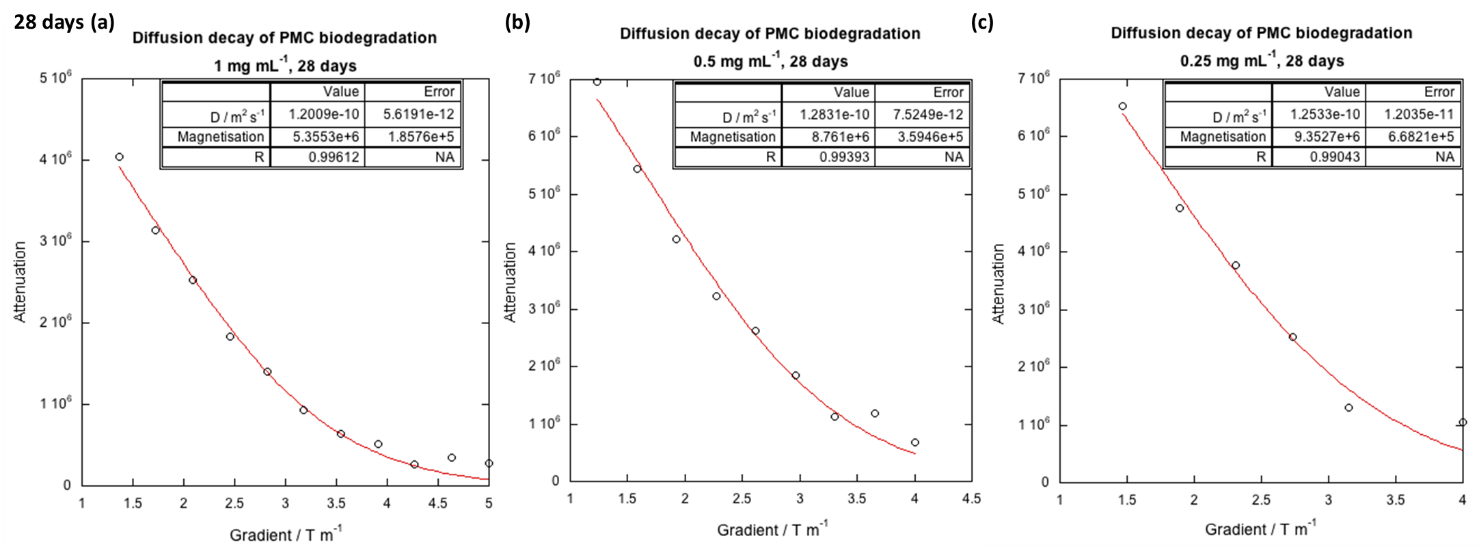
**


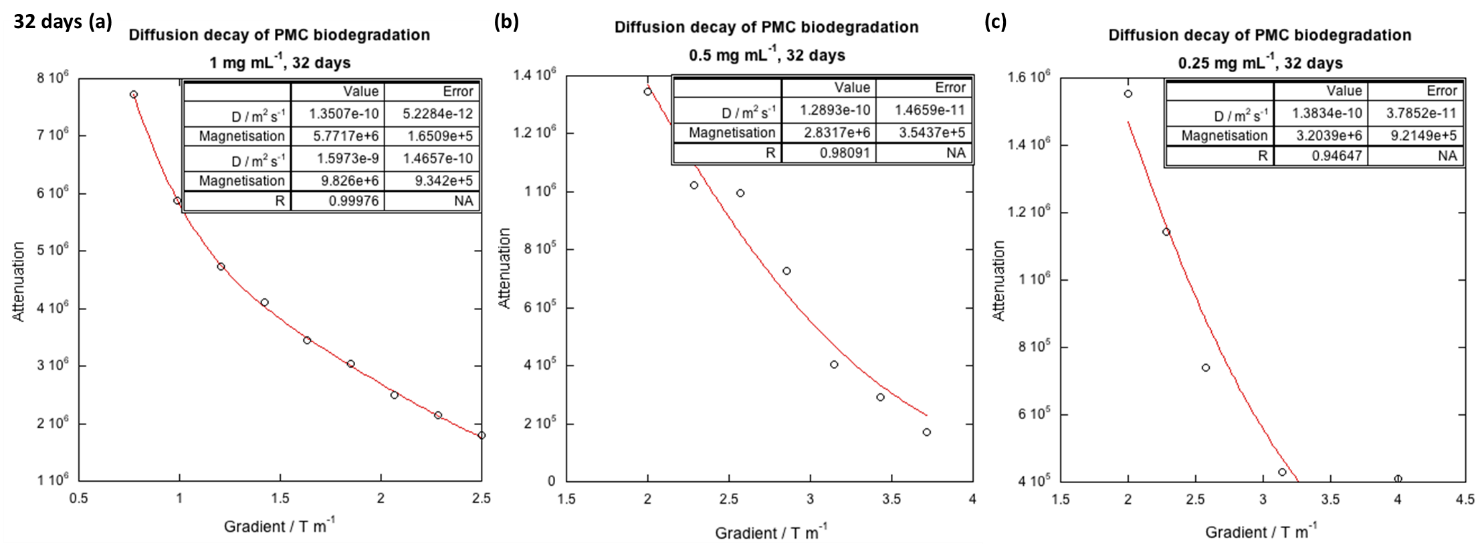


32 days (a) Biexponential fitting due to high water content, first 3 points removed due to water overpowering the polymer signal as a result of the low *G*_min_.

**
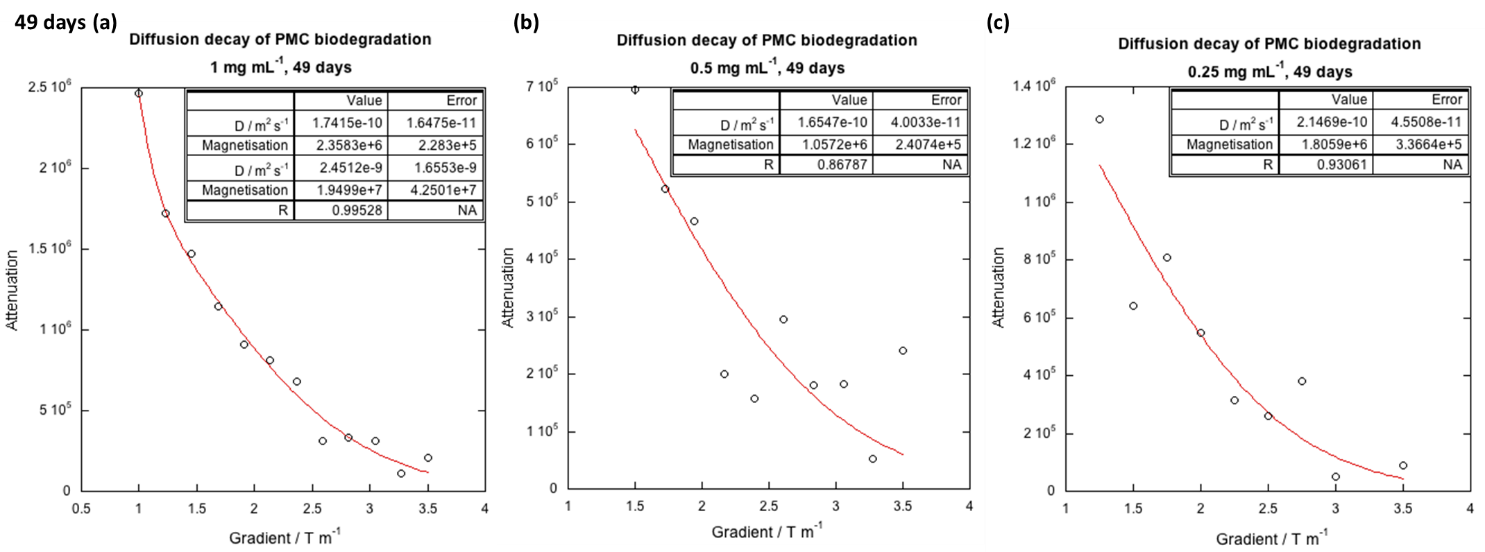
**

Signal intensity decays for PEG at (a) 1 mg·mL^–1^ (b) 0.5 mg·mL^–1^ (c) 0.25 mg·mL^–1^.

**
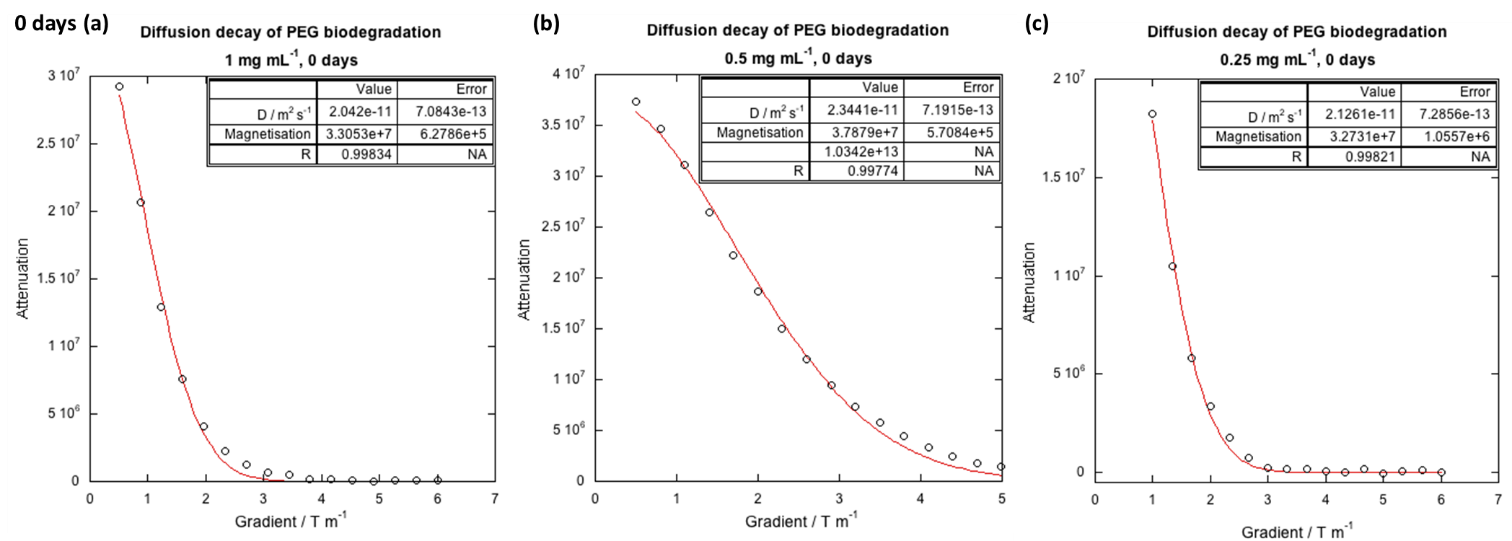
**

**
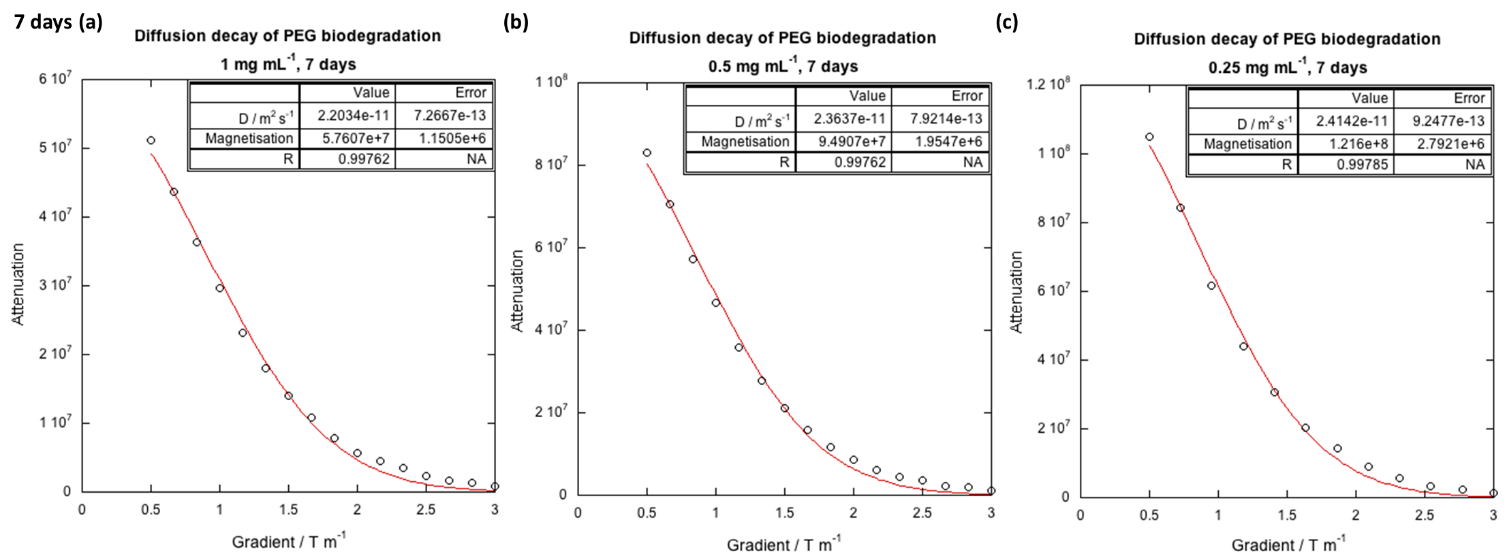
**

**
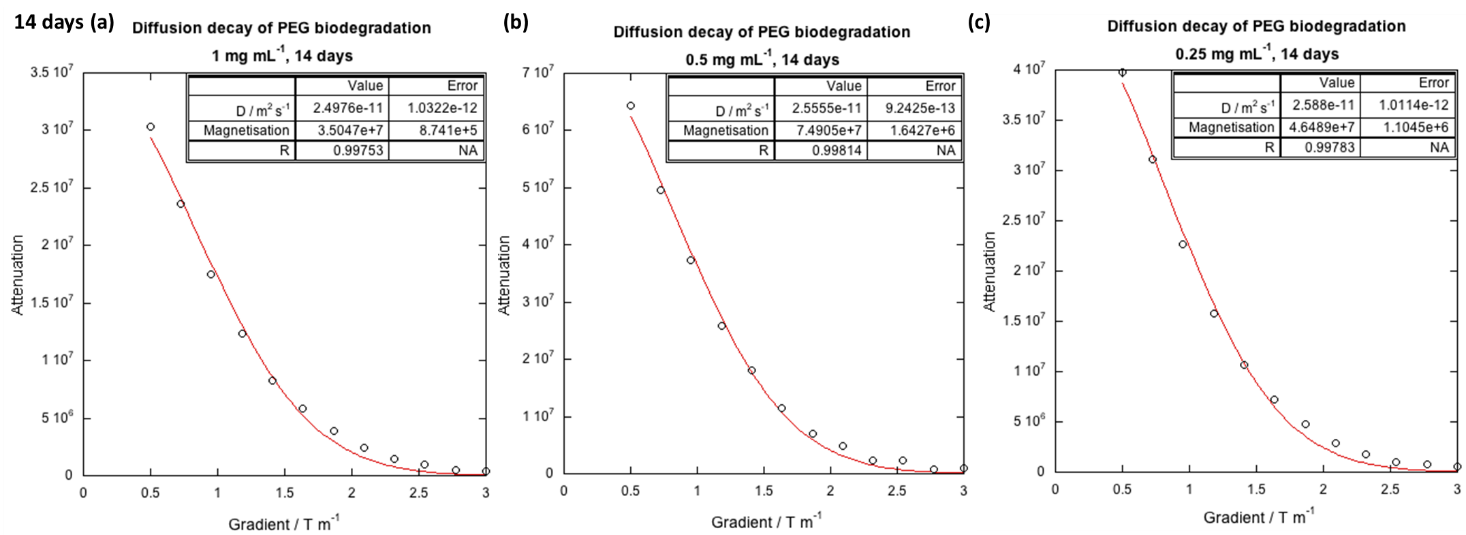
**

**
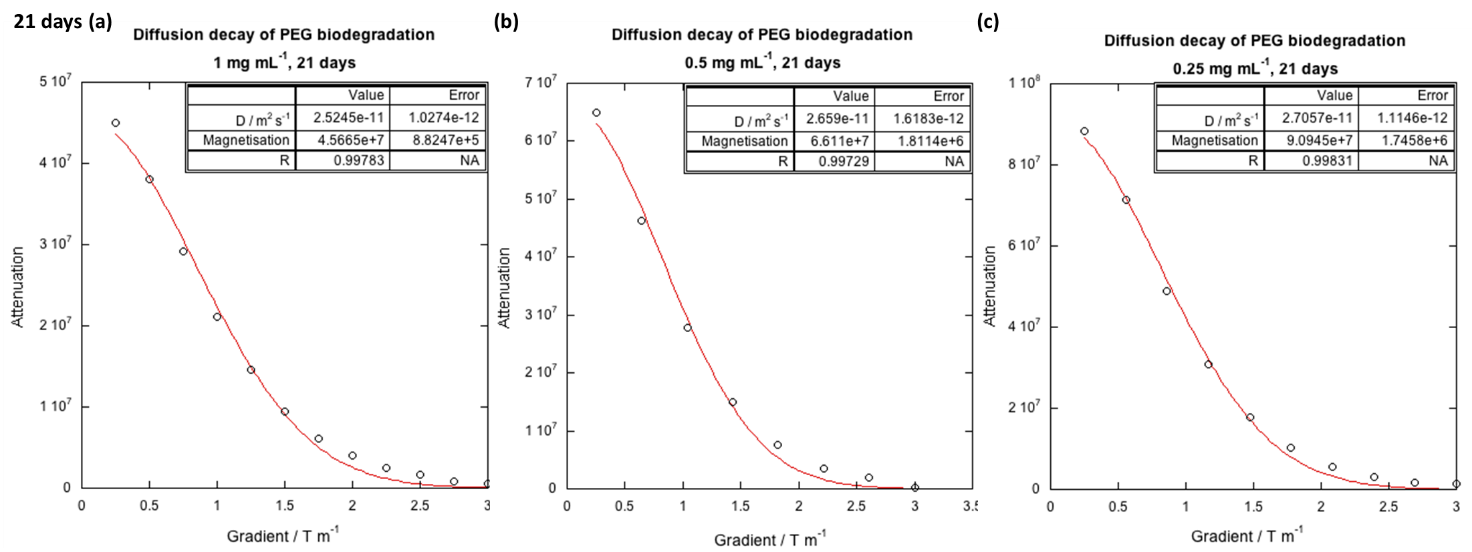
**

**
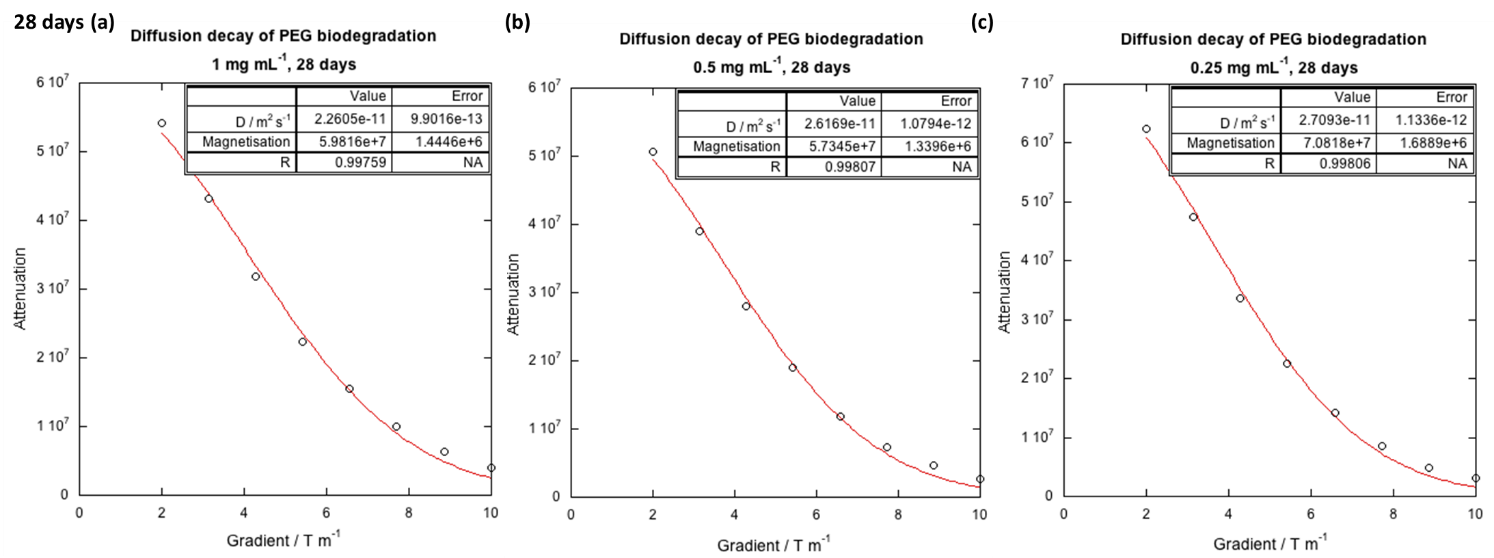
**

**
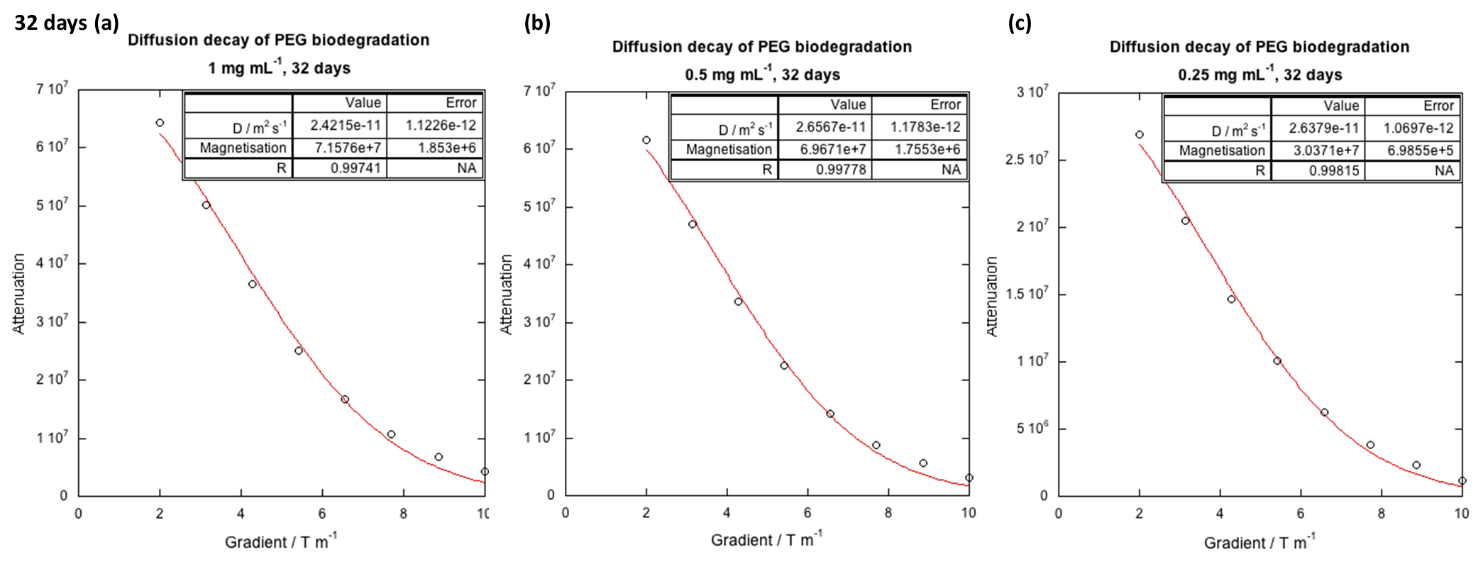
**

**
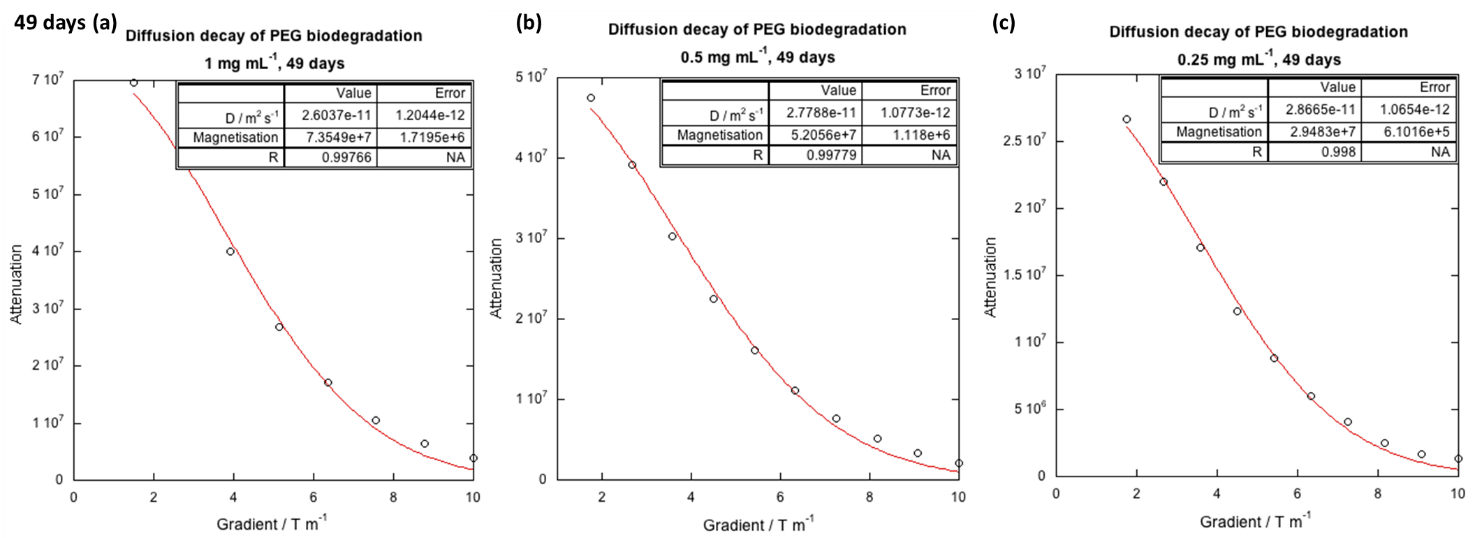
**

# **References**

[1] E. O. Stejskal, J. E. Tanner, “Spin diffusion measurements: spin echoes in the presence of a time‐dependent field gradient.“ *J. Chem. Phys.* **1965**, *42*, 288-292.

[2] C. C. Miller, “The Stokes-Einstein law for diffusion in solution.“ *Proc. R. Soc. Lond. A,* **1924**, *106*, 724-749.

[3] L. L. C. Gaylord Chemical Company. “DMSO Physical Properties” can be found at <https://www.gaylordchemical.com/products/literature/physical-properties/> (accessed on 21.07.2022)

[4] A. Chen, D. Wu, C. S. Johnson, “Determination of Molecular Weight Distributions for Polymers by Diffusion-Ordered NMR.” *J. Am. Chem. Soc.* **1995**, *117*, 7965-7970.

[5] W. Li, H. Chung, C. Daeffler, J. A. Johnson, R. H. Grubbs, “Application of (1)H DOSY for Facile Measurement of Polymer Molecular Weights.” *Macromolecules* **2012**, *45*, 9595-9603.

[6] P.-J. Voorter, A. McKay, J. Dai, O. Paravagna, N. R. Cameron, T. Junkers, “Solvent-Independent Molecular Weight Determination of Polymers Based on a Truly Universal Calibration.” *Angew. Chem., Int. Ed.* **2022**, *61*, e202114536.

[7] J. A. Wilson, S. A. Hopkins, P. M. Wright, A. P. Dove, “‘Immortal’ ring-opening polymerization of ω-pentadecalactone by Mg(BHT)2(THF)2” *Polym. Chem.* **2014**, *5*, 2691-2694.

[8] H. Ihre, A. Hult, J. M, J. Fréchet, I. Gitsov, “Double-Stage Convergent Approach for the Synthesis of Functionalized Dendritic Aliphatic Polyesters Based on 2,2-Bis(hydroxymethyl)propionic Acid.” *Macromolecules*, **1998**, *31*, 4061- 4068.

[9] D. P. Sanders, K. Fukushima, D. J. Coady, A. Nelson, M. Fujiwara, M. Yasumoto, J. L. Hedrick, “Simple and Efficient Synthesis of Functionalized Cyclic Carbonate Monomers Using a Versatile Pentafluorophenyl Ester Intermediate.” *J. Am. Chem.* **2010**, *132*, 14724-14726.

[10] P. S. Tofts, D. Lloyd, C. A. Clark, G. J. Barker, G. J. M. Parker, P. McConville, C. Baldock, J. M. Pope, “Test liquids for quantitative MRI measurements of self-diffusion coefficient in vivo.” *Magn. Reson. Med.* **2000**, *43*, 368-374.
